# C−H Borylation/Cross‐Coupling Forms Twisted Donor–Acceptor Compounds Exhibiting Donor‐Dependent Delayed Emission

**DOI:** 10.1002/chem.201801799

**Published:** 2018-06-25

**Authors:** Daniel L. Crossley, Pakapol Kulapichitr, James E. Radcliffe, Jay J. Dunsford, Inigo Vitorica‐Yrezabal, Rachel J. Kahan, Adam W. Woodward, Michael L. Turner, Joseph J. W. McDouall, Michael J. Ingleson

**Affiliations:** ^1^ School of Chemistry The University of Manchester Oxford Road Manchester M13 9PL UK

**Keywords:** benzothiadiazole, boron, borylation, natural transition orbitals, thermally activated delayed fluorescence

## Abstract

Benzothiadiazole (BT) directed C−H borylation using BCl_3_, followed by B−Cl hydrolysis and Suzuki–Miyaura cross‐coupling enables facile access to twisted donor–acceptor compounds. A subsequent second C−H borylation step provides, on arylation of boron, access to borylated highly twisted D−A compounds with a reduced bandgap, or on B−Cl hydrolysis/cross‐coupling to twisted D‐A‐D compounds. Photophysical studies revealed that in this series there is long lifetime emission only when the donor is triphenylamine. Computational studies indicated that the key factor in observing the donor dependent long lifetime emission is the energy gap between the S_1_/T_2_ excited states, which are predominantly intramolecular charge‐transfer states, and the T_1_ excited state, which is predominantly a local excited state on the BT acceptor moiety.

## Introduction

**Figure 1:**
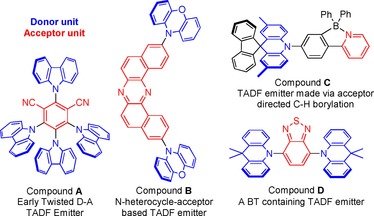
Select examples of twisted D−A molecules that exhibit TADF, including examples containing a boracycle (compound **C**) and a benzo[*c*]‐1,2,5‐thiadiazole (BT) acceptor unit (compound **D**).

Non‐planar, twisted, aromatic compounds containing donor (D=donor) and acceptor (A=acceptor) units are attracting considerable current interest for application in organic light emitting diodes (OLEDs).^1^ These molecules show thermally activated delayed fluorescence (TADF),^2^ a phenomenon that can dramatically increase the power efficiency of transition metal free OLEDs by effective harvesting of triplet excitons. Twisted aromatic structures have a significant dihedral angle between the D and A units (see Figure 1 for select examples) that can lead to a large degree of spatial separation of the HOMO and LUMO.^3^ This results in a small energy difference between the lowest energy excited singlet and triplet states (Δ*E*_S−T_) as it reduces the magnitude of exchange.^1^, ^2^, ^4^ Invoking Marcus′ theory,^5^ the rate of (reverse) intersystem crossing (*k*_(R)ISC_) is given by Equation (1) from which the importance of Δ*E*_S−T_ can be seen to dominate $k_{\left(R\right)ISC}$ , as it enters quadratically into the exponential term.

(1) $$k_{\left(R\right)ISC}=2\;\pi {\left|\langle \Psi_{S_{1}}|\hat{H}|\Psi_{T_{1}}\rangle \right|}^{2}\frac{1}{\sqrt{4\;\pi \lambda T}}e^{-\left[\frac{1}{4T}\left(\lambda +2\Delta E_{S-T}+\frac{{\Delta E}_{\;\;S-T}^{2}}{\lambda }\right)\right]}$$

Smaller Δ*E*_S−T_ values increase the rate of intersystem crossing (forward and reverse) even for the low spin‐orbit coupling interactions present in heavy atom‐free molecules. The more rapid intersystem crossing results in effective delayed emission from the singlet state provided that the emissive molecules have good photoluminescence quantum yields (PLQY). However, the necessity to balance multiple factors to achieve efficient TADF emission^6^ often requires significant molecular fine tuning. Therefore, methods to rapidly make libraries of twisted D−A molecules are important in facilitating the identification of materials that are potential TADF emitters.

**Scheme 1:**
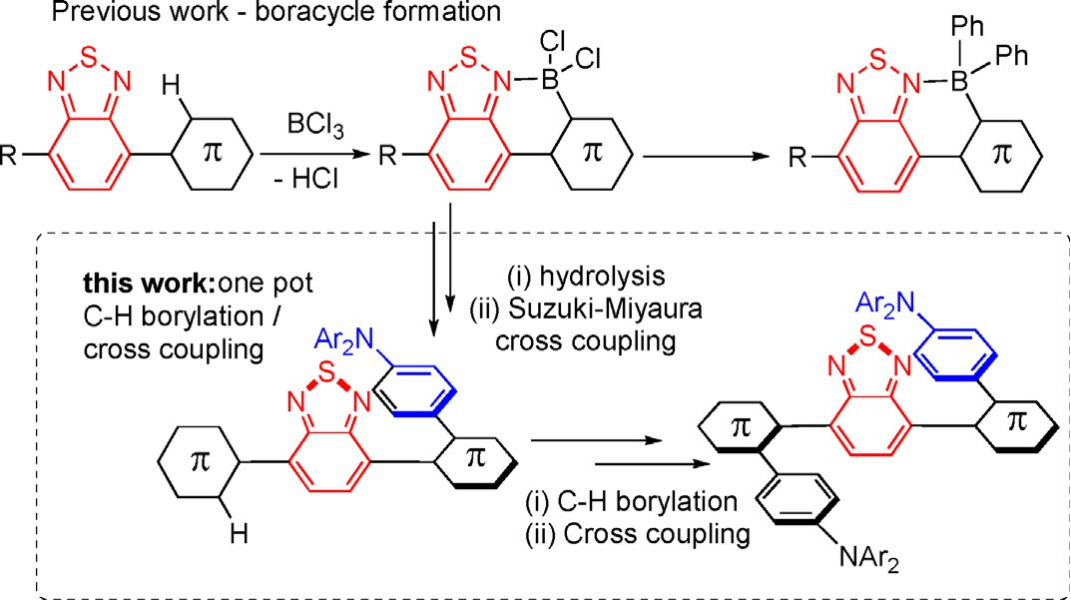
Top, previous work and inset, this work, one pot electrophilic C−H borylation/ cross‐coupling to form twisted D−A and D‐A‐D compounds.

The incorporation of steric bulk proximal to the donor‐acceptor linkage is one way to impart a significant dihedral angle between the D and A units.1a,1b We hypothesised that a one‐pot two step sequence of directed electrophilic C−H borylation and Suzuki–Miyaura cross‐coupling would be a simple method to rapidly form highly twisted D−A molecules (Scheme 1). Many acceptor units are electron deficient N‐heterocycles (see compounds **B**–**D**)^7^ that possess a Lewis basic site that can direct electrophilic C−H borylation by coordination to a Lewis acidic borane (e.g. BCl_3_).^8^ Post C−H borylation the cross‐coupling step will occur at a position that creates significant steric crowding and thus will lead to twisting in the D−A molecule. While directed electrophilic C−H borylation has been utilised to incorporate boracycles into D−A compounds to reduce the LUMO energy, including in TADF emitters (compound **C**),7b, 9 the combination of directed electrophilic C−H borylation with sequential Suzuki–Miyaura cross‐coupling is much less explored,^10^ and to date has not been used to make highly twisted D−A compounds to the best of our knowledge. Our previous work used benzo[*c*]‐1,2,5‐thiadiazole (BT) as the acceptor moiety to direct C−H borylation (Scheme 1)8b and TADF emitters have been reported that include BT as the acceptor moiety (e.g. compound **D**);7c, 11 however when compared to other electron deficient N‐heterocycles, the BT moiety is underutilised.^1^, 7c In this work we report the development of one‐pot BT directed C−H borylation/cross‐coupling to make a range of twisted D−A and D‐A‐D compounds. A number of these compounds display long lifetime emission which was found to be highly dependent on the nature of the donor. Computational studies revealed that this disparity principally derives from variation in the energy of the S_1_ and T_2_ excited states, which are predominantly intramolecular charge‐transfer states and thus are highly dependent on the frontier orbital energy of the donor moiety.

## Results and Discussion

**Scheme 2:**
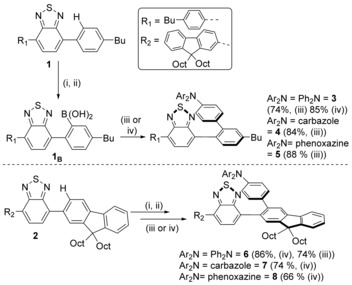
The synthesis of **3**–**8**. (i) 2–3 equiv BCl_3_ (1 m in CH_2_Cl_2_), 3 h; (ii) solvent removed, dissolved in 10:1 THF/H_2_O, 16 h; (iii)+electrophile (1.1 equiv), degassed, +Pd(PPh_3_)_4_ in THF and K_3_PO_4_ 2 m (aq.), reflux; (iv)+electrophile (1.1 equiv), degassed, then +Pd(*t*Bu_3_P)_2_ in THF and K_3_PO_4_ 2 m (aq.), 15 h.

Precursors, **1** and **2** (Scheme 2), were synthesized using standard cross‐coupling methodologies. Compound **2** was selected as 9,9‐dioctyl‐fluorene‐BT derivatives generally have good PLQY values,8b however as derivatives of **2** are often not amenable to crystallisation compound **1** was also employed as this should give more crystalline products. BT directed C−H borylation of **1** and **2** was achieved using BCl_3_ in dry CH_2_Cl_2_, this was followed by a solvent switch to THF/H_2_O (10:1), which led to B−Cl hydrolysis over 16 h at ambient temperature. The putative boronic acids, **1_B_** and **2_B_**, were not isolated but instead used directly in Suzuki–Miyaura cross‐coupling reactions with a range of halogenated arylamines. The Suzuki–Miyaura cross‐coupling reactions could be performed at ambient temperature using Pd(P*t*Bu_3_)_2_ or at raised temperature using Pd(PPh_3_)_4_, with both procedures giving good yields of the D−A products (for example isolated yields for **3** of 85 and 74 %, respectively, were obtained after purification). Arylamines utilised included, 4‐bromotriphenylamine, 9‐(4‐bromophenyl)carbazole, and 10‐(4‐bromophenyl)phenoxazine, as these install donor units commonly found in twisted D−A compounds that exhibit TADF,^1^ including in examples containing four‐coordinate boron units.7c

**Figure 2:**
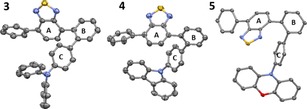
Solid state structures of **3**–**5**, ellipsoids at 50 % probability. Hydrogen atoms and *n*‐butyl groups omitted for clarity. Angle between planes of specific rings for **3**: A−B=60.8° and B−C=48.2° for **4**: A−B=70.1° and B−C=61.3°; for **5**: A−B=59.3° and B−C=53.4°.

The twisted D−A compounds **3**–**8** were characterized by multinuclear NMR spectroscopy and mass spectrometry. Compounds **3**–**8**, readily accessed by benzothiadiazole directed borylation, represent substitution patterns that are hard to access by conventional methods for example, 2,3‐disubstituted fluorene structures (other synthetic strategies generally lead to 2,7‐disubstituted fluorenes for example). In our hands, **3**–**5** were amenable to crystallization and single crystal X‐ray diffraction studies; however, compounds **6**–**8** were not. The solid‐state structures of compounds **3**–**5** confirm the expected highly twisted structures, with significant angles between the planes of ring A and B and between rings B and C in each case (Figure 2). These angles are closely comparable to the optimised structures from DFT calculations on model compounds of **3**–**5** (where *n*‐butyl has been replaced with hydrogen, termed **3_H_**‐**5_H_**). These calculations were performed using the PBE0 exchange‐correlation functional with the 6‐31G(d,p) basis set. The effect of solvent was included using the polarisable continuum model (PCM) for toluene. We denote this level of theory as PBE0/6‐31G(d,p)/PCM(toluene). These calculations revealed that the LUMO is predominantly localised on the BT acceptor unit with a minor contribution from the carbon atoms in ring B in each case (comparable to other twisted BT based compounds).7c The HOMOs are also similar in nature, predominantly localised on the Ph‐NAr_2_ units. However, it is notable that for **3_H_** and **4_H_**, the HOMO also has a minor contribution from ring B (Figure S29), whereas with the phenoxazine derivative **5_H_** there is effectively zero contribution from ring B (Figure S32) to the HOMO. Thus, in compound **5_H_** there is more complete spatial separation of the HOMO and LUMO. We attribute this disparity to the higher energy frontier orbitals of the phenoxazine donor unit which leads to the HOMO being more localised onto the donor NAr_2_ unit. This can be quantified by determining the absolute HOMO–LUMO spatial overlap percentages which for **3_H_** and **4_H_** are 15.3 and 17.2 % but for **5_H_** the absolute spatial HOMO–LUMO overlap is 3.2 %. Thus, as the HOMO–LUMO energy gap decreases due to an increase in the HOMO energy, the HOMO becomes more localised on the donor moiety and thus reduces the absolute spatial overlap of HOMO and LUMO, producing a lower oscillator strength for the S_0_–S_1_ transition.

**Figure 3:**
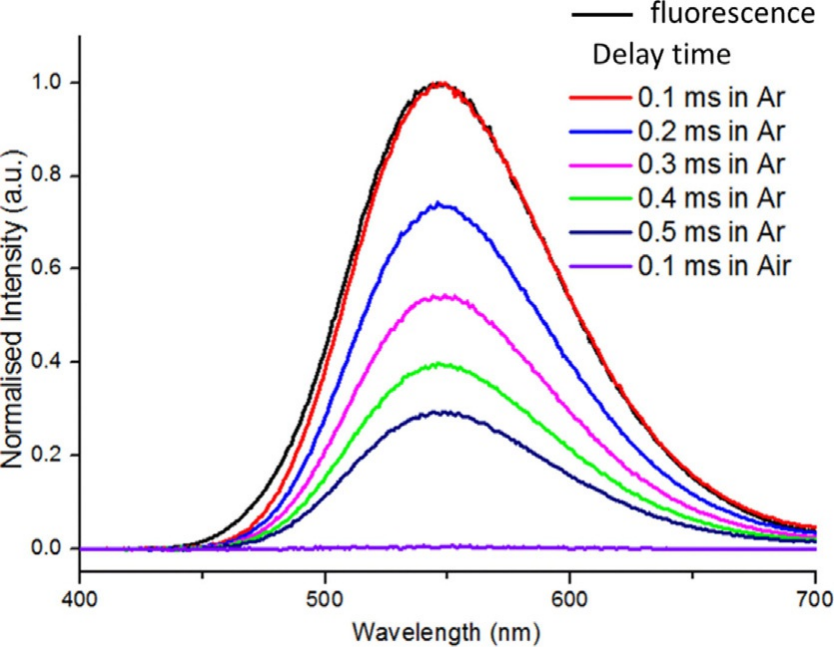
Emission of **3** in argon saturated toluene solution after various delay times and in air saturated toluene solution after 0.1 ms delay post excitation.

The optoelectronic properties of compounds **3**–**8** were measured in solution (Table 1). All six compounds showed reversible first reduction and oxidation waves, with the first reduction potential similar throughout. This is consistent with the LUMO being dominated by the acceptor unit in each case and agrees with/is supported by DFT calculations. The HOMO energy was dependent on the donor unit, and the values observed by CV were again in line with the DFT calculations with the phenoxazine derivatives being the most easily oxidised as expected. Analysis of the photophysical properties revealed that the phenoxazine compounds, **5** and **8**, were effectively non‐emissive; while the triphenylamine and carbazole congeners are both emissive with the carbazole congeners having significantly greater PLQY values. As expected the fluorene derivatives were more emissive in each case. The trend in PLQY mirrors that of the HOMO–LUMO absolute spatial overlap implicating these orbitals as the major contributors to the S_1_→S_0_ transitions, consistent with TD‐DFT and the calculated oscillator strengths (see subsequent discussion). More notable is the presence in argon saturated toluene solutions of long lifetime emission for both **3** and **6** (see Figure 3 and S20) which displayed delayed fluorescence (with emission maxima identical to the prompt fluorescence) when measured even after a 0.5 ms delay after excitation; this delayed emission is absent in air saturated solutions. In contrast, the carbazole derivatives **4** and **7** displayed no long lifetime emission under identical conditions. Furthermore, the fluorescence emission is notably increased for **3** and **6** when measured in argon saturated toluene solutions than when measured in air saturated solutions. These observations are consistent with TADF for **3** and **6** and are comparable to that observed in other emitters exhibiting TADF (see Figures S27 and S28).7a

**Table 1:** Optoelectronic properties of compounds **3–11**.

Compound	*E*_ox_ [V]^[a]^	*E*_red_ [V]^[a]^	*E*_gap_ [eV]	*λ*_max abs_ [nm]^[b]^	*ϵ*×10^3^ [m ^−1^ cm^−1^]	*λ*_max em_ [nm]^[b]^	Δ*E*_opt_ [eV]^[b]^	PLQY [%]^[c]^
3	0.42	−1.95	2.37	311, 319, 379	35.8, 35.6, 9.5	493	2.72	9.9
4	0.81	−1.93	2.74	343, 389	9.2, 9.4	492^e^	2.81	58.2
5	0.21	−1.93	2.14	320, 384	19.7, 10.4	–	2.80	–
6	0.42	−1.95	2.37	310, 403	73.4, 16.4	564	2.57	42.0
7	0.81	−1.94	2.75	412	17.9	516	2.69	75.7
8	0.22	−1.93	2.15	312, 408	47.0, 15.8	–	2.68	–
9	0.42	−2.03	2.45	318, 368, 400	55.2, 6.8, 3.5	544	2.67	3.9
10	0.42	−2.04	2.46	306, 379, 420	95.0, 12.7, 7.8	554	2.56	7.9
11	0.37	−1.88	2.25	348, 432	82.4, 47.3	542	2.50	69.0

[a] Measured in DCM (1 mm) with [*n*Bu_4_N][PF_6_] (0.1 m) as the supporting electrolyte at a scan rate of 50 mV s^−1^. Potentials are onset potentials given relative to the Fc/Fc^+^ redox couple which is taken to be 5.1 eV below vacuum. [b] Measured at 1×10^−5^ m in toluene, optical band gap from the onset of absorption. [c] absolute quantum yield values measured in air saturated toluene solutions using an integrating sphere (estimated error ±10 %).

**Figure 4:**
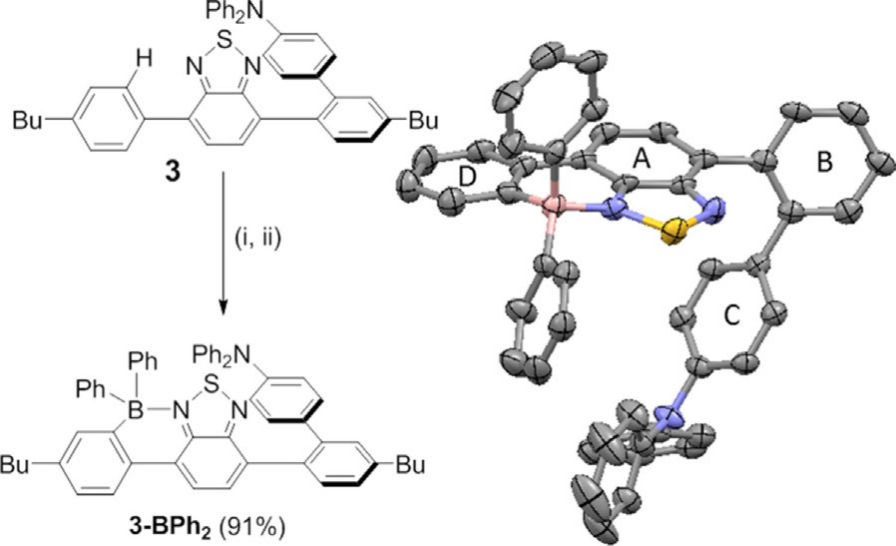
Left: the formation of **3‐BPh_2_**; (i) BCl_3_, CH_2_Cl_2_, 6 h, dried in vacuo; (ii) 2.2 equiv ZnPh_2_, CH_2_Cl_2_, 3 h. Right: the solid‐state structure of **3‐BPh_2_**, ellipsoids at 50 % probability, hydrogen atoms and *n*‐butyl groups omitted for clarity.

An analogue, the borylated compound **3‐BPh_2_** (Figure 4), with potentially a significant redshifted emission was investigated computationally (PBE0/6‐31G(d,p)/PCM (toluene)) using a model compound in which the butyl group has been replaced with hydrogen (termed **3_H_‐BPh_2_**). These calculations revealed the expected significant decrease in the LUMO energy compared to **3_H_** (**3_H_**=−2.28 eV; **3_H_‐BPh_2_**=−2.99 eV) with only a minor difference in the HOMO energies (**3_H_**=−5.23 eV; **3_H_‐BPh_2_**=−5.30 eV) from borylation as reported for non‐twisted compounds.8b The HOMO and LUMO of **3_H_‐BPh_2_** were calculated to have some character on ring B (Figure 4) and be closely comparable in character to the HOMO and LUMO calculated for **3_H_**. The non‐zero contribution to the HOMO and LUMO on ring B in **3_H_‐BPh_2_** is consistent with the absolute spatial HOMO–LUMO overlap value of 13.8 % and calculations which predict an emission (S_1_→S_0_) oscillator strength of *f*=0.0148 for this HOMO–LUMO dominated transition.

Compound **3‐BPh_2_** was synthesized in excellent yield from **3** (91 %) via directed electrophilic borylation with the formulation supported by multinuclear NMR spectroscopy, mass spectrometry and an X‐ray diffraction study (Figure 4, right). The calculated and solid state structures of **3_H_‐BPh_2_** and **3‐BPh_2_** were closely comparable, with that latter revealing a boracycle unit that has metrics closely comparable to previously reported examples.8b The mean plane of rings D and A were effectively co‐planar (angles between the mean planes of these rings=11.0°), but there was still a high degree of twist between rings A and B (angle between the mean planes of rings A and B=67.7° and between rings B and C=54.5°). The optical properties of **3‐BPh_2_** in toluene revealed the desired red‐shift in emission (relative to **3**), with emission maxima observed at 666 and 704 nm compared to 493 nm for **3**. However, the PLQY for **3‐BPh_2_** in toluene solution was extremely low (<1 %) in contrast to that for **3**. This is attributed to an increased non‐radiative rate constant from introducing B−Ph groups which can act as rotors to enable a non‐radiative relaxation mechanism. Consistent with this **3‐BPh_2_** is more emissive in the crystalline state, emitting in the red/near infra‐red region (emission maxima of 652 nm) of the spectrum with a solid state PLQY of 13.5 %. This is consistent with aggregation induced emission (AIE) preventing non‐radiative relaxation pathways,^12^ presumably in this case involving B−C_Ph_ rotation. Inspection of the extended packing structure for **3‐BPh_2_** reveals a dense extended structure with multiple close contacts between the BPh_2_ units and adjacent molecules, these interactions presumably contribute to blocking the rotational modes that in solution led to non‐radiative decay. It is also notable that in the crystalline state **3‐BPh_2_** exhibits delayed fluorescence, with an identical spectrum to the prompt fluorescence observed after a delay between excitation and measuring of 1 μs. While emitters exhibiting AIE and TADF are known,^13^ **3‐BPh_2_** is a rare example of a competent (solid‐state PLQY >10 %) emitter with emission maxima >650 nm and significant emission at *λ*>700 nm, that shows delayed fluorescence.^14^ It should be noted that compounds **3**–**8** are emissive in the solid state and solution, thus do not show the dramatic disparity between the solution and crystalline phases observed for **3‐BPh_2_**.

**Figure 5:**
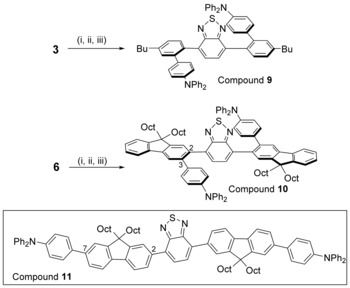
The synthesis of **9** and **10**; (i) in CH_2_Cl_2_+BCl_3_ (1 m in CH_2_Cl_2_, 2–3 equiv), 6 h; (ii) dried, +THF/H_2_O 10:1, stirred overnight; (iii) 4‐Bromotriphenylamine (1.1 equiv) solution degassed, then +Pd(*t*Bu_3_P)_2_, THF and K_3_PO_4_ 2 m (aq.), 18 h room temperature. Inset: the 2,7 fluorene‐isomer, **11**.

The formation of **3‐BPh_2_** demonstrates that a second directed C−H electrophilic borylation is feasible, therefore we sought to use this approach to install an additional donor group to provide access to twisted D‐A‐D compounds as a number of TADF emitters have twisted D‐A‐D structures (e.g. compound **B**).7a Sequential electrophilic borylation/Suzuki–Miyaura cross‐coupling of **3** and **6** (chosen as these two D−A compounds showed long lifetime emission) was performed to install a second Ph−NPh_2_ donor unit. Compounds **9** and **10** were accessed in good isolated yields (77 and 76 %, respectively, starting from **3** and **6**). To determine the importance of a 2,3 disubstituted fluorene unit (e.g. **10**, Figure 5), which leads to a large degree of twist between the donor and acceptor moieties, a 2,7‐disubstituted fluorene analogue, compound **11**, was synthesised via standard cross‐coupling methodologies (inset Figure 5).

D‐A‐D compounds **9** and **10** have comparable frontier orbital energies (from cyclic voltammetry) to their respective D−A compounds **3** and **6**; however, they were both significantly less emissive than **3** and **6** (Table 1). From TD‐DFT calculations (on a model of **9** where butyl has been replaced with hydrogen, termed **9_H_**) the S_0_–S_1_ transition is again dominated by the HOMO and LUMO (Figure S33) and for the D‐A‐D compound the oscillator strength is notably lower than the D−A analogue, consistent with the LUMO being more localised on the BT unit in **9** with less contribution from the flanking aryl rings (relative to that in **3**). While less emissive, both **9** and **10** still display delayed fluorescence in argon saturated toluene solutions with identical fluorescence spectra observed after a delay post excitation of 1.0 ms for **9** and **10**. The 2,7‐isomer, compound **11**, while having a higher PLQY for prompt emission than **10**, displayed no delayed fluorescence, indicating that in this case the highly twisted structure accessed by directed C−H borylation/cross‐coupling is essential for delayed fluorescence. For **11**, TD‐DFT calculations revealed that the S_0_–S_1_ transition is also dominated by the HOMO and LUMO, but inspection of these orbitals (Figure S35) revealed that the HOMO is more delocalised than observed in the twisted congener **10**. Thus **11** has more absolute spatial HOMO–LUMO overlap, which is consistent with the considerably larger oscillator strength (for **11** *f*=1.1306). The greater absolute spatial overlap of frontier orbitals in **11** (28.9 % compared to 18.0 % in **10**) also indicates that Δ*E*_S−T_ would be notably higher in **11** due to a greater exchange component.

**Figure 6:**
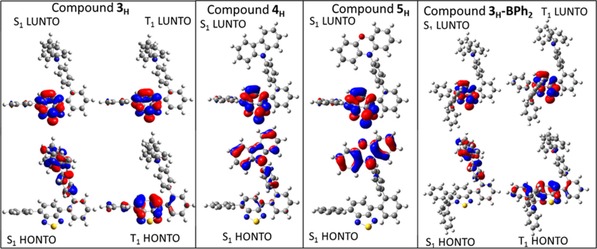
HONTO and LUNTO (isosurface value=0.04) for the S_0_–S_1_ and S_0_–T_1_ transitions for **3_H_**–**5_H_** and **3_H_‐BPh_2_** (the T_1_ HONTO and LUNTO for **4_H_** and **5_H_** are effectively identical to that for **3_H_**.

To provide more insight into the differences observed in the emission properties of **3** versus **4** and **5** more extensive calculations were performed on **3_H_**–**5_H_**. These were performed at the PBE0/6‐31G(d,p)/PCM(toluene) level and probed the nature of the excited states by calculating the frontier natural transition orbitals (NTOs) which provides more detail into the electron transitions of excited states.^15^, ^16^, ^17^ This indicated that S_1_ is predominantly an intramolecular charge transfer state (ICT) involving the HOMO and LUMO in all three cases but T_1_ is predominantly a local excited state on the benzothiadiazole acceptor unit (Figure 6 and Table 2). The T_2_ state is predominantly ICT in character in all three compounds, with each T_2_ state being similar in energy to the respective S_1_ state as expected for ^1^ICT and ^3^ICT states with low absolute overlap values of the hole and electron wavefunctions (thus a low magnitude of exchange). This series of BT containing compounds therefore involves an uncommon situation for organic D−A compounds where the lowest energy triplet state is a local excited state on the acceptor and not on the donor.7a Therefore the key to controlling Δ*E*_S−T_ (and also Δ*E*_T1−T2_)^18^ in these systems is matching the energy of the ICT S_1_ (≈T_2_) excited state to that of the local T_1_ excited state on the BT moiety. As the HOMO of carbazole based **4_H_** is lower in energy (than the HOMO in **3_H_**) this leads to a larger HOMO–LUMO energy gap, and as this gap correlates to the energy of the ICT states this results in Δ*E*_S−T_ (and Δ*E*_T1−T2_) being higher in energy for **4_H_** relative to **3_H_** (as the energy of the BT acceptor unit localised T_1_ excited state is effectively identical in both cases). Conversely as the HOMO of **5_H_** is significantly higher in energy (than in **3_H_**) and more localized on the NAr_2_ unit, Δ*E*_S−T_ (and Δ*E*_T1−T2_) is lower for this compound as S_1_ (and T_2_) are relatively low in energy and almost entirely ICT in character. However, the extremely low HOMO–LUMO absolute spatial overlap for **5_H_** leads to a very low oscillator strength for the S_1_→S_0_ transition and hence **5** is effectively non‐emissive. Therefore in line with previous studies, to observe significant delayed emission in twisted D−A compounds a balance has to be found that reduces Δ*E* between key excited states but maintains sufficient oscillator strength for the S_1_→S_0_ transition (and thus acceptable PLQY values).^1^ It should be noted that the other factors (in addition to Δ*E*_S−T_) that affect inter system crossing rates between the S_1_ and T_1_ states [see Eq. (1)], specifically the reorganisation energy (*λ*) and the spin orbit contribution (SOC), were also calculated (see Table S4). The values calculated for *λ* and the SOC for **3_H_–5_H_** showed only minor changes relative to the variation in Δ*E*_S−T_, therefore it is Δ*E*_S−T_ that dominates the magnitude of the rates of (forward and reverse) intersystem crossing, ISC, between T_1_ and S_1_. The larger Δ*E*_S−T_ in **4_H_** corresponds to small forward and reverse ISC rates, which are much lower than the rate of fluorescence (*k*_r_), thus no delayed emission is observed. While **5_H_** has larger rate constants for (R)ISC it is non‐emissive. Therefore, in these systems it is Δ*E* between the key excited states and the oscillator strength that dominate the observed variance in the photophysical properties of **3**–**5**. Both are modified by altering the donor (which alters the S_1_ HONTO character and energy), and in the case of **3** Δ*E*_S−T_ is sufficiently small and the oscillator strength sufficiently high for delayed fluorescence to be observed.

**Table 2:** Key parameters determined at the PBE0/6‐31G(d,p)/PCM(toluene) level for **3_H_**–**5_H_** and **3_H_‐BPh_2_**.

	HOMO/LUMO	*f*	S_1_				T_1_		T_2_		
	[eV]	S_1_→S_0_	S_h+e‐_^[a]^	% ICT/LE	Δ*E*_S0‐S1_ [eV]	S_h+e‐_^[a]^	% ICT/LE	Δ*E*_S0‐T1_ [eV]	S_h+e_^[a]^	[%] ICT/LE	Δ*E*_S0‐T2_ [eV]
**3_H_**	−5.23/−2.28	0.0278	20.7	92/8	2.44	82.2	14/86	1.85	29.5	89/11	2.48
**4_H_**	−5.65/−2.35	0.1062	31.5	85/15	2.82	83.4	10/90	1.87	24.8	90/10	2.85
**5_H_**	−4.97/−2.36	0.0003	4.3	95/5	2.18	83.5	10/90	1.88	8.9	95/5	2.18
**3_H_‐BPh_2_**	−5.30/−2.99	0.0148	19.6	93/7	1.81	77.2	16/84	1.39	25.5	90/10	1.87

[a] S_h+e‐_=% absolute overlap values of the hole and electron wavefunctions from the NTO calculations. ICT=intramolecular charge transfer state, LE=local excited state.

Finally, a model of the borylated compound, termed **3_H_‐BPh_2_**, was explored in more depth computationally. Borylative cyclisation has been previously shown to principally lower the energy of the LUMO and as the LUNTO of both S_1_, T_1_ and T_2_ excited states is dominated by the LUMO for **3_H_**–**5_H,_** it was expected that **3‐BPh_2_** would show delayed fluorescence provided *k*_r_ was not ≪*k*_nr_ (as is the case in solution). The calculations (Table 2) on **3_H_‐BPh_2_** showed comparable NTOs of the S_1_ (^1^ICT), T_1_ (^3^LE on the BT acceptor unit) and T_2_ (^3^ICT) states to those found for **3_H_**, but the S_1_, T_1_ and T_2_ states are all lower in energy post borylation. Furthermore, Δ*E*_S−T_ (and Δ*E*_T1−T2_) is smaller and thus higher (reverse) ISC rates for **3_H_‐Ph_2_** relative to **3_H_** are found. This is consistent with the long lifetime emission observed in the crystalline state for **3‐BPh_2_**_._

## Conclusions

In conclusion, a facile method to functionalise BT containing conjugated systems is presented that enables the generation of highly twisted D−A and D‐A‐D compounds. Photophysical studies revealed donor‐dependent long‐lifetime emission, which required structures with high degrees of twist, with a less twisted “linear” analogue (**11**) displaying no delayed fluorescence. Borylative cyclisation of a twisted D−A compound led to the expected red‐shift in emission while maintaining delayed emission (albeit only in the crystalline form due to aggregation induced emission). Detailed calculations revealed that the lowest energy triplet state is a local excited state located on the acceptor moiety and not the donor, with the T_2_ state being predominantly an intramolecular charge transfer state which is therefore comparable in energy to the S_1_ state. The significant variation in the long lifetime emission was determined to be due to: (i) a requirement to match the energy of the intramolecular charge transfer dominated excited states (S_1_/T_2_) with the local (on benzothiadiazole) T_1_ excited state, and (ii) to have sufficient S_0_–S_1_ oscillator strength. Both factors are highly sensitive to the character and energy of the donor unit's frontier orbitals, which if too low in energy leads to high energy ICT excited states (and thus a higher Δ*E* between the key excited states that reduces the rate of (reverse) intersystem crossing). Equally if the occupied frontier orbitals lie too high in energy this results in low S_0_–S_1_ oscillator strength due to effectively zero absolute spatial overlap of the HOMO and LUMO orbitals which dominate the S_1_–S_0_ transition.

## Experimental Section

For general considerations, NMR spectra, crystallographic information, further details on the optoelectronic properties (including spectra and CV plots) and computational information please see the Supporting Information.

Compound **3**: **1** (250 mg, 0.62 mmol) was dissolved in anhydrous DCM (3 mL) and BCl_3_ (1 m in DCM) (1.25 mL, 1.25 mmol) was added to the solution where a colour change from yellow to dark purple was observed. The solution was then stirred at ambient temperature for 3 hours under the dynamic flow of nitrogen. The solvent and other volatiles were then removed under reduced pressure and the resulting purple residue was dissolved in non‐anhydrous THF (10 mL). H_2_O (1 mL) was then added to the reaction mixture which was stirred overnight at ambient temperature were a colour change from purple to orange was observed. 4‐Bromotriphenylamine (222 mg, 0.69 mmol) was added to the reaction mixture which was then degassed (bubble N_2_). A solution of Pd(*t*Bu_3_P)_2_ (32 mg, 0.063 mmol) in THF (5 mL) was added to the degassed reaction mixture followed by the addition of K_3_PO_4_ 2 m (aq.) (1.56 mL, 3.12 mmol). The reaction mixture was then stirred overnight at ambient temperature. The reaction mixture was diluted with ethyl acetate (50 mL) followed by the addition of brine (10 mL) and deionised water (30 mL). The organic layer was isolated using a separating funnel and dried (MgSO_4_). The solvent was evaporated under reduced pressure and the resulting residue was purified using silica gel chromatography [eluent=2:8 CHCl_3_: petroleum ether]. The desired product was then isolated as a yellow solid. Yield: 342 mg, 85 %. Reaction repeated using **1** (1.90 g, 4.74 mmol), BCl_3_ (1 m in DCM) (10 mL, 10 mmol), 4‐Bromotriphenylamine (1.61 g, 4.98 mmol), K_3_PO_4_ 2 m (aq.) (11.85 mL, 23.70 mmol) and Pd(PPh_3_)_4_ (274 mg, 0.37 mmol) (heating overnight at 75 °C). Yield: 1.98 g, 74 %. HR‐MS (APCI mode: positive): *m*/*z* calcd for C_44_H_42_N_3_S^+^ [*M*+H]^+^ 644.3094, found 644.3092. ^1^H NMR: (400 MHz, CDCl_3_): *δ*=7.86–7.74 (m, 2 H), 7.54 (d, *J=*7.1 Hz, 1 H), 7.50 (d, *J=*7.8 Hz, 1 H), 7.40–7.33 (m, 2 H), 7.33–7.27 (m, *J=*8.3 Hz, 2 H), 7.25 (dd, *J=*1.7, 7.8 Hz, 1 H), 7.14–7.06 (m, 4 H), 6.97–6.91 (m, 2 H), 6.91–6.82 (m, 6 H), 6.75–6.67 (m, 2 H), 2.77–2.57 (m, 4 H), 1.76–1.51 (m, 4 H), 1.46–1.30 (m, 4 H), 0.93 (t, *J=*7.3 Hz, 3 H), 0.91 ppm (t, *J=*7.3 Hz, 3 H); ^13^C{^1^H} NMR: (101 MHz, CDCl_3_): *δ*=155.3, 154.1, 148.2, 146.7, 144.1, 143.9, 141.9, 136.9, 135.4, 134.5, 134.4, 133.3, 131.6, 131.1, 130.7, 130.6, 129.7, 129.1, 127.9, 127.7, 124.5, 123.9, 123.2, 36.0, 36.0, 34.3, 23.1, 23.0, 14.4, 14.3 ppm.

Compound **4**: **1** (279 mg, 0.70 mmol) was dissolved in anhydrous DCM (3 mL) and BCl_3_ (1 m in DCM) (1 mL, 1 mmol) was added to the solution where a colour change from yellow to dark purple was observed. The solution was then stirred at ambient temperature for 0.5 hours under the dynamic flow of nitrogen. The solvent and other volatiles were then removed under reduced pressure and the resulting purple residue was dissolved in non‐anhydrous THF (10 mL). H_2_O (2 mL) was then added to the reaction mixture which was stirred overnight at ambient temperature were a colour change from purple to orange was observed. 9‐(4‐Bromophenyl)‐9*H*‐carbazole (235 mg, 0.75 mmol) was added to the reaction mixture which was then degassed (bubble N_2_). Pd(PPh_3_)_4_ (40 mg, 0.035 mmol) was added to the degassed reaction mixture followed by the addition of K_3_PO_4_ 2 m (aq.) (1.75 mL, 3.50 mmol). The reaction mixture was then stirred for 10 hours at 75 °C. The reaction mixture was diluted with ethyl acetate (50 mL) followed by the addition of brine (10 mL) and deionised water (30 mL). The organic layer was isolated using a separating funnel and dried (MgSO_4_). The solvent was evaporated under reduced pressure and the resulting residue was purified using silica gel chromatography [eluent=1:9 DCM: petroleum ether graduated to 2:8 DCM:petroleum ether]. The desired product was then isolated as a yellow/green solid. Yield: 375 mg, 84 %. HR‐MS (APCI mode: positive): *m*/*z* calcd for C_44_H_40_N_3_S^+^ [*M*+H]^+^ 642.2937, found 642.2938. ^1^H NMR: (400 MHz, CDCl_3_): *δ*=8.17 (d, *J=*7.6 Hz, 2 H), 7.95 (d, *J=*8.3 Hz, 2 H), 7.71 (d, *J=*7.3 Hz, 2 H), 7.59 (d, *J=*1.5 Hz, 1 H), 7.53 (d, *J=*7.1 Hz, 1 H), 7.50–7.39 (m, 7 H), 7.37–7.25 (m, 6 H), 2.95–2.83 (m, 2 H), 2.77 (t, *J=*7.7 Hz, 2 H), 1.95–1.80 (m, 2 H), 1.80–1.67 (m, 2 H), 1.63–1.43 (m, 4 H), 1.09 (t, *J=*7.3 Hz, 3 H), 1.04 ppm (t, *J=*7.3 Hz, 3 H); ^13^C{^1^H} NMR: (101 MHz, CDCl_3_): *δ*=154.7, 153.5, 143.6, 143.2, 141.0, 140.6, 135.8, 134.5, 133.7, 133.3, 133.0, 131.1, 130.7, 130.6, 130.4, 129.0, 128.7, 127.8, 127.3, 126.2, 125.8, 123.2, 120.2, 119.8, 109.5, 35.5, 35.4, 33.5, 22.5, 22.3, 14.0, 13.9 ppm.

Compound **6**: **1** (323 mg, 0.81 mmol) was dissolved in anhydrous DCM (3 mL) and BCl_3_ (1 m in DCM) (1.2 mL, 1.2 mmol) was added to the solution where a colour change from yellow to dark purple was observed. The solution was then stirred at ambient temperature for 0.5 hours under the dynamic flow of nitrogen. The solvent and other volatiles were then removed under reduced pressure and the resulting purple residue was dissolved in non‐anhydrous THF (30 mL). H_2_O (3 mL) was then added to the reaction mixture which was stirred overnight at ambient temperature were a colour change from purple to orange was observed. 10‐(4‐bromophenyl)‐10*H*‐phenoxazine (286 mg, 0.85 mmol) was added to the reaction mixture which was then degassed (bubble N_2_). Pd(PPh_3_)_4_ (46 mg, 0.04 mmol) was added to the degassed reaction mixture followed by the addition of K_3_PO_4_ 2 m (aq.) (2.00 mL, 4.00 mmol). The reaction mixture was then stirred for 12 hours at 75 °C. The reaction mixture was diluted with ethyl acetate (50 mL) followed by the addition of brine (10 mL) and deionised water (30 mL). The organic layer was isolated using a separating funnel and dried (MgSO_4_). The solvent was evaporated under reduced pressure and the resulting residue was purified using silica gel chromatography [eluent=2:8 DCM: petroleum]. The desired product was then isolated as a yellow solid. Yield: 468 mg, 88 %. HR‐MS (APCI mode: positive): *m*/*z* calcd for C_44_H_40_ON_3_S^+^ [*M*+H]^+^ 658.2887, found 658.2893. ^1^H NMR: (400 MHz, CDCl_3_): *δ*=7.92–7.79 (m, *J=*8.1 Hz, 2 H), 7.63 (d, *J=*7.8 Hz, 1 H), 7.66 (d, *J=*7.3 Hz, 1 H), 7.56–7.46 (m, 2 H), 7.41 (dd, *J=*1.5, 7.8 Hz, 1 H), 7.39–7.31 (m, 4 H), 7.09–6.97 (m, *J=*8.5 Hz, 2 H), 6.70–6.49 (m, 6 H), 5.61 (dd, *J=*1.5, 7.8 Hz, 2 H), 2.87–2.77 (m, 2 H), 2.71 (t, *J=*7.7 Hz, 2 H), 1.85–1.73 (m, 2 H), 1.73–1.63 (m, 2 H), 1.56–1.47 (m, 2 H), 1.47–1.35 (m, 2 H), 1.03 (t, *J=*7.3 Hz, 3 H), 0.98 ppm (t, *J=*7.3 Hz, 3 H); ^13^C{^1^H} NMR: (101 MHz, CDCl_3_): *δ*=154.5, 153.5, 143.8, 143.6, 143.3, 142.3, 140.7, 137.0, 134.5, 134.2, 133.8, 133.4, 133.2, 131.8, 131.0, 130.6, 130.2, 129.9, 129.0, 128.7, 128.0, 127.2, 123.1, 121.1, 115.3, 113.0, 35.5, 35.4, 33.6, 33.5, 22.6, 22.4, 14.0, 14.0 ppm.

Compound **6**: **2** (294 g, 0.32 mmol) was dissolved in anhydrous DCM (5 mL) and BCl_3_ (1 m in DCM) (0.8 mL, 0.8 mmol) was added to the solution where a colour change from yellow to dark purple was observed. The solution was then stirred at ambient temperature for 3 hours under the dynamic flow of nitrogen. The solvent and other volatiles were then removed under reduced pressure and the resulting purple residue was dissolved in non‐anhydrous THF (10 mL). H_2_O (1 mL) was then added to the reaction mixture which was stirred overnight at ambient temperature were a colour change from purple to orange was observed. 4‐Bromotriphenylamine (115 mg, 0.35 mmol) was added to the reaction mixture which was then degassed (bubble N_2_). A solution of Pd(*t*Bu_3_P)_2_ (17 mg, 0.033 mmol) in THF (3 mL) was added to the degassed reaction mixture followed by the addition of K_3_PO_4_ 2 m (aq.) (0.80 mL, 1.60 mmol). The reaction mixture was then stirred overnight at ambient temperature. The reaction mixture was diluted with ethyl acetate (50 mL) followed by the addition of brine (10 mL) and deionised water (30 mL). The organic layer was isolated using a separating funnel and dried (MgSO_4_). The solvent was evaporated under reduced pressure and the resulting residue was purified using silica gel chromatography [eluent=1:9 DCM: petroleum ether]. The desired product was then isolated as a yellow solid. Yield: 320 mg, 86 %. Reaction repeated using **2** (1.00 g, 1.09 mmol), BCl_3_ (1 m in DCM) (3 mL, 3 mmol), 4‐bromotriphenylamine (373 mg, 1.14 mmol), K_3_PO_4_ 2 m (aq.) (2.8 mL, 5.6 mmol) and Pd(PPh_3_)_4_ (63 mg, 0.05 mmol) (heating overnight at 75 °C). Yield: 873 mg, 74 %. HR‐MS (APCI mode: positive): *m*/*z* calcd for C_82_H_98_N_3_S^+^ [*M*+H]^+^ 1156.7476, found 1156.7490. ^1^H NMR: (400 MHz, CDCl_3_): *δ*=7.99 (dd, *J=*1.5, 7.8 Hz, 1 H), 7.95–7.91 (m, 1 H), 7.90 (s, 1 H), 7.85 (d, *J=*8.0 Hz, 1 H), 7.80–7.69 (m, 3 H), 7.60 (s, 1 H), 7.56 (d, *J=*7.3 Hz, 1 H), 7.43–7.25 (m, 6 H), 7.20–7.10 (m, 6 H), 6.99–6.87 (m, 6 H), 6.86–6.78 (m, 2 H), 2.17–1.87 (m, 8 H), 1.26–1.01 (m, 40 H), 0.96–0.66 ppm (m, 20 H); ^13^C{^1^H} NMR: (101 MHz, CDCl_3_): *δ*=154.8, 153.7, 151.5, 151.2, 151.0, 149.4, 147.6, 146.1, 141.5, 141.3, 140.7, 140.6, 140.1, 136.6, 136.1, 135.1, 134.8, 133.3, 130.5, 130.3, 129.1, 128.2, 127.3, 127.3, 127.2, 126.9, 126.8, 126.0, 123.9, 123.7, 123.5, 123.0, 122.9, 122.6, 121.3, 119.9, 119.7, 55.2, 55.1, 40.3, 40.1, 31.8, 30.1, 29.2, 29.2, 24.0, 23.9, 22.6, 22.6, 14.1, 14.1 ppm.

Compound **7**: **2** (145 g, 0.16 mmol) was dissolved in anhydrous DCM (5 mL) and BCl_3_ (1 m in DCM) (0.3 mL, 0.3 mmol) was added to the solution where a colour change from yellow to dark purple was observed. The solution was then stirred at ambient temperature for 3 hours under the dynamic flow of nitrogen. The solvent and other volatiles were then removed under reduced pressure and the resulting purple residue was dissolved in non‐anhydrous THF (10 mL). H_2_O (1 mL) was then added to the reaction mixture which was stirred overnight at ambient temperature were a colour change from purple to orange was observed. 9‐(4‐Bromophenyl)‐9*H*‐carbazole (56 mg, 0.18 mmol) was added to the reaction mixture which was then degassed (bubble N_2_). A solution of Pd(*t*Bu_3_P)_2_ (4 mg, 0.008 mmol) in THF (3 mL) was added to the degassed reaction mixture followed by the addition of K_3_PO_4_ 2 m (aq.) (0.40 mL, 0.80 mmol). The reaction mixture was then stirred overnight at ambient temperature. The reaction mixture was diluted with ethyl acetate (50 mL) followed by the addition of brine (10 mL) and deionised water (30 mL). The organic layer was isolated using a separating funnel and dried (MgSO_4_). The solvent was evaporated under reduced pressure and the resulting residue was purified using silica gel chromatography [eluent=1:9 DCM: petroleum ether]. The desired product was then isolated as a yellow solid. Yield: 136 mg, 74 %. HR‐MS (APCI mode: positive): *m*/*z* calcd for C_82_H_96_N_3_S^+^ [*M*+H]^+^ 1154.7319, found 1154.7340. ^1^H NMR: (400 MHz, CD_2_Cl_2_=CH_2_Cl_2_): *δ*=8.15 (d, *J*=7.3 Hz, 2 H), 8.07 (d, *J*=8.5 Hz, 3 H), 7.94–7.88 (m, 2 H), 7.86 (d, *J*=7.3 Hz, 1 H), 7.84–7.79 (m, 1 H), 7.77 (s, 1 H), 7.68 (d, *J*=7.1 Hz, 1 H), 7.55 (d, *J*=8.3 Hz, 2 H), 7.52–7.33 (m, 10 H), 7.33–7.24 (m, 4 H), 2.24–1.06 (m, 40 H), 1.01–0.72 ppm (m, 20 H); ^13^C{^1^H} NMR: (101 MHz, CDCl_3_): *δ*=155.5, 154.3, 152.2, 151.9, 151.7, 150.7, 142.2, 142.1, 141.9, 141.3, 141.2, 141.0, 140.4, 136.8, 136.4, 136.0, 134.9, 134.1, 131.5, 131.4, 128.9, 128.1, 128.0, 127.9, 127.6, 127.4, 126.9, 126.9, 126.5, 124.5, 123.8, 123.8, 123.6, 122.0, 120.7, 120.6, 120.5, 120.4, 120.1, 110.2, 55.8, 40.8, 40.7, 32.4, 32.4, 30.7, 30.6, 29.9, 29.8, 29.8, 24.7, 24.5, 23.3, 23.2, 14.5, 14.4 ppm.

Compound **8**: **2** (186 g, 0.20 mmol) was dissolved in anhydrous DCM (5 mL) and BCl_3_ (1 m in DCM) (0.4 mL, 0.4 mmol) was added to the solution where a colour change from yellow to dark purple was observed. The solution was then stirred at ambient temperature for 3 hours under the dynamic flow of nitrogen. The solvent and other volatiles were then removed under reduced pressure and the resulting purple residue was dissolved in non‐anhydrous THF (10 mL). H_2_O (1 mL) was then added to the reaction mixture which was stirred overnight at ambient temperature were a colour change from purple to orange was observed. 10‐(4‐Bromophenyl)‐10*H*‐phenoxazine (72 mg, 0.21 mmol) was added to the reaction mixture which was then degassed (bubble N_2_). A solution of Pd(*t*Bu_3_P)_2_ (5 mg, 0.010 mmol) in THF (3 mL) was added to the degassed reaction mixture followed by the addition of K_3_PO_4_ 2 m (aq.) (0.50 mL, 1.00 mmol). The reaction mixture was then stirred overnight at ambient temperature. The reaction mixture was diluted with ethyl acetate (50 mL) followed by the addition of brine (10 mL) and deionised water (30 mL). The organic layer was isolated using a separating funnel and dried (MgSO_4_). The solvent was evaporated under reduced pressure and the resulting residue was purified using silica gel chromatography [eluent=15:85 DCM: petroleum ether]. The desired product was then isolated as a yellow solid. Yield: 158 mg, 66 %. HR‐MS (APCI mode: positive): *m*/*z* calcd for C_82_H_96_N_3_OS^+^ [*M*+H]^+^ 1170.7269, found 1170.7285. ^1^H NMR: (400 MHz, CD_2_Cl_2_): *δ*=8.10–7.96 (m, 3 H), 7.92–7.86 (m, 2 H), 7.86–7.78 (m, 2 H), 7.73 (s, 1 H), 7.69 (d, *J=*7.3 Hz, 1 H), 7.58–7.32 (m, 8 H), 7.09 (d, *J=*8.3 Hz, 2 H), 6.75–6.48 (m, 6 H), 5.64 (d, *J=*6.0 Hz, 2 H), 2.21–1.97 (m, 8 H), 1.37–1.03 (m, 40 H), 1.00–0.71 ppm (m, 20 H); ^13^C{^1^H} NMR: (101 MHz, CDCl_3_): *δ*=154.8, 153.6, 151.5, 151.4, 151.1, 150.3, 143.9 (br.), 142.8, 141.7, 141.4, 140.6, 140.5, 140.0, 137.0 (br.), 136.1, 135.6, 134.4, 134.3 (br.), 133.7, 132.2, 130.7, 130.0 (br.), 128.2, 127.6, 127.4, 127.4, 127.0, 126.9, 126.0, 124.0, 123.2, 123.1, 121.2, 120.1, 119.9, 119.6, 115.2 (br.), 113.1, 55.3, 55.3, 40.2, 40.2, 31.9, 31.8, 30.1, 30.0, 29.3, 24.1, 23.9, 22.7, 22.6, 13.9, 13.9 ppm.

Compound **3‐BPh_2_**: **3** (48 mg, 0.075 mmol) was dissolved in anhydrous DCM (3 mL) and BCl_3_ (1 m in DCM) (1 mL, 1 mmol) was added to the solution where a colour change from yellow to dark red was observed. The solution was then stirred at ambient temperature for 6 hours under the dynamic flow of nitrogen where upon the solution had become a dark purple colour. The solvent and other volatiles were then removed under reduced pressure and the resulting purple residue was dissolved in DCM (3 mL). ZnPh_2_ (36 mg, 0.164 mmol) was then added to the solution and the reaction mixture was stirred for 3 hours at ambient temperature. The reaction mixture was then passed through a plug of silica gel (eluent DCM) with only the red coloured fractions retained. The solvent was then removed under reduced pressure to give the desired product as a dark red solid. Yield: 55 mg, 91 %. HR‐MS (APCI mode: positive): *m*/*z* calc. for C_56_H_51_N_3_BS^+^ [*M*+H]^+^ 808.3891, found 808.3898.^1^H NMR: (400 MHz, CDCl_3_): *δ*=8.16 (d, *J=*7.8 Hz, 1 H), 8.00 (d, *J=*8.3 Hz, 1 H), 7.64 (d, *J=*7.5 Hz, 1 H), 7.55 (d, *J=*7.8 Hz, 1 H), 7.41 (dd, *J=*1.5, 20.0 Hz, 2 H), 7.35 (dd, *J=*1.8, 7.8 Hz, 1 H), 7.26–7.12 (m, 15 H), 7.07–6.97 (m, 8 H), 6.89–6.81 (m, 2 H), 2.84–2.72 (m, 2 H), 2.61 (t, *J=*7.7 Hz, 2 H), 1.82–1.70 (m, 2 H), 1.67–1.55 (m, 2 H), 1.55–1.43 (m, 3 H), 1.40–1.29 (m, 2 H), 1.02 (t, *J=*7.4 Hz, 3 H), 0.92 ppm (t, *J=*7.4 Hz, 3 H); ^13^C{^1^H} NMR: (101 MHz, CDCl_3_): *δ*=154.5 (br.), 154.0, 152.9 (br.), 147.6, 147.4, 146.6, 144.1, 143.5, 141.3, 135.1, 134.8, 133.6, 133.4, 132.9, 131.5, 131.0, 130.6, 129.9, 129.2, 128.6, 128.0, 127.5, 127.3, 126.5, 125.8, 124.5, 123.2, 123.0, 122.4, 122.1, 35.7, 35.5, 33.5, 33.4, 22.5, 22.3, 14.0, 13.9 ppm; ^11^B NMR: (128 MHz, CDCl_3_) *δ*=1.66 ppm.

Compound **9**: **3** (205 mg, 0.32 mmol) was dissolved in anhydrous DCM (3 mL) and BCl_3_ (1 m in DCM) (1 mL, 1 mmol) was added to the solution where a colour change from yellow to dark red was observed. The solution was then stirred at ambient temperature for 6 hours under the dynamic flow of nitrogen where upon the solution had become a dark purple colour. The solvent and other volatiles were then removed under reduced pressure and the resulting purple residue was dissolved in non‐anhydrous THF (10 mL). H_2_O (1 mL) was then added to the reaction mixture which was stirred overnight at ambient temperature were a colour change from purple to orange was observed. 4‐Bromotriphenylamine (114 mg, 0.35 mmol) was added to the reaction mixture which was then degassed (bubble N_2_). A solution of Pd(*t*Bu_3_P)_2_ (8 mg, 0.016 mmol) in THF (3 mL) was added to the degassed reaction mixture followed by the addition of K_3_PO_4_ 2 m (aq.) (0.90 mL, 1.80 mmol). The reaction mixture was then stirred overnight at ambient temperature. The reaction mixture was diluted with ethyl acetate (50 mL) followed by the addition of brine (10 mL) and deionised water (30 mL). The organic layer was isolated using a separating funnel and dried (MgSO_4_). The solvent was evaporated under reduced pressure and the resulting residue was purified using silica gel chromatography [eluent=2:8 CHCl_3_: petroleum ether graduated to 4:6 CHCl_3_: petroleum ether]. The desired product was then isolated as a yellow solid. Yield: 217 mg, 77 %. HR‐MS (APCI mode: positive): *m*/*z* calcd for C_62_H_55_N_4_S^+^ [*M*+H]^+^ 887.4142, found 887.4146. ^1^H NMR: (400 MHz, CD_2_Cl_2_): *δ*=7.52 (d, *J=*7.6 Hz, 1 H), 7.49–7.44 (m, 1 H), 7.40–7.32 (m, 2 H), 7.18 (t, *J=*7.9 Hz, 4 H), 6.96 (dd, *J=*5.9, 7.8 Hz, 4 H), 6.91 (d, *J=*7.8 Hz, 4 H), 6.65 (d, *J=*8.5 Hz, 2 H), 2.84 (t, *J=*7.7 Hz, 2 H), 1.82 (quin, *J=*7.6 Hz, 2 H), 1.55 (sxt, *J=*7.4 Hz, 2 H), 1.08 ppm (t, *J=*7.3 Hz, 3 H); ^13^C{^1^H} NMR: (101 MHz, CD_2_Cl_2_): *δ*=154.7, 148.2, 146.8, 144.0, 141.8, 136.7, 134.4, 134.4, 131.6, 130.7, 130.6, 130.6, 129.7, 127.6, 124.4, 124.0, 123.1, 36.1, 34.3, 23.2, 14.5 ppm.

Compound **10**: **6** (380 mg, 0.35 mmol) was dissolved in anhydrous DCM (5 mL) and BCl_3_ (1 m in DCM) (0.75 mL, 0.75 mmol) was added to the solution where a colour change from yellow to dark red was observed. The solution was then stirred at ambient temperature for 6 hours under the dynamic flow of nitrogen where upon the solution had become a dark purple colour. The solvent and other volatiles were then removed under reduced pressure and the resulting purple residue was dissolved in non‐anhydrous THF (10 mL). H_2_O (1 mL) was then added to the reaction mixture which was stirred overnight at ambient temperature were a colour change from purple to orange was observed. 4‐Bromotriphenylamine (120 mg, 0.37 mmol) was added to the reaction mixture which was then degassed (bubble N_2_). A solution of Pd(*t*Bu_3_P)_2_ (9 mg, 0.018 mmol) in THF (5 mL) was added to the degassed reaction mixture followed by the addition of K_3_PO_4_ 2 m (aq.) (1.56 mL, 3.12 mmol). The reaction mixture was then stirred overnight at ambient temperature. The reaction mixture was diluted with ethyl acetate (50 mL) followed by the addition of brine (10 mL) and deionised water (30 mL). The organic layer was isolated using a separating funnel and dried (MgSO_4_). The solvent was evaporated under reduced pressure and the resulting residue was purified using silica gel chromatography [eluent=1:9 DCM: pentane]. The desired product was then isolated as a yellow solid. Yield: 367 mg, 76 %. Reaction repeated using **6** (172 mg, 0.15 mmol), BCl_3_ (1 m in DCM) (0.3 mL, 0.3 mmol), 4‐Bromotriphenylamine (54 mg, 0.16 mmol), K_3_PO_4_ 2 m (aq.) (0.35 mL, 0.70 mmol) and Pd(PPh_3_)_4_ (9 mg, 0.008 mmol) (heating overnight at 75 °C). Yield: 128 mg, 62 %. HR‐MS (APCI mode: positive): *m*/*z* calcd for C_100_H_111_N_4_S^+^ [*M*+H]^+^ 1399.8524, found 1399.8546. ^1^H NMR: (400 MHz, CD_2_Cl_2_): *δ*=7.91 (s, 2 H), 7.87–7.78 (m, 2 H), 7.60 (s, 2 H), 7.49–7.34 (m, 8 H), 7.21–7.09 (m, 8 H), 7.00 (d, *J=*8.5 Hz, 4 H), 6.96–6.80 (m, 12 H), 6.65 (d, *J=*8.5 Hz, 4 H), 2.16–1.93 (m, 8 H), 1.27–1.08 (m, 40 H), 0.96–0.76 ppm (m, 20 H); ^13^C{^1^H} NMR: (101 MHz, CDCl_3_): *δ*=154.2, 151.5, 149.3, 147.6, 146.1, 141.3, 140.6, 140.1, 136.5, 135.1, 134.5, 130.2, 130.1, 129.1, 127.3, 126.9, 125.8, 123.8, 123.5, 123.0, 122.5, 121.2, 119.9, 55.0, 40.1, 31.8, 30.1, 29.2, 29.2, 24.0, 22.6, 14.1 ppm.

Compound **11**: 4,7‐bis(7‐bromo‐9,9‐dioctyl‐9*H*‐fluoren‐2‐yl)‐2,1,3‐benzothiadiazole (200 mg, 0.19 mmol) and (4‐(diphenylamino)phenyl)boronic acid was dissolved in degassed (bubble N_2_) THF (10 mL). A solution of Pd(*t*Bu_3_P)_2_ (10 mg, 0.02 mmol) in THF (3 mL) was added to the degassed reaction mixture followed by the addition of K_3_PO_4_ 2 m (aq.) (1.0 mL, 2.0 mmol). The reaction mixture was then stirred overnight at ambient temperature. The reaction mixture was diluted with ethyl acetate (50 mL) followed by the addition of brine (10 mL) and deionised water (30 mL). The organic layer was isolated using a separating funnel and dried (MgSO_4_). The solvent was evaporated under reduced pressure and the resulting residue was purified using silica gel chromatography [eluent=15:85 DCM: petroleum ether graduated to 30:70 DCM: petroleum ether]. The desired product was then isolated as an orange solid. Yield: 195 mg, 74 %. HR‐MS (APCI mode: positive): *m*/*z* calcd for C_100_H_111_ON_3_S^+^ [*M*+H]^+^ 1399.8524, found 1399.8533. ^1^H NMR: (400 MHz, CDCl_3_): *δ*=8.09 (dd, *J*=1.2, 8.1 Hz, 2 H), 8.03 (s, 2 H), 7.98–7.89 (m, 4 H), 7.85 (d, *J*=8.3 Hz, 2 H), 7.70–7.58 (m, 8 H), 7.37–7.29 (m, 8 H), 7.26–7.18 (m, 12 H), 7.12–7.05 (m, 4 H), 2.27–2.00 (m, 8 H), 1.29–1.11 (m, 40 H), 0.95–0.79 ppm (m, 20 H); ^13^C{^1^H} NMR: (101 MHz, CDCl_3_): *δ*=154.3, 152.0, 151.3, 147.7, 147.1, 141.1, 139.7, 139.5, 136.1, 135.6, 133.5, 129.3, 128.2, 127.9, 127.8, 125.6, 124.4, 124.0, 123.9, 122.9, 121.0, 120.2, 119.7, 55.3, 40.3, 31.8, 30.1, 29.2, 29.2, 23.9, 22.6, 14.1 ppm.

## Conflict of interest

The authors declare no conflict of interest.

## Supporting information

As a service to our authors and readers, this journal provides supporting information supplied by the authors. Such materials are peer reviewed and may be re‐organized for online delivery, but are not copy‐edited or typeset. Technical support issues arising from supporting information (other than missing files) should be addressed to the authors.

Supplementary
