# Supplementary material for: C−H Borylation/Cross‐Coupling Forms Twisted Donor–Acceptor Compounds Exhibiting Donor‐Dependent Delayed Emission
Source: Chemistry. 2018 Jun 25;24(41):10521–30. doi: 10.1002/chem.201801799 (PMC6099339; doi:10.1002/chem.201801799)
Supplement: Supplementary file 1 — Supplementary [file CHEM-24-10521-s001.pdf]

CHEMISTRY
A European Journal

Supporting Information

C−H Borylation/Cross-Coupling Forms Twisted Donor–Acceptor Compounds Exhibiting Donor-Dependent Delayed Emission

Daniel L. Crossley, Pakapol Kulapichitr, James E. Radcliffe, Jay J. Dunsford, Inigo Vitorica-Yrezabal, Rachel J. Kahan, Adam W. Woodward, Michael L. Turner,* Joseph J. W. McDouall,* and Michael J. Ingleson*[a]

chem_201801799_sm_miscellaneous_information.pdf

Table of Contents

General Considerations	S2
Synthetic Procedures	S5
NMR Spectra	S16
Optical Properties	S28
Electrochemical Properties	S32
Delayed Emission Studies	S37
Crystallography Details	S50
Computational Details	S53

General Considerations

Unless otherwise indicated all reagents were purchased from commercial sources and were used without further purification. 4,7-bis(9,9-dioctyl-9H-fluoren-2-yl)-2,1,3-Benzothiadiazole (compound 2)[S1], 4,7-bis(7-bromo-9,9-dioctyl-9H-fluoren-2-yl)-2,1,3-Benzothiadiazole[S1] and 10-(4-bromophenyl)-10H-phenoxazine were synthesised according to literature procedures[S2]. All appropriate manipulations were performed using standard Schlenk techniques or in an argon-filled MBraun glovebox (O_2 levels below 0.5 ppm). Glassware was dried in a hot oven overnight and heated under vacuum before use. Solvents were distilled from NaK, CaH_2, or K and degassed prior to use. Dichloromethane and THF were stored over activated 3 Å molecular sieves while toluene was stored over a potassium mirror.

NMR spectra were recorded using a Bruker AV-400 spectrometer (400 MHz ^{1}H, 101 MHz ^{13}C{^{1}H}, 128 MHz ^{11}B, 376 MHz ^{19}F{^{1}H}). The chemical shift values of the ^{1}H NMR and ^{13}C{^{1}H} NMR spectra are reported in ppm relative to residual protio solvent (e.g. $CHCl_3$ in $CDCl_3$ δH = 7.27 or δC = 77.2) as internal standards. The ^{19}F{^{1}H} NMR spectra were referenced to C_6F_6, ^{11}B NMR spectra were referenced to external BF_3:Et_2O. Unless otherwise stated all NMR spectra are recorded at 293 K. Broad features in the ^{11}B NMR spectra are due to boron present in borosilicate glass. Carbon atoms directly bonded to boron are not always observed in the ^{13}C{^{1}H} NMR spectra due to quadrupolar relaxation leading to signal broadening. Coupling constants *J* are given in Hertz (Hz) as positive values regardless of their real individual signs. The multiplicity of the signals are indicated as “s”, “d”, “t”, “q”, “quin”, “sxt” “sept” or “m” for singlet, doublet, triplet, quartet, quintet, sextet septet, or multiplet, respectively. (br.) denotes a broad signal.

Matrix assisted laser desorption/ionization time of flight (MALDI-TOF), atmospheric pressure chemical ionization (APCI) and Electrospray ionization (ESI) measurements was performed by the Mass Spectrometry Service, School of Chemistry, University of Manchester. MALDI-TOF analyses were performed using a Shimadzu Axima Confidence spectrometer using a 4k PPG as a calibration reference. 1 µL of a solution of dopant NaI in THF (10 mg mL^{-1}) was spotted onto a well of the MALDI plate and the solvent left to evaporate. Solutions were made up to 10 mg mL^{-1} in DCM. A solution of matrix dithranol was made up to 10 mg mL^{-1} in

THF. 2 μL of sample solution and 20 μL of matrix solution were thoroughly mixed and 1 μL of this solution was spotted onto a well with no dopant and 1 μL spotted by a layered method with the NaI. The solvent was allowed to evaporate before being placed in the spectrometer. Samples were run in positive polarity mode in either linear or reflection mode. High resolution mass spectra (HRMS) were recorded on a Waters QTOF mass spectrometer.

All UV-vis absorption spectra were recorded on a Varian Cary 5000 UV-vis-NIR spectrometer at room temperature in spectroscopic grade solvents. Solution phase emission spectra were recorded on a Varian Cary Eclipse Fluorimeter at room temperature in spectroscopic grade solvents, exciting at their relative absorbance maxima. Absolute quantum yield values were recorded on an Edinburgh Instruments FP920 Phosphorescence Lifetime Spectrometer equipped with a 5 watt microsecond pulsed xenon flashlamp (with single 300 mm focal length excitation and emission monochromators in Czerny Turner configuration) and a red sensitive photomultiplier in peltier (air cooled) housing and determined using a calibrated Edinburgh Instruments integrating sphere. Solid phase samples were also measured using the FP920 and integrating sphere, with steady state spectra excited via a 450 W Xe lamp.

Cyclic voltammetry was performed using a CH-Instrument 1110C Electrochemical/Analyzer potentiostat under a nitrogen flow. Measurements were made using a 1 mM analyte solution with 0.1 M tetra^{n}butylammonium hexafluorophosphate (Fluka ≥99.0 %) as the supporting electrolyte in DCM that had been degassed prior to use and obtained from a dry solvent system. A glassy carbon electrode served as the working electrode and a platinum wire as the counter electrode. An Ag/$AgNO_3$ non-aqueous reference electrode was used. All scans were calibrated against the ferrocene/ferrocenium (Fc/Fc^+) redox couple, which in this work is taken to be 5.1 eV below vacuum.[S3] The half-wave potential of the ferrocene/ferrocenium (Fc/Fc^+) redox couple ($E_{1/2,\ Fc,Fc+}$) was estimated from $E_{1/2,\ Fc,Fc+} = (E_{ap} + E_{cp})/2$, where E_{ap} and E_{cp} are the anodic and cathodic peak potentials, respectively.

Calculations were performed using the Gaussian 09 (Revision D.01) suite of programmes.[S4] Structures were optimised with DFT method at the PBE0/6-31G(d,p)/PCM(toluene) level of theory.[S5] In all cases, structures were confirmed as

minima by frequency analysis and the absence of imaginary frequencies. The S_1 geometries were optimised using time-dependent DFT and the T_1 geometries were optimised using triplet ground state. In-house Fortran 77 codes were used for calculations of SOC for $k_{(R)ISC}$, HOMO/LUMO and HONTO/LUNTO absolute overlap percentages, and ICT/LE characters. SOC values were obtained based on the equations provided by Gao *et al.*[S6] Absolute overlap integrals were numerically calculated based on Becke's grid-based integration.[S7] ICT/LE contribution percentages for each excited state were obtained via the Löwdin population analysis of NTOs; see refs S8 and S9 for more information. Full Cartesian coordinates of the optimised ground state geometries are provided in the computational section below.

Synthetic Details

Compound 1

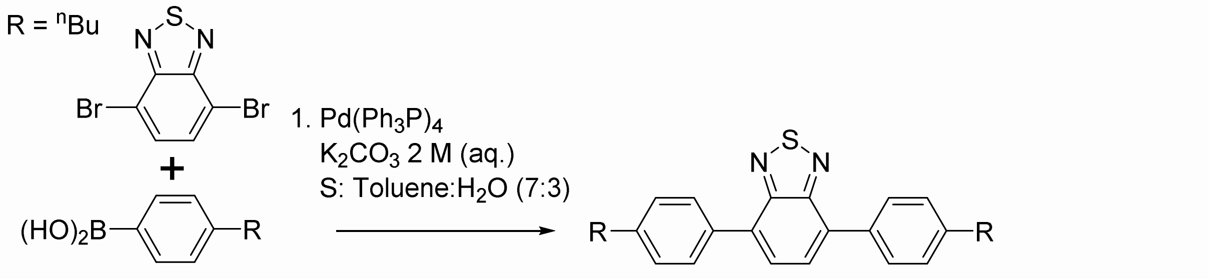

4,7-Dibromobenzo[*c*]-1,2,5-thiadiazole (3.75 g, 12.76 mmol), 4-Butylphenylboronic acid (5 g, 28.08 mmol), K_2CO_3 (7.06 g, 51.06 mmol) and $Pd(PPh_3)_4$ (444 mg, 0.32 mmol) was added to a degassed (bubble N_2) Toluene:H_2O (7:3) mixture (100 mL). The Reaction mixture was then heated at 90°C for 14 hours. After cooling to ambient temperature, 20 mL of water was added and the reaction mixture was extracted with DCM (3 x 50 mL). The isolated organic layers were then combined and the solvent was removed under reduced pressure. The resulting residue was then was purified using silica gel chromatography [eluent = 2:8 DCM: petroleum ether]. The desired product was then isolated as a crystalline yellow solid. Yield: 4.31g, 84 %.

HR-MS (APCI mode: positive): *m/z* calc. for $C_{28}H_{29}N_2S^+$ $[M + H]^+$ 401.2046, found 401.2043.

^{1}H NMR: (400 MHz, CD_2Cl_2) δ = 7.89 (d, *J* = 8.3 Hz, 4 H), 7.77 (s, 2 H), 7.36 (d, *J* = 8.1 Hz, 4 H), 2.72 (t, *J* = 7.8 Hz, 4 H), 1.69 (quin, *J* = 7.6 Hz, 4 H), 1.43 (sxt, *J* = 7.4 Hz, 4 H), 0.98 (t, *J* = 7.3 Hz, 6 H);

^{13}C{1H} NMR: (101MHz, $CDCl_3$) δ = 154.8, 143.9, 135.4, 133.5, 129.7, 129.1, 128.3, 36.0, 34.2, 23.0, 14.3;

Compound 3.

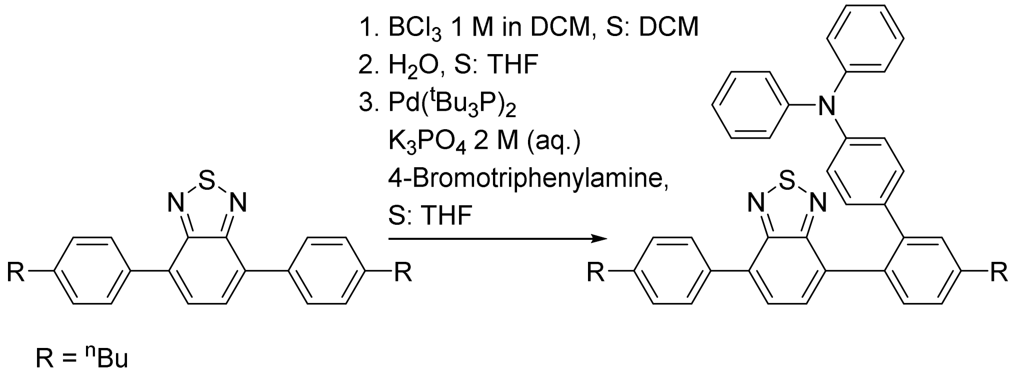

1 (250 mg, 0.62 mmol) was dissolved in anhydrous DCM (3 mL) and BCl_3 (1M in DCM) (1.25 mL, 1.25 mmol) was added to the solution where a colour change from yellow to dark purple was observed. The solution was then stirred at ambient temperature for 3 hours

under the dynamic flow of nitrogen. The solvent and other volatiles were then removed under reduced pressure and the resulting purple residue was dissolved in non-anhydrous THF (10 mL). H_2O (1 mL) was then added to the reaction mixture which was stirred overnight at ambient temperature were a colour change from purple to orange was observed. 4-Bromotriphenylamine (222 mg, 0.69 mmol) was added to the reaction mixture which was then degassed (bubble N_2). A solution of Pd($^tBu_3P)_2$ (32 mg, 0.063 mmol) in THF (5 mL) was added to the degassed reaction mixture followed by the addition of K_3PO_4 2M (aq.) (1.56 mL, 3.12 mmol). The reaction mixture was then stirred overnight at ambient temperature. The reaction mixture was diluted with ethyl acetate (50 mL) followed by the addition of brine (10 mL) and deionised water (30 mL). The organic layer was isolated using a separating funnel and dried ($MgSO_4$). The solvent was evaporated under reduced pressure and the resulting residue was purified using silica gel chromatography [eluent = 2:8 $CHCl_3$: petroleum ether]. The desired product was then isolated as a yellow solid. Yield: 342 mg, 85 %.

Reaction repeated using **1** (1.90 g, 4.74 mmol), BCl_3 (1M in DCM) (10 mL, 10 mmol), 4-Bromotriphenylamine (1.61 g, 4.98 mmol), K_3PO_4 2M (aq.) (11.85 mL, 23.70 mmol) and $Pd(PPh_3)_4$ (274 mg, 0.37 mmol) (heating overnight at 75°C). Yield: 1.98 g, 74 %.

HR-MS (APCI mode: positive): *m/z* calc. for $C_{44}H_{42}N_3S^+$ $[M + H]^+$ 644.3094, found 644.3092.

^{1}H NMR: (400 MHz, $CDCl_3$) δ = 7.86 - 7.74 (m, 2 H), 7.54 (d, *J* = 7.1 Hz, 1 H), 7.50 (d, *J* = 7.8 Hz, 1 H), 7.40 - 7.33 (m, 2 H), 7.33 - 7.27 (m, *J* = 8.3 Hz, 2 H), 7.25 (dd, *J* = 1.7, 7.8 Hz, 1 H), 7.14 - 7.06 (m, 4 H), 6.97 - 6.91 (m, 2 H), 6.91 - 6.82 (m, 6 H), 6.75 - 6.67 (m, 2 H), 2.77 - 2.57 (m, 4 H), 1.76 - 1.51 (m, 4 H), 1.46 - 1.30 (m, 4 H), 0.93 (t, *J* = 7.3 Hz, 3 H), 0.91 (t, *J* = 7.3 Hz, 3 H);

^{13}C{^{1}H} NMR: (101MHz, $CDCl_3$) δ = 155.3, 154.1, 148.2, 146.7, 144.1, 143.9, 141.9, 136.9, 135.4, 134.5, 134.4, 133.3, 131.6, 131.1, 130.7, 130.6, 129.7, 129.1, 127.9, 127.7, 124.5, 123.9, 123.2, 36.0, 36.0, 34.3, 23.1, 23.0, 14.4, 14.3;

Compound 4,

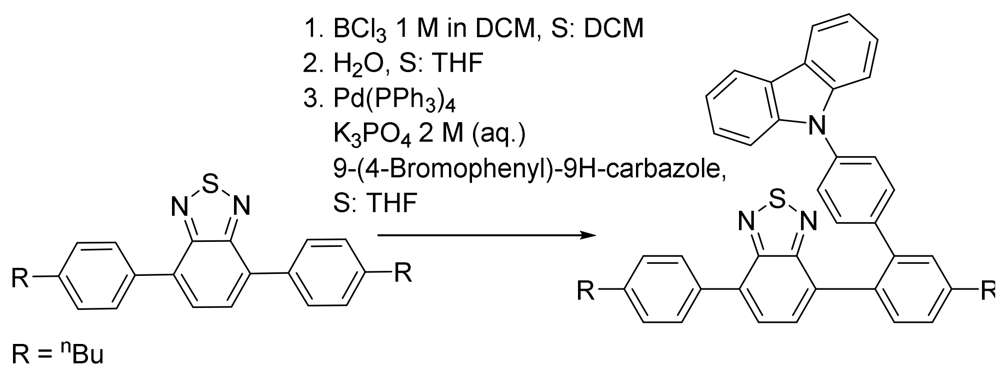

1 (279 mg, 0.70 mmol) was dissolved in anhydrous DCM (3 mL) and BCl_3 (1M in DCM) (1 mL, 1 mmol) was added to the solution where a colour change from yellow to dark purple was observed. The solution was then stirred at ambient temperature for 0.5 hours under the dynamic flow of nitrogen. The solvent and other volatiles were then removed under reduced pressure and the resulting purple residue was dissolved in non-anhydrous THF (10 mL). H_2O (2 mL) was then added to the reaction mixture which was stirred overnight at ambient temperature were a colour change from purple to orange was observed. 9-(4-Bromophenyl)-9H-carbazole (235 mg, 0.75 mmol) was added to the reaction mixture which was then degassed (bubble N_2). $Pd(PPh_3)_4$ (40 mg, 0.035 mmol) was added to the degassed reaction mixture followed by the addition of K_3PO_4 2M (aq.) (1.75 mL, 3.50 mmol). The reaction mixture was then stirred for 10 hours at 75°C. The reaction mixture was diluted with ethyl acetate (50 mL) followed by the addition of brine (10 mL) and deionised water (30 mL). The organic layer was isolated using a separating funnel and dried ($MgSO_4$). The solvent was evaporated under reduced pressure and the resulting residue was purified using silica gel chromatography [eluent = 1:9 DCM: petroleum ether graduated to 2:8 DCM: petroleum ether]. The desired product was then isolated as a yellow/green solid. Yield: 375 mg, 84 %.

HR-MS (APCI mode: positive): *m/z* calc. for $C_{44}H_{40}N_3S^+$ $[M + H]^+$ 642.2937, found 642.2938.

^{1}H NMR: (400 MHz, $CDCl_3$) δ = 8.17 (d, *J* = 7.6 Hz, 2 H), 7.95 (d, *J* = 8.3 Hz, 2 H), 7.71 (d, *J* = 7.3 Hz, 2 H), 7.59 (d, *J* = 1.5 Hz, 1 H), 7.53 (d, *J* = 7.1 Hz, 1 H), 7.50 - 7.39 (m, 7 H), 7.37 - 7.25 (m, 6 H), 2.95 - 2.83 (m, 2 H), 2.77 (t, *J* = 7.7 Hz, 2 H), 1.95 - 1.80 (m, 2 H), 1.80 - 1.67 (m, 2 H), 1.63 - 1.43 (m, 4 H), 1.09 (t, *J* = 7.3 Hz, 3 H), 1.04 (t, *J* = 7.3 Hz, 3 H);

^{13}C{^{1}H} NMR: (101MHz, $CDCl_3$) δ = 154.7, 153.5, 143.6, 143.2, 141.0, 140.6, 135.8, 134.5, 133.7, 133.3, 133.0, 131.1, 130.7, 130.6, 130.4, 129.0, 128.7, 127.8, 127.3, 126.2, 125.8, 123.2, 120.2, 119.8, 109.5, 35.5, 35.4, 33.5, 22.5, 22.3, 14.0, 13.9;

Compound 5.

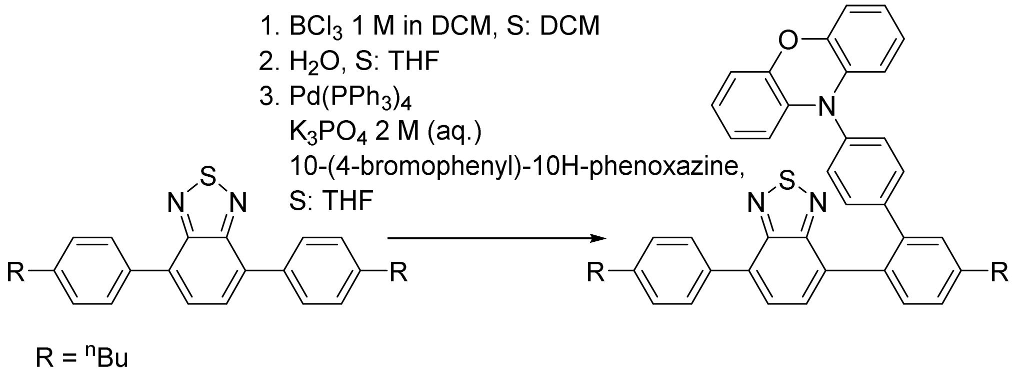

1 (323 mg, 0.81 mmol) was dissolved in anhydrous DCM (3 mL) and BCl_3 (1M in DCM) (1.2 mL, 1.2 mmol) was added to the solution where a colour change from yellow to dark purple was observed. The solution was then stirred at ambient temperature for 0.5 hours under

the dynamic flow of nitrogen. The solvent and other volatiles were then removed under reduced pressure and the resulting purple residue was dissolved in non-anhydrous THF (30 mL). H_2O (3 mL) was then added to the reaction mixture which was stirred overnight at ambient temperature were a colour change from purple to orange was observed. 10-(4-bromophenyl)-10H-phenoxazine (286 mg, 0.85 mmol) was added to the reaction mixture which was then degassed (bubble N_2). $Pd(PPh_3)_4$ (46 mg, 0.04 mmol) was added to the degassed reaction mixture followed by the addition of K_3PO_4 2M (aq.) (2.00 mL, 4.00 mmol). The reaction mixture was then stirred for 12 hours at 75°C. The reaction mixture was diluted with ethyl acetate (50 mL) followed by the addition of brine (10 mL) and deionised water (30 mL). The organic layer was isolated using a separating funnel and dried ($MgSO_4$). The solvent was evaporated under reduced pressure and the resulting residue was purified using silica gel chromatography [eluent = 2:8 DCM: petroleum]. The desired product was then isolated as a yellow solid. Yield: 468 mg, 88 %.

HR-MS (APCI mode: positive): *m/z* calc. for $C_{44}H_{40}ON_3S^+$ $[M + H]^+$ 658.2887, found 658.2893.

^{1}H NMR: (400 MHz, $CDCl_3$) δ = 7.92 - 7.79 (m, *J* = 8.1 Hz, 2 H), 7.63 (d, *J* = 7.8 Hz, 1 H), 7.66 (d, *J* = 7.3 Hz, 1 H), 7.56 - 7.46 (m, 2 H), 7.41 (dd, *J* = 1.5, 7.8 Hz, 1 H), 7.39 - 7.31 (m, 4 H), 7.09 - 6.97 (m, *J* = 8.5 Hz, 2 H), 6.70 - 6.49 (m, 6 H), 5.61 (dd, *J* = 1.5, 7.8 Hz, 2 H), 2.87 - 2.77 (m, 2 H), 2.71 (t, *J* = 7.7 Hz, 2 H), 1.85 - 1.73 (m, 2 H), 1.73 - 1.63 (m, 2 H), 1.56 - 1.47 (m, 2 H), 1.47 - 1.35 (m, 2 H), 1.03 (t, *J* = 7.3 Hz, 3 H), 0.98 (t, *J* = 7.3 Hz, 3 H);

^{13}C{^{1}H} NMR: (101MHz, $CDCl_3$) δ = 154.5, 153.5, 143.8, 143.6, 143.3, 142.3, 140.7, 137.0, 134.5, 134.2, 133.8, 133.4, 133.2, 131.8, 131.0, 130.6, 130.2, 129.9, 129.0, 128.7, 128.0, 127.2, 123.1, 121.1, 115.3, 113.0, 35.5, 35.4, 33.6, 33.5, 22.6, 22.4, 14.0, 14.0;

Compound 6

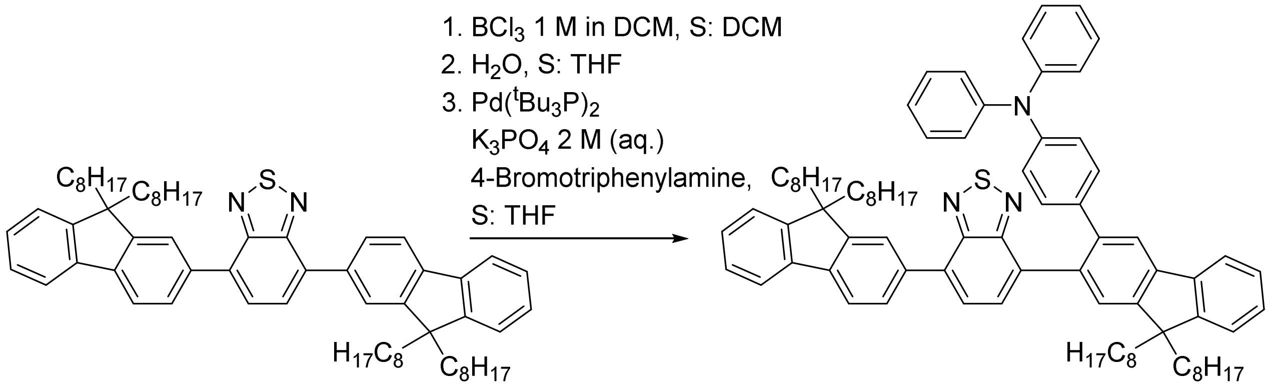

4,7-Bis(9,9-dioctyl-9H-fluoren-2-yl)-2,1,3-Benzothiadiazole (compound **2**, 294 g, 0.32 mmol) was dissolved in anhydrous DCM (5 mL) and BCl_3 (1M in DCM) (0.8 mL, 0.8 mmol) was added to the solution where a colour change from yellow to dark purple was observed. The solution was then stirred at ambient temperature for 3 hours under the dynamic flow of

nitrogen. The solvent and other volatiles were then removed under reduced pressure and the resulting purple residue was dissolved in non-anhydrous THF (10 mL). H_2O (1 mL) was then added to the reaction mixture which was stirred overnight at ambient temperature were a colour change from purple to orange was observed. 4-Bromotriphenylamine (115 mg, 0.35 mmol) was added to the reaction mixture which was then degassed (bubble N_2). A solution of Pd($^tBu_3P)_2$ (17 mg, 0.033 mmol) in THF (3 mL) was added to the degassed reaction mixture followed by the addition of K_3PO_4 2M (aq.) (0.80 mL, 1.60 mmol). The reaction mixture was then stirred overnight at ambient temperature. The reaction mixture was diluted with ethyl acetate (50 mL) followed by the addition of brine (10 mL) and deionised water (30 mL). The organic layer was isolated using a separating funnel and dried ($MgSO_4$). The solvent was evaporated under reduced pressure and the resulting residue was purified using silica gel chromatography [eluent = 1:9 DCM: petroleum ether]. The desired product was then isolated as a yellow solid. Yield: 320 mg, 86 %.

Reaction repeated using **2** (1.00 g, 1.09 mmol), BCl_3 (1M in DCM) (3 mL, 3 mmol), 4-Bromotriphenylamine (373 mg, 1.14 mmol), K_3PO_4 2M (aq.) (2.8 mL, 5.6 mmol) and $Pd(PPh_3)_4$ (63 mg, 0.05 mmol) (heating overnight at 75°C). Yield: 873 mg, 74 %.

HR-MS (APCI mode: positive): *m/z* calc. for $C_{82}H_{98}N_3S^+$ [M + H]$^+$ 1156.7476, found 1156.7490.

^{1}H NMR: (400 MHz, $CDCl_3$) δ = 7.99 (dd, *J* = 1.5, 7.8 Hz, 1 H), 7.95 - 7.91 (m, 1 H), 7.90 (s, 1 H), 7.85 (d, *J* = 8.0 Hz, 1 H), 7.80 - 7.69 (m, 3 H), 7.60 (s, 1 H), 7.56 (d, *J* = 7.3 Hz, 1 H), 7.43 - 7.25 (m, 6 H), 7.20 - 7.10 (m, 6 H), 6.99 - 6.87 (m, 6 H), 6.86 - 6.78 (m, 2 H), 2.17 - 1.87 (m, 8 H), 1.26 - 1.01 (m, 40 H), 0.96 - 0.66 (m, 20 H);

^{13}C{^{1}H} NMR: (101MHz, $CDCl_3$) δ = 154.8, 153.7, 151.5, 151.2, 151.0, 149.4, 147.6, 146.1, 141.5, 141.3, 140.7, 140.6, 140.1, 136.6, 136.1, 135.1, 134.8, 133.3, 130.5, 130.3, 129.1, 128.2, 127.3, 127.3, 127.2, 126.9, 126.8, 126.0, 123.9, 123.7, 123.5, 123.0, 122.9, 122.6, 121.3, 119.9, 119.7, 55.2, 55.1, 40.3, 40.1, 31.8, 30.1, 29.2, 29.2, 24.0, 23.9, 22.6, 22.6, 14.1, 14.1;

Compound 7

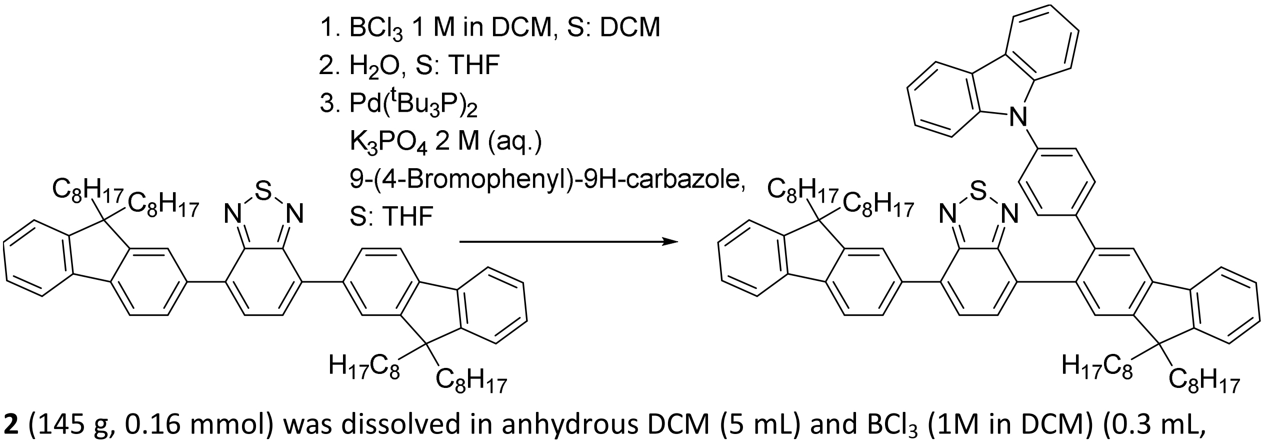

2 (145 g, 0.16 mmol) was dissolved in anhydrous DCM (5 mL) and BCl_3 (1M in DCM) (0.3 mL, 0.3 mmol) was added to the solution where a colour change from yellow to dark purple was

observed. The solution was then stirred at ambient temperature for 3 hours under the dynamic flow of nitrogen. The solvent and other volatiles were then removed under reduced pressure and the resulting purple residue was dissolved in non-anhydrous THF (10 mL). H_2O (1 mL) was then added to the reaction mixture which was stirred overnight at ambient temperature were a colour change from purple to orange was observed. 9-(4-Bromophenyl)-9H-carbazole (56 mg, 0.18 mmol) was added to the reaction mixture which was then degassed (bubble N_2). A solution of $Pd(^tBu_3P)_2$ (4 mg, 0.008 mmol) in THF (3 mL) was added to the degassed reaction mixture followed by the addition of K_3PO_4 2M (aq.) (0.40 mL, 0.80 mmol). The reaction mixture was then stirred overnight at ambient temperature. The reaction mixture was diluted with ethyl acetate (50 mL) followed by the addition of brine (10 mL) and deionised water (30 mL). The organic layer was isolated using a separating funnel and dried ($MgSO_4$). The solvent was evaporated under reduced pressure and the resulting residue was purified using silica gel chromatography [eluent = 1:9 DCM: petroleum ether]. The desired product was then isolated as a yellow solid. Yield: 136 mg, 74 %.

HR-MS (APCI mode: positive): *m/z* calc. for $C_{82}H_{96}N_3S^+$ $[M + H]^+$ 1154.7319, found 1154.7340.

^{1}H NMR: (400 MHz, $CD_2Cl_2 = CH_2Cl_2$) δ = 8.15 (d, J = 7.3 Hz, 2 H), 8.07 (d, J = 8.5 Hz, 3 H), 7.94 - 7.88 (m, 2 H), 7.86 (d, J = 7.3 Hz, 1 H), 7.84 - 7.79 (m, 1 H), 7.77 (s, 1 H), 7.68 (d, J = 7.1 Hz, 1 H), 7.55 (d, J = 8.3 Hz, 2 H), 7.52 - 7.33 (m, 10 H), 7.33 - 7.24 (m, 4 H), 2.20-2.05 (m, 8 H) 1.24 - 1.06 (m, 40 H), 1.01 - 0.72 (m, 20 H);

^{13}C{^{1}H} NMR: (101MHz, $CDCl_3$) δ = 155.5, 154.3, 152.2, 151.9, 151.7, 150.7, 142.2, 142.1, 141.9, 141.3, 141.2, 141.0, 140.4, 136.8, 136.4, 136.0, 134.9, 134.1, 131.5, 131.4, 128.9, 128.1, 128.0, 127.9, 127.6, 127.4, 126.9, 126.9, 126.5, 124.5, 123.8, 123.8, 123.6, 122.0, 120.7, 120.6, 120.5, 120.4, 120.1, 110.2, 55.8, 40.8, 40.7, 32.4, 32.4, 30.7, 30.6, 29.9, 29.8, 29.8, 24.7, 24.5, 23.3, 23.2, 14.5, 14.4;

Compound 8

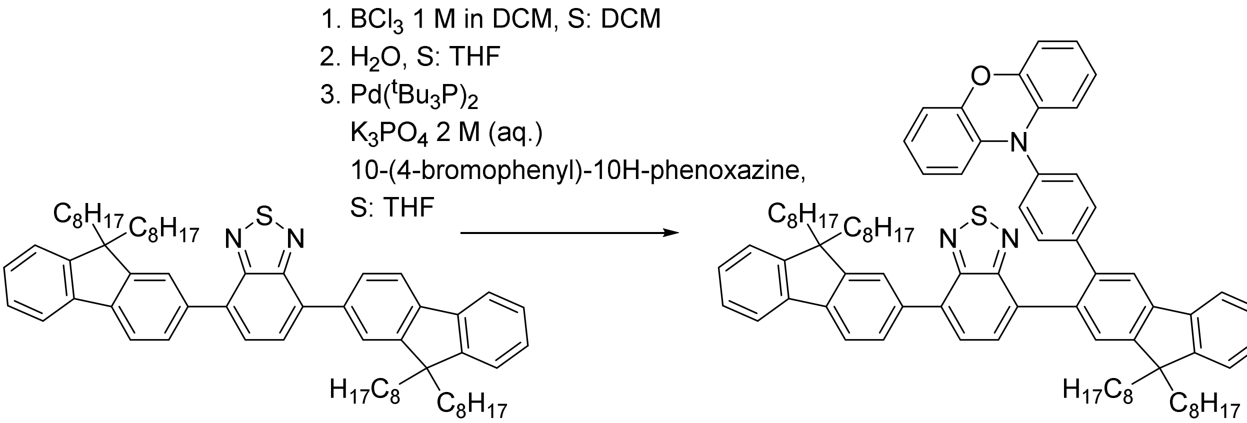

2 (186 g, 0.20 mmol) was dissolved in anhydrous DCM (5 mL) and BCl_3 (1M in DCM) (0.4 mL, 0.4 mmol) was added to the solution where a colour change from yellow to dark purple was observed. The solution was then stirred at ambient temperature for 3 hours under the

dynamic flow of nitrogen. The solvent and other volatiles were then removed under reduced pressure and the resulting purple residue was dissolved in non-anhydrous THF (10 mL). H_2O (1 mL) was then added to the reaction mixture which was stirred overnight at ambient temperature were a colour change from purple to orange was observed. 10-(4-bromophenyl)-10H-phenoxazine (72 mg, 0.21 mmol) was added to the reaction mixture which was then degassed (bubble N_2). A solution of $Pd(^tBu_3P)_2$ (5 mg, 0.010 mmol) in THF (3 mL) was added to the degassed reaction mixture followed by the addition of K_3PO_4 2M (aq.) (0.50 mL, 1.00 mmol). The reaction mixture was then stirred overnight at ambient temperature. The reaction mixture was diluted with ethyl acetate (50 mL) followed by the addition of brine (10 mL) and deionised water (30 mL). The organic layer was isolated using a separating funnel and dried ($MgSO_4$). The solvent was evaporated under reduced pressure and the resulting residue was purified using silica gel chromatography [eluent = 15:85 DCM: petroleum ether]. The desired product was then isolated as a yellow solid. Yield: 158 mg, 66 %.

HR-MS (APCI mode: positive): *m/z* calc. for $C_{82}H_{96}N_3OS^+$ $[M + H]^+$ 1170.7269, found 1170.7285.

^{1}H NMR: (400 MHz, CD_2Cl_2) δ = 8.10 - 7.96 (m, 3 H), 7.92 - 7.86 (m, 2 H), 7.86 - 7.78 (m, 2 H), 7.73 (s, 1 H), 7.69 (d, *J* = 7.3 Hz, 1 H), 7.58 - 7.32 (m, 8 H), 7.09 (d, *J* = 8.3 Hz, 2 H), 6.75 - 6.48 (m, 6 H), 5.64 (d, *J* = 6.0 Hz, 2 H), 2.21 - 1.97 (m, 8 H), 1.37 - 1.03 (m, 40 H), 1.00 - 0.71 (m, 20 H);

^{13}C{^{1}H} NMR: (101MHz, $CDCl_3$) δ = 154.8, 153.6, 151.5, 151.4, 151.1, 150.3, 143.9 (br.), 142.8, 141.7, 141.4, 140.6, 140.5, 140.0, 137.0 (br.), 136.1, 135.6, 134.4, 134.3 (br.), 133.7, 132.2, 130.7, 130.0 (br.), 128.2, 127.6, 127.4, 127.4, 127.0, 126.9, 126.0, 124.0, 123.2, 123.1, 121.2, 120.1, 119.9, 119.6, 115.2 (br.), 113.1, 55.3, 55.3, 40.2, 40.2, 31.9, 31.8, 30.1, 30.0, 29.3, 24.1, 23.9, 22.7, 22.6, 13.9, 13.9;

Compound 3-BPh$_2$

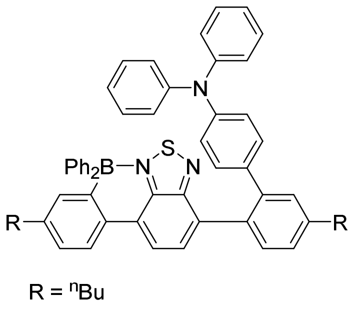

3 (48 mg, 0.075 mmol) was dissolved in anhydrous DCM (3 mL) and BCl_3 (1M in DCM) (1 mL, 1 mmol) was added to the solution where a colour change from yellow to dark red was observed. The solution was then stirred at ambient temperature for 6 hours under the

dynamic flow of nitrogen where upon the solution had become a dark purple colour. The solvent and other volatiles were then removed under reduced pressure and the resulting purple residue was dissolved in DCM (3mL). $ZnPh_2$ (36 mg, 0.164 mmol) was then added to the solution and the reaction mixture was stirred for 3 hours at ambient temperature. The reaction mixture was then passed through a plug of silica gel (eluent DCM) with only the red coloured fractions retained. The solvent was then removed under reduced pressure to give the desired product as a dark red solid. Yield: 55 mg, 91 %.

^{1}H NMR: (400 MHz, $CDCl_3$) δ = 8.16 (d, *J* = 7.8 Hz, 1 H), 8.00 (d, *J* = 8.3 Hz, 1 H), 7.64 (d, *J* = 7.5 Hz, 1 H), 7.55 (d, *J* = 7.8 Hz, 1 H), 7.41 (dd, *J* = 1.5, 20.0 Hz, 2 H), 7.35 (dd, *J* = 1.8, 7.8 Hz, 1 H), 7.26 - 7.12 (m, 15 H), 7.07 - 6.97 (m, 8 H), 6.89 - 6.81 (m, 2 H), 2.84 - 2.72 (m, 2 H), 2.61 (t, *J* = 7.7 Hz, 2 H), 1.82 - 1.70 (m, 2 H), 1.67 - 1.55 (m, 2 H), 1.55 - 1.43 (m, 3 H overlapping with water in $CDCl_3$, should be 2H), 1.40 - 1.29 (m, 2 H), 1.02 (t, *J* = 7.4 Hz, 3 H), 0.92 (t, *J* = 7.4 Hz, 3 H);

^{13}C{^{1}H} NMR: (101MHz, $CDCl_3$) δ = 154.5 (br.), 154.0, 152.9 (br.), 147.6, 147.4, 146.6, 144.1, 143.5, 141.3, 135.1, 134.8, 133.6, 133.4, 132.9, 131.5, 131.0, 130.6, 129.9, 129.2, 128.6, 128.0, 127.5, 127.3, 126.5, 125.8, 124.5, 123.2, 123.0, 122.4, 122.1, 35.7, 35.5, 33.5, 33.4, 22.5, 22.3, 14.0, 13.9;

^{11}B NMR: (1280 MHz, $CDCl_3$) δ = 1.66;

HR-MS (APCI mode: positive): *m/z* calc. for $C_{56}H_{51}N_3BS^+$ $[M + H]^+$ 808.3891, found 808.3898.

Compound 9

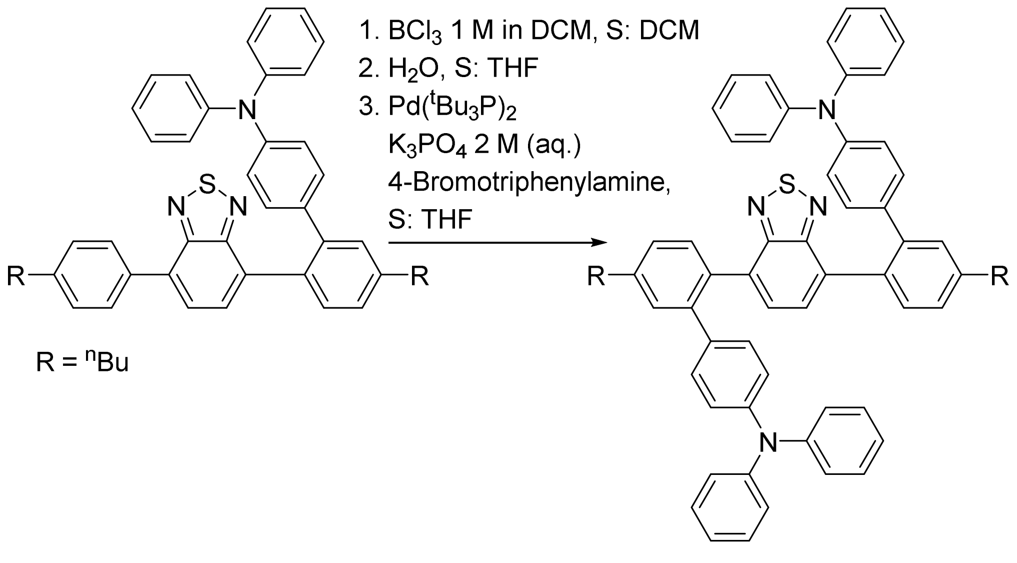

3 (205 mg, 0.32 mmol) was dissolved in anhydrous DCM (3 mL) and BCl_3 (1M in DCM) (1 mL, 1 mmol) was added to the solution where a colour change from yellow to dark red was observed. The solution was then stirred at ambient temperature for 6 hours under the dynamic flow of nitrogen where upon the solution had become a dark purple colour. The

solvent and other volatiles were then removed under reduced pressure and the resulting purple residue was dissolved in non-anhydrous THF (10 mL). H_2O (1 mL) was then added to the reaction mixture which was stirred overnight at ambient temperature were a colour change from purple to orange was observed. 4-Bromotriphenylamine (114 mg, 0.35 mmol) was added to the reaction mixture which was then degassed (bubble N_2). A solution of $Pd(^tBu_3P)_2$ (8 mg, 0.016 mmol) in THF (3 mL) was added to the degassed reaction mixture followed by the addition of K_3PO_4 2M (aq.) (0.90 mL, 1.80 mmol). The reaction mixture was then stirred overnight at ambient temperature. The reaction mixture was diluted with ethyl acetate (50 mL) followed by the addition of brine (10 mL) and deionised water (30 mL). The organic layer was isolated using a separating funnel and dried ($MgSO_4$). The solvent was evaporated under reduced pressure and the resulting residue was purified using silica gel chromatography [eluent = 2:8 $CHCl_3$: petroleum ether graduated to 4:6 $CHCl_3$: petroleum ether]. The desired product was then isolated as a yellow solid. Yield: 217 mg, 77 %.

HR-MS (APCI mode: positive): *m/z* calc. for $C_{62}H_{55}N_4S^+$ $[M + H]^+$ 887.4142, found 887.4146.

^{1}H NMR: (400 MHz, CD_2Cl_2) δ = 7.52 (d, *J* = 7.6 Hz, 2 H), 7.49 - 7.44 (m, 2 H), 7.40 - 7.32 (m, 4 H), 7.18 (t, *J* = 7.9 Hz, 8 H), 6.96 (dd, *J* = 5.9, 7.8 Hz, 8 H), 6.91 (d, *J* = 7.8 Hz, 8 H), 6.65 (d, *J* = 8.5 Hz, 4 H), 2.84 (t, *J* = 7.7 Hz, 4 H), 1.82 (quin, *J* = 7.6 Hz, 4 H), 1.55 (sxt, *J* = 7.4 Hz, 4 H), 1.08 (t, *J* = 7.3 Hz, 6 H);

^{13}C{^{1}H} NMR: (101MHz, CD_2Cl_2) δ = 154.7, 148.2, 146.8, 144.0, 141.8, 136.7, 134.4, 134.4, 131.6, 130.7, 130.6, 130.6, 129.7, 127.6, 124.4, 124.0, 123.1, 36.1, 34.3, 23.2, 14.5;

Compound 10

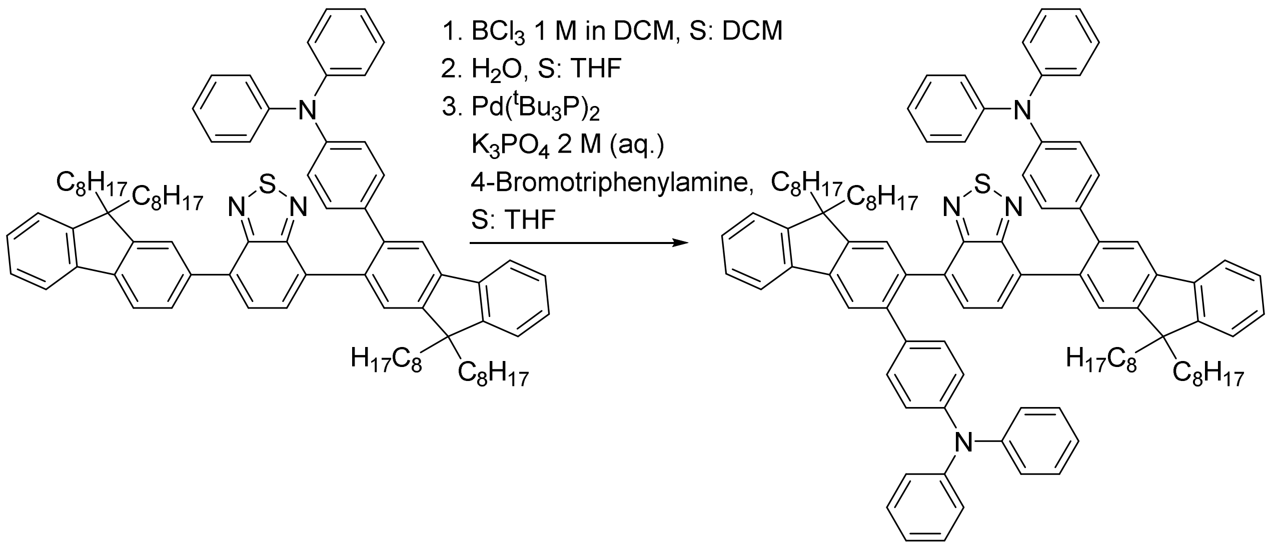

6 (380 mg, 0.35 mmol) was dissolved in anhydrous DCM (5 mL) and BCl_3 (1M in DCM) (0.75 mL, 0.75 mmol) was added to the solution where a colour change from yellow to dark red was observed. The solution was then stirred at ambient temperature for 6 hours under the

dynamic flow of nitrogen where upon the solution had become a dark purple colour. The solvent and other volatiles were then removed under reduced pressure and the resulting purple residue was dissolved in non-anhydrous THF (10 mL). H_2O (1 mL) was then added to the reaction mixture which was stirred overnight at ambient temperature were a colour change from purple to orange was observed. 4-Bromotriphenylamine (120 mg, 0.37 mmol) was added to the reaction mixture which was then degassed (bubble N_2). A solution of $Pd(^tBu_3P)_2$ (9 mg, 0.018 mmol) in THF (5 mL) was added to the degassed reaction mixture followed by the addition of K_3PO_4 2M (aq.) (1.56 mL, 3.12 mmol). The reaction mixture was then stirred overnight at ambient temperature. The reaction mixture was diluted with ethyl acetate (50 mL) followed by the addition of brine (10 mL) and deionised water (30 mL). The organic layer was isolated using a separating funnel and dried ($MgSO_4$). The solvent was evaporated under reduced pressure and the resulting residue was purified using silica gel chromatography [eluent = 1:9 DCM: pentane]. The desired product was then isolated as a yellow solid. Yield: 367 mg, 76 %.

Reaction repeated using **6** (172 mg, 0.15 mmol), BCl_3 (1M in DCM) (0.3 mL, 0.3 mmol), 4-Bromotriphenylamine (54 mg, 0.16 mmol), K_3PO_4 2M (aq.) (0.35 mL, 0.70 mmol) and $Pd(PPh_3)_4$ (9 mg, 0.008 mmol) (heating overnight at 75°C). Yield: 128 mg, 62 %.

HR-MS (APCI mode: positive): *m/z* calc. for $C_{100}H_{111}N_4S^+$ $[M + H]^+$ 1399.8524, found 1399.8546.

^{1}H NMR: (400 MHz, CD_2Cl_2) δ = 7.91 (s, 2 H), 7.87 - 7.78 (m, 2 H), 7.60 (s, 2 H), 7.49 - 7.34 (m, 8 H), 7.21 - 7.09 (m, 8 H), 7.00 (d, *J* = 8.5 Hz, 4 H), 6.96 - 6.80 (m, 12 H), 6.65 (d, *J* = 8.5 Hz, 4 H), 2.16 - 1.93 (m, 8 H), 1.27 - 1.08 (m, 40 H), 0.96 - 0.76 (m, 20 H);

^{13}C{^{1}H} NMR: (101MHz, $CDCl_3$) δ = 154.2, 151.5, 149.3, 147.6, 146.1, 141.3, 140.6, 140.1, 136.5, 135.1, 134.5, 130.2, 130.1, 129.1, 127.3, 126.9, 125.8, 123.8, 123.5, 123.0, 122.5, 121.2, 119.9, 55.0, 40.1, 31.8, 30.1, 29.2, 29.2, 24.0, 22.6, 14.1;

Compound 11

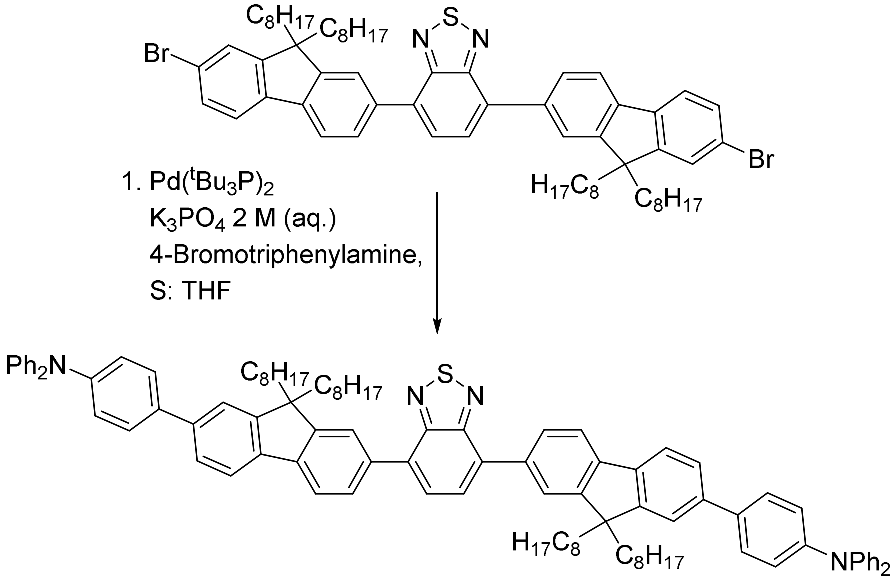

4,7-bis(7-bromo-9,9-dioctyl-9H-fluoren-2-yl)-2,1,3-Benzothiadiazole (200 mg, 0.19 mmol) and (4-(diphenylamino)phenyl)boronic acid was dissolved in degassed (bubble N_2) THF (10 mL). A solution of Pd(^{t}Bu$_3$P)$_2$ (10 mg, 0.02 mmol) in THF (3 mL) was added to the degassed reaction mixture followed by the addition of K_3PO_4 2M (aq.) (1.0 mL, 2.0 mmol). The reaction mixture was then stirred overnight at ambient temperature. The reaction mixture was diluted with ethyl acetate (50 mL) followed by the addition of brine (10 mL) and deionised water (30 mL). The organic layer was isolated using a separating funnel and dried ($MgSO_4$). The solvent was evaporated under reduced pressure and the resulting residue was purified using silica gel chromatography [eluent = 15:85 DCM: petroleum ether graduated to 30:70 DCM: petroleum ether]. The desired product was then isolated as an orange solid. Yield: 195 mg, 74 %.

HR-MS (APCI mode: positive): *m/z* calc. for $C_{100}H_{111}ON_3S^+$ $[M + H]^+$ 1399.8524, found 1399.8533

^{1}H NMR: (400 MHz, $CDCl_3$) δ = 8.09 (dd, J = 1.2, 8.1 Hz, 2 H), 8.03 (s, 2 H), 7.98 - 7.89 (m, 4 H), 7.85 (d, J = 8.3 Hz, 2 H), 7.70 - 7.58 (m, 8 H), 7.37 - 7.29 (m, 8 H), 7.26 - 7.18 (m, 12 H), 7.12 - 7.05 (m, 4 H), 2.27 - 2.00 (m, 8 H), 1.29 - 1.11 (m, 40 H), 0.95 - 0.79 (m, 20 H);

^{13}C{^{1}H} NMR: (101MHz, $CDCl_3$) δ = 154.3, 152.0, 151.3, 147.7, 147.1, 141.1, 139.7, 139.5, 136.1, 135.6, 133.5, 129.3, 128.2, 127.9, 127.8, 125.6, 124.4, 124.0, 123.9, 122.9, 121.0, 120.2, 119.7, 55.3, 40.3, 31.8, 30.1, 29.2, 29.2, 23.9, 22.6, 14.1;

NMR spectra

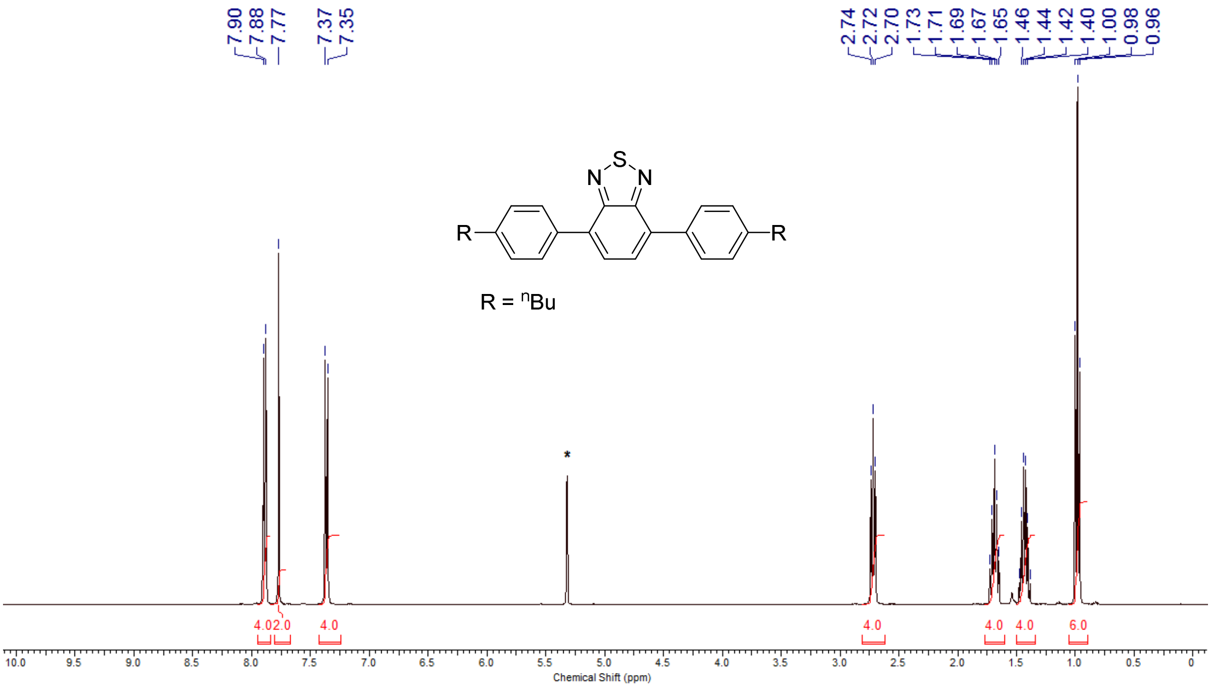

^{1}**H NMR** spectrum of **1** in CD_2Cl_2. * = CH_2Cl_2

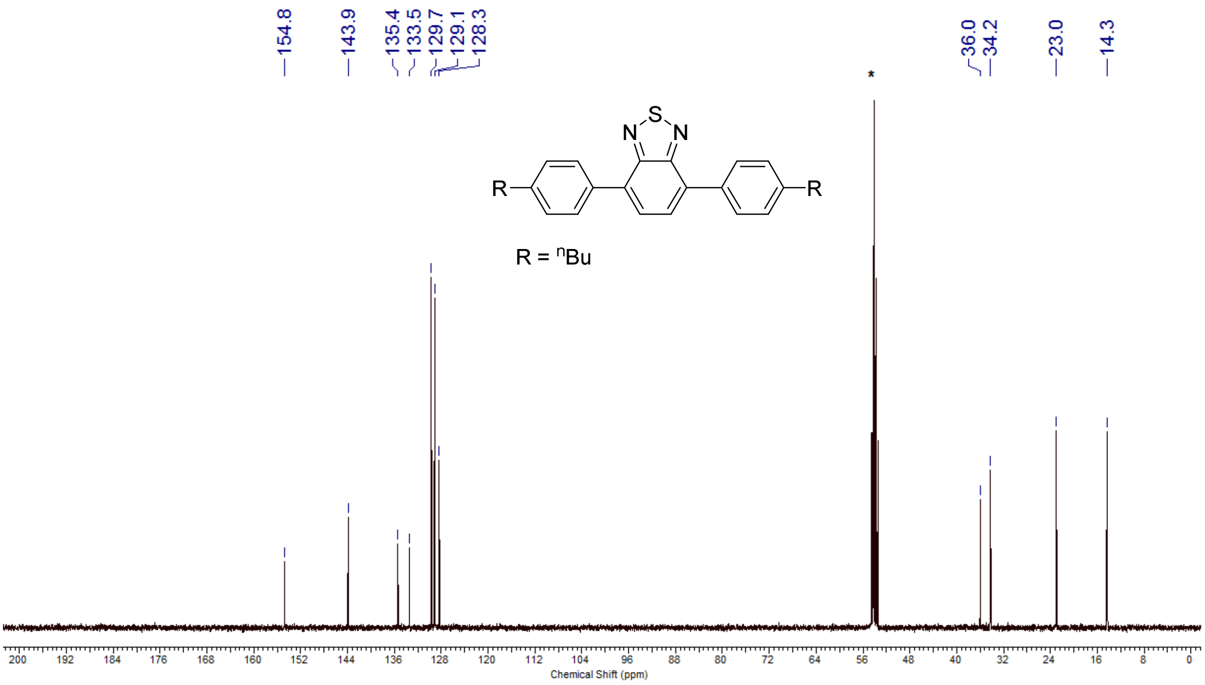

^{13}**C{^{1}H} NMR** spectrum of **1** in CD_2Cl_2. * = CD_2Cl_2

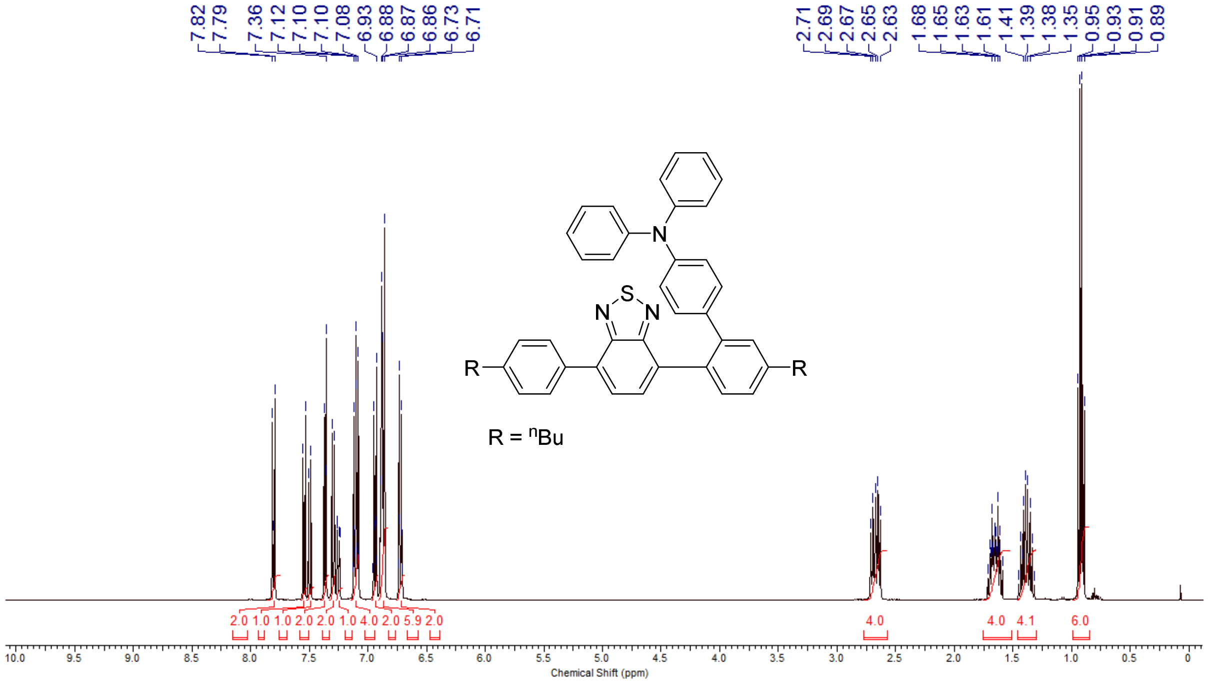

^{1}**H NMR** spectrum of ***3*** in $CDCl_3$.

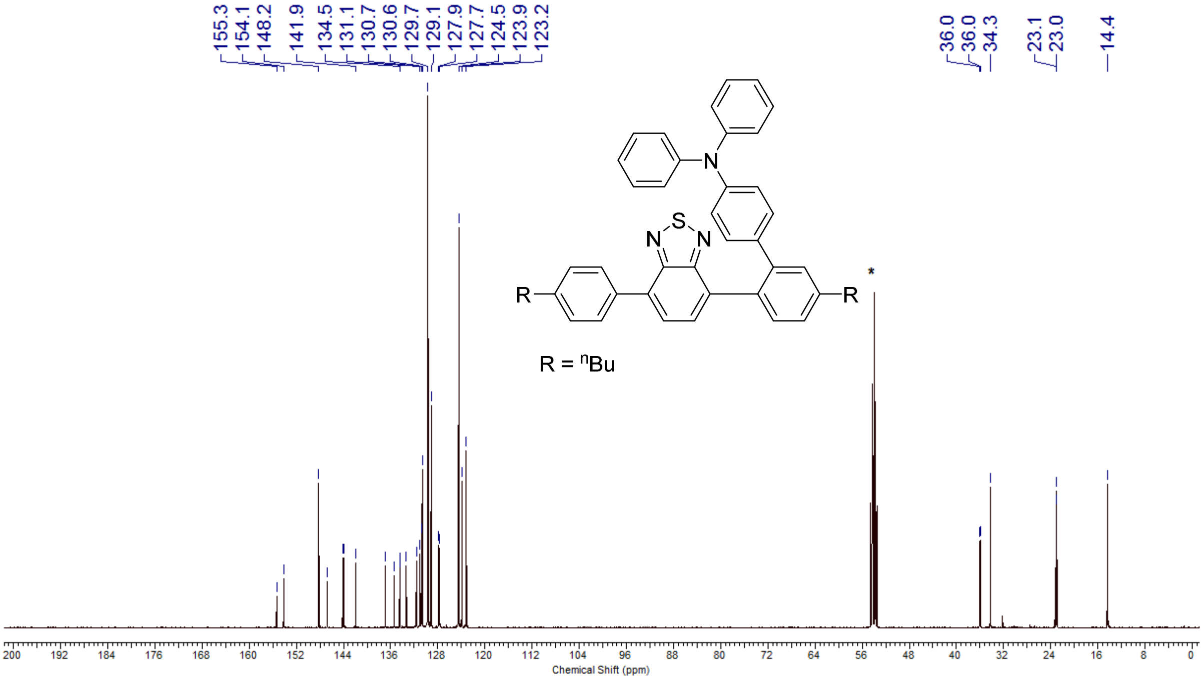

^{13}**C{^{1}H} NMR** spectrum of ***3*** in CD_2Cl_2. * = CD_2Cl_2

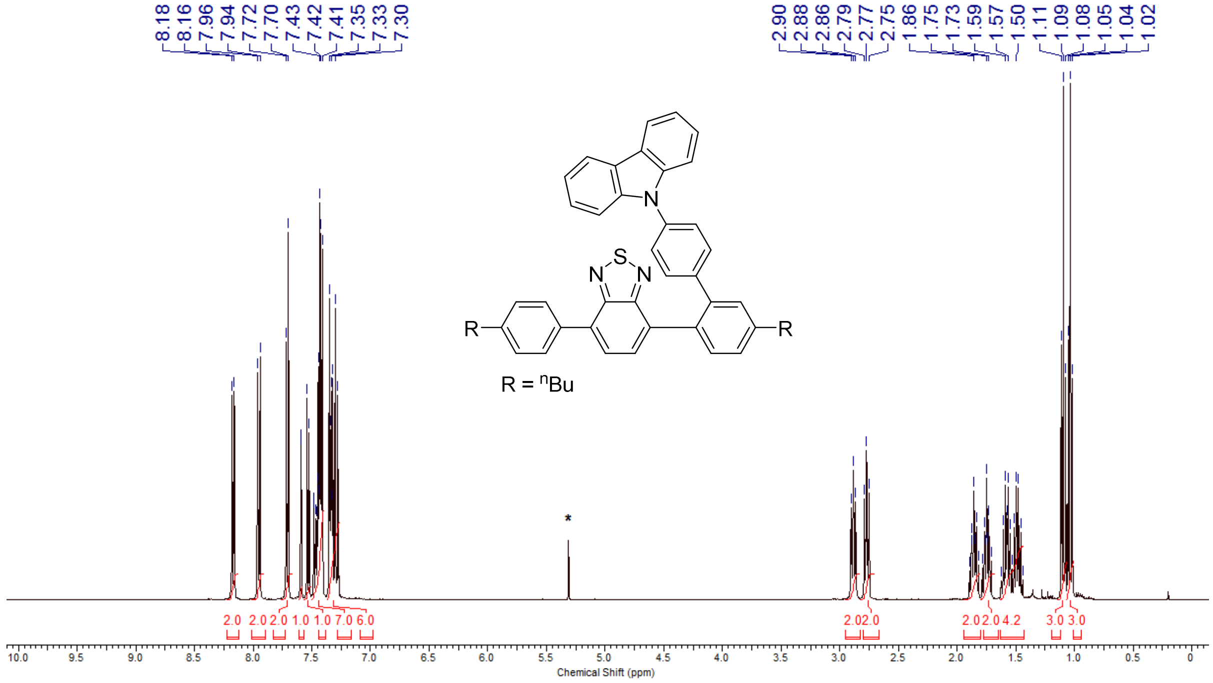

^{1}**H NMR** spectrum of ***4*** in $CDCl_3$. * = CH_2Cl_2

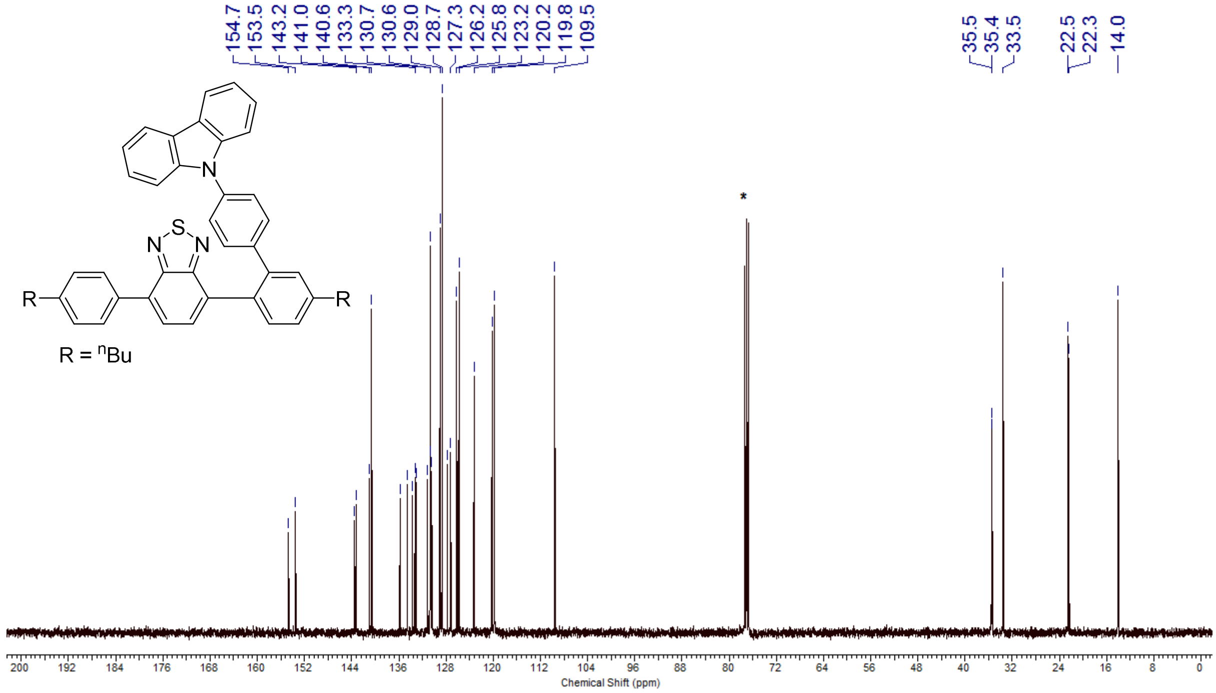

^{13}**C{^{1}H} NMR** spectrum of ***4*** in $CDCl_3$. * = $CDCl_3$

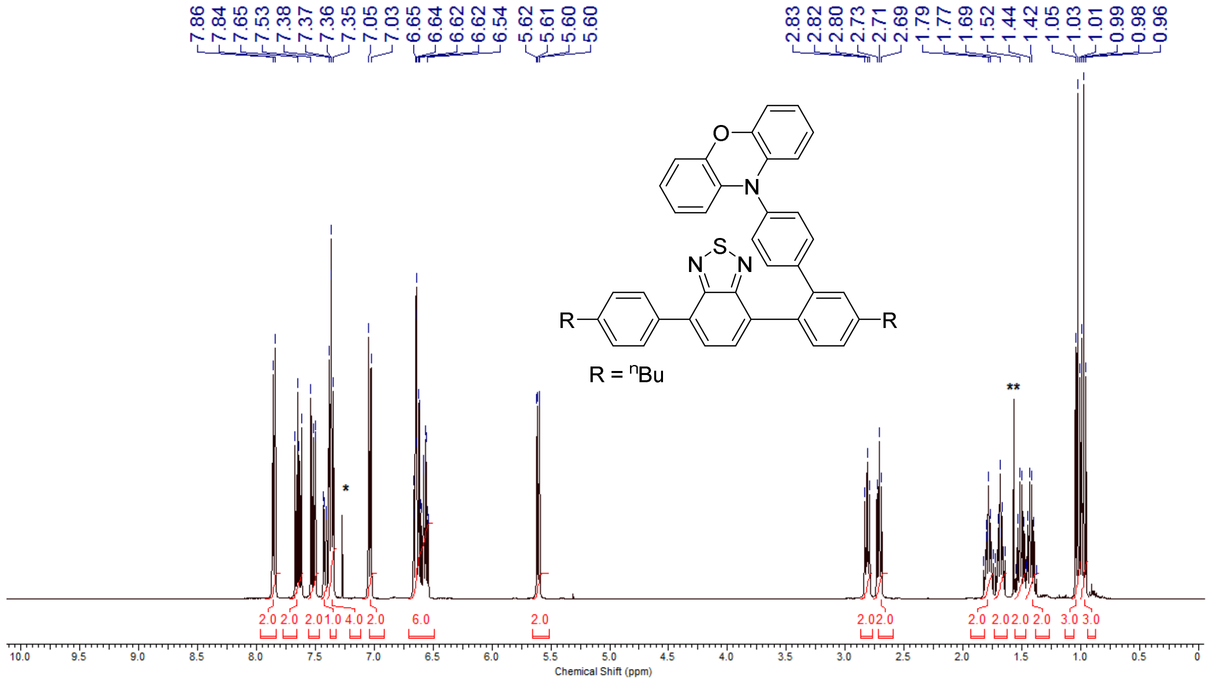

^{1}**H NMR** spectrum of ***5*** in $CDCl_3$. * = $CHCl_3$, ** = H_2O

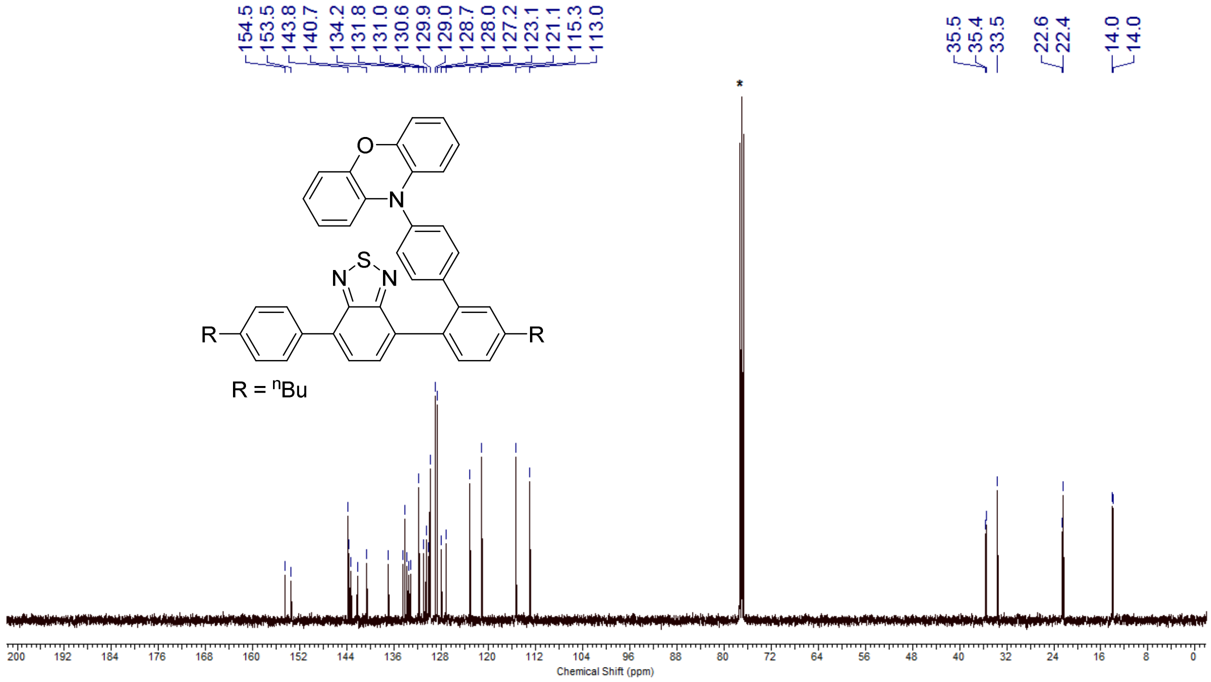

^{13}**C{^{1}H} NMR** spectrum of ***5*** in $CDCl_3$. * = $CDCl_3$

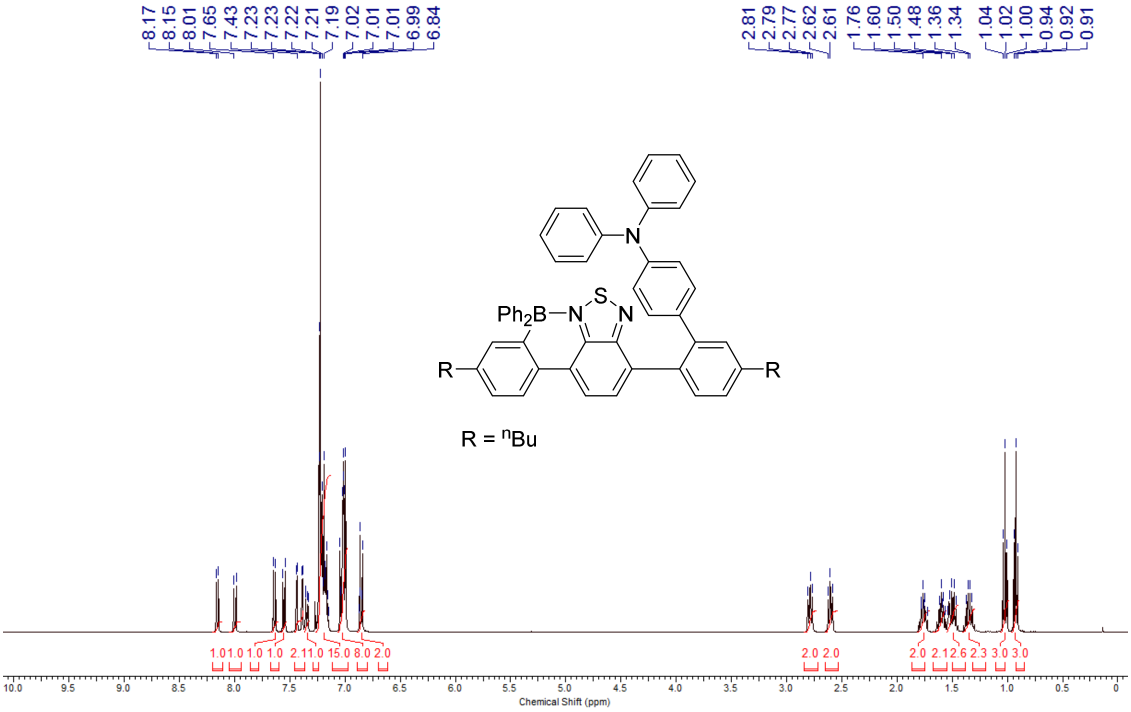

^{1}**H NMR** spectrum of **3-BPh$_2$** in $CDCl_3$.

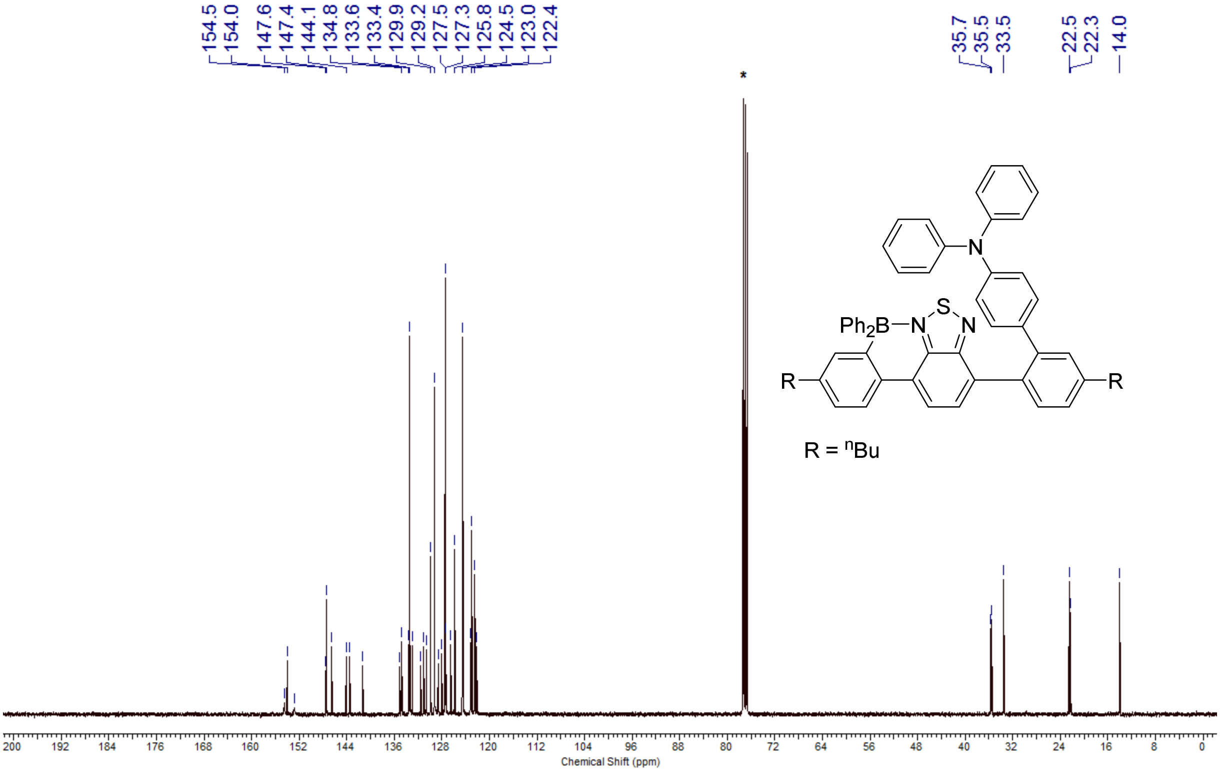

^{13}**C{^{1}H} NMR** spectrum of **3-BPh$_2$** in $CDCl_3$. * = $CDCl_3$

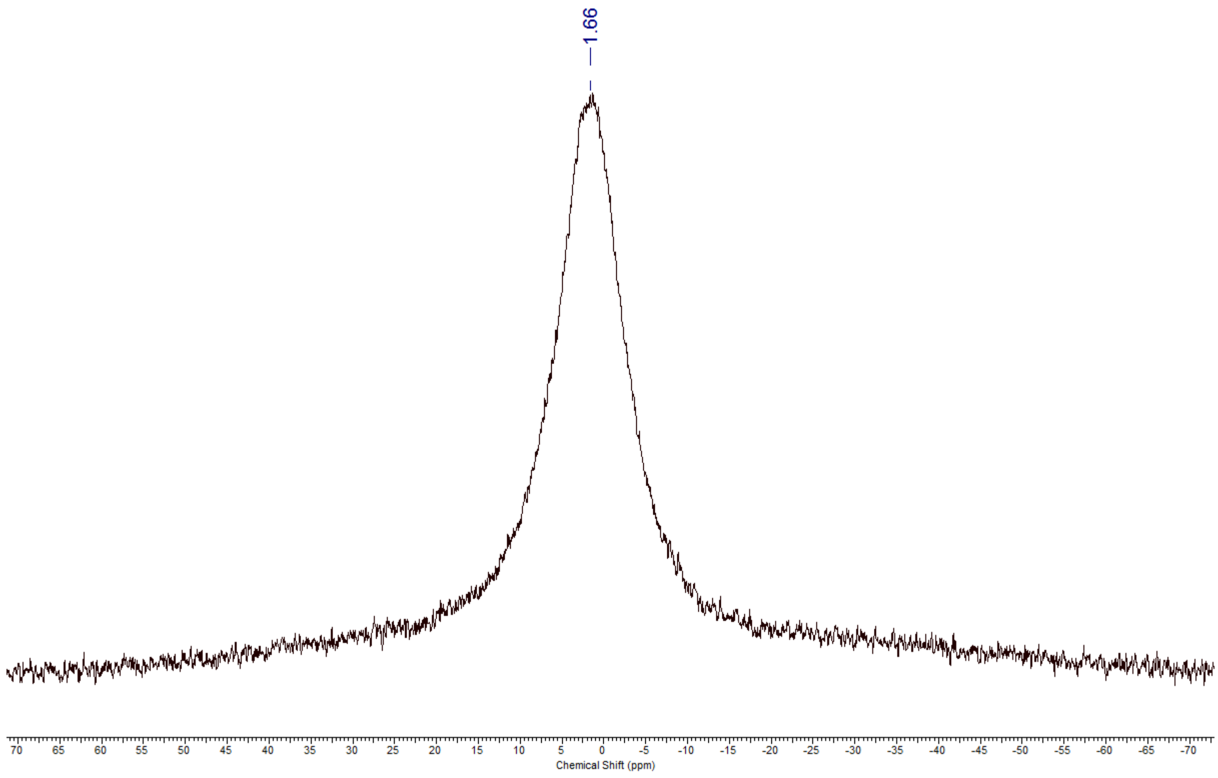

^{11}B NMR spectrum of **3-BPh$_2$** in $CDCl_3$ (run in a quartz NMR tube).

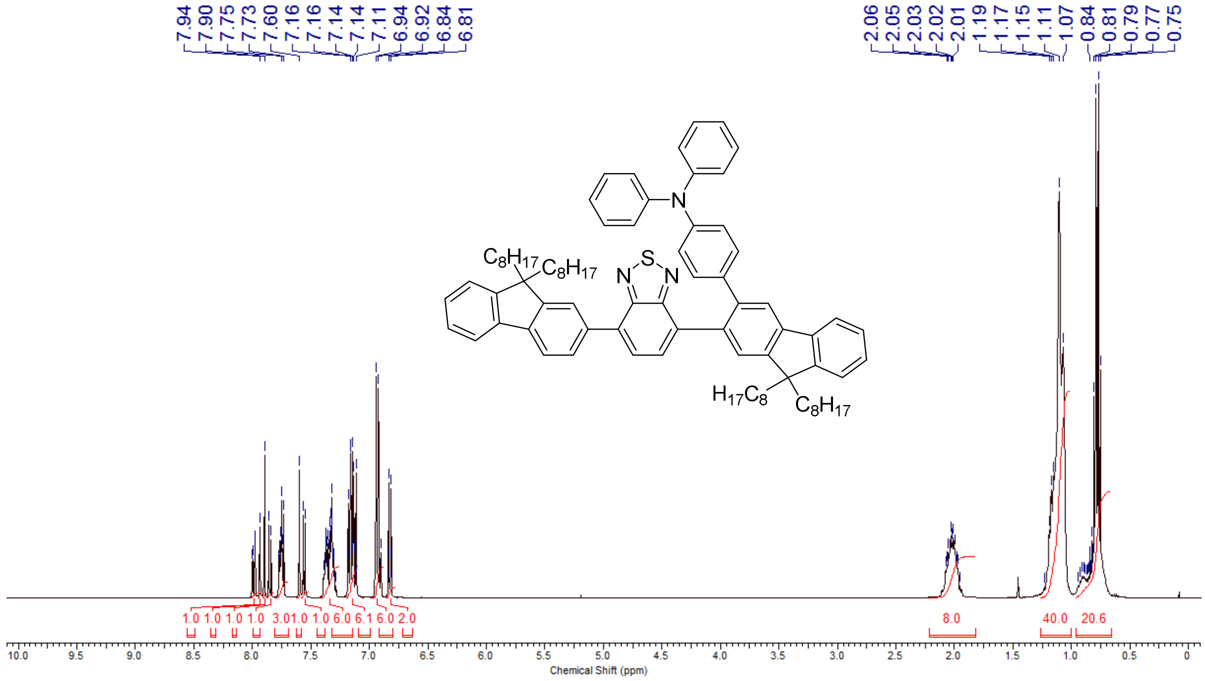

^{1}H NMR spectrum of ***6*** in $CDCl_3$.

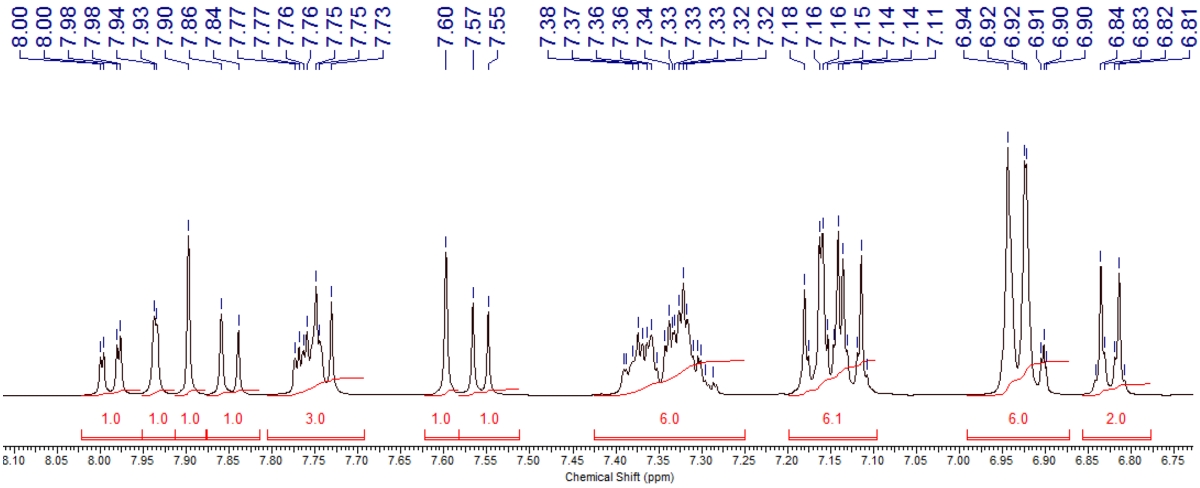

^{1}H NMR spectrum of ***6*** in $CDCl_3$ 8.10 ppm to 6.75 ppm.

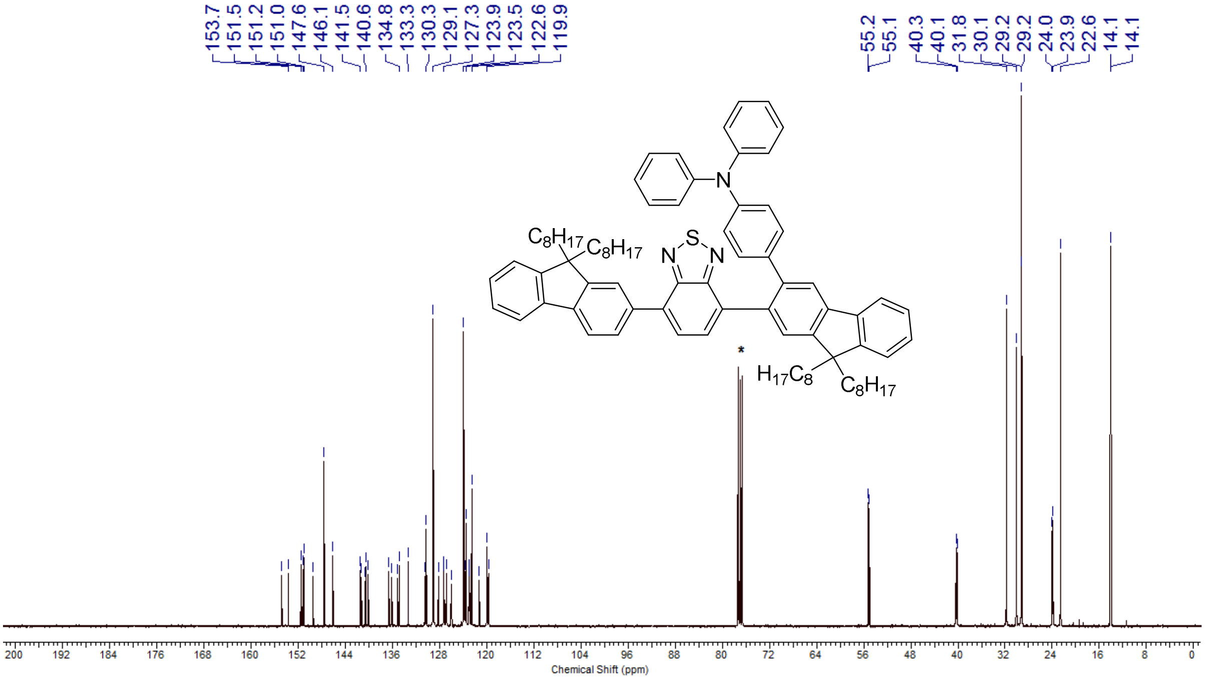

^{13}C{^{1}H} NMR spectrum of ***6*** in $CDCl_3$. * = $CDCl_3$

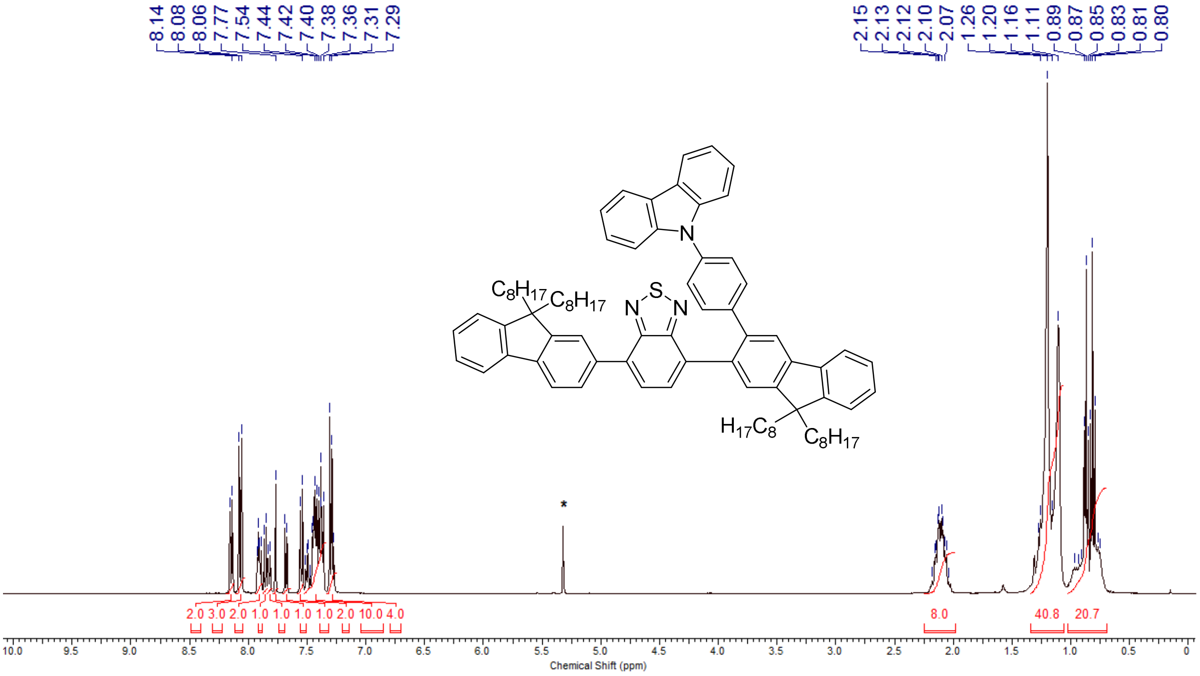

^{1}H NMR spectrum of ***7*** in CD_2Cl_2. * = CH_2Cl_2

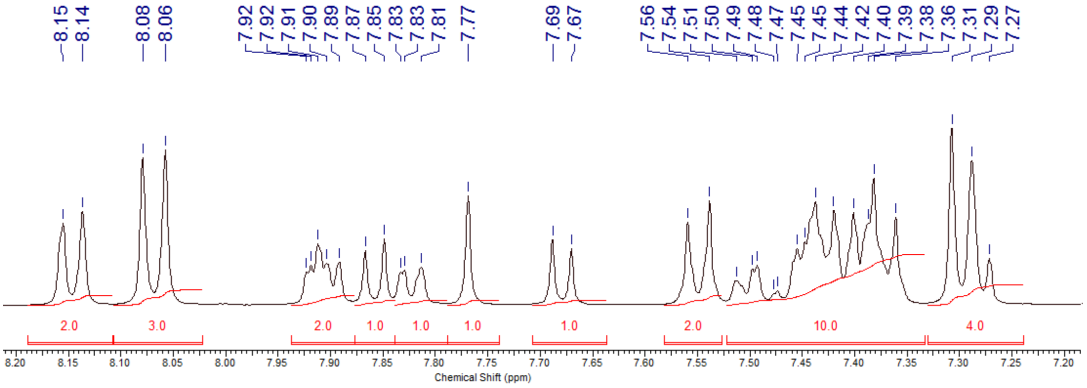

^{1}H NMR spectrum of ***7*** in CD_2Cl_2 8.20 ppm to 7.20 ppm region.

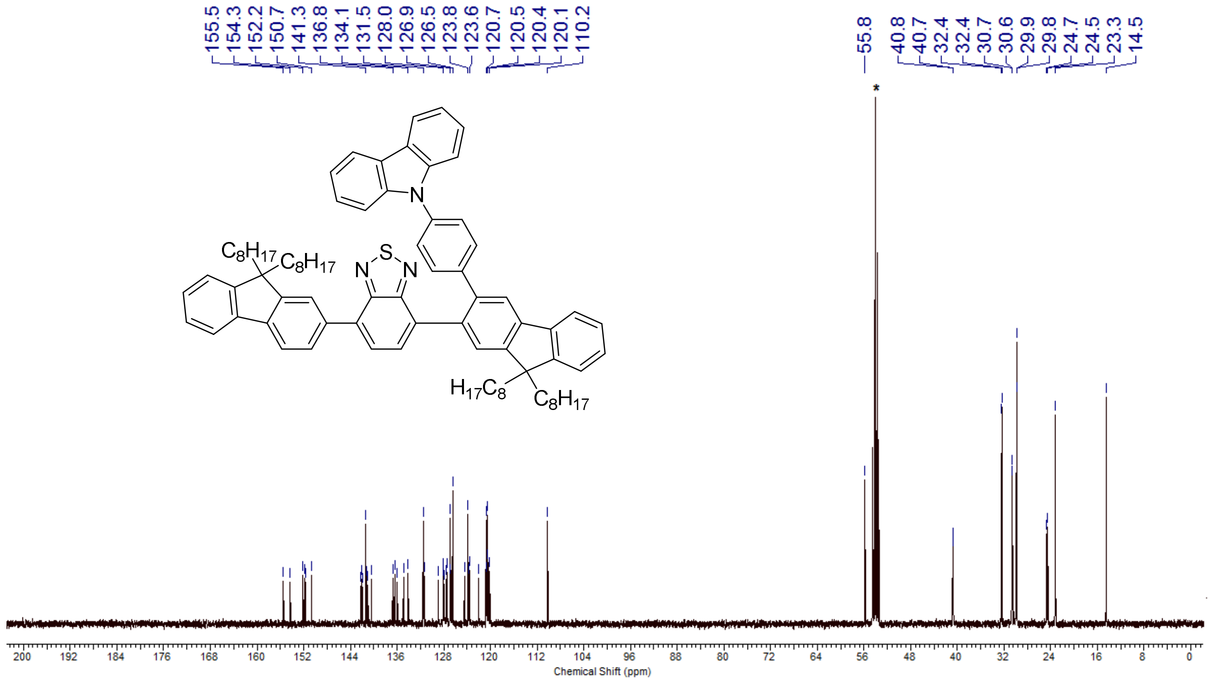

$^{13}C\{^{1}H\}$ NMR spectrum of ***7*** in CD_2Cl_2. * = CD_2Cl_2

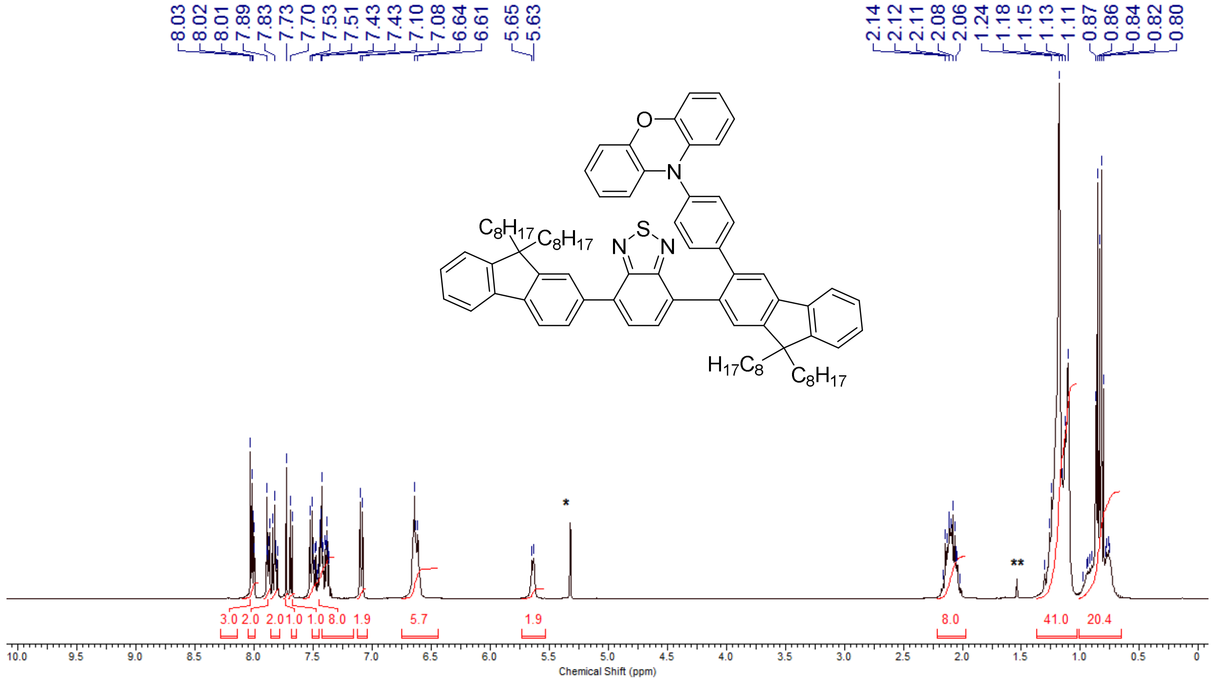

^{1}H NMR spectrum of ***8*** in CD_2Cl_2. * = CH_2Cl_2, ** = H_2O

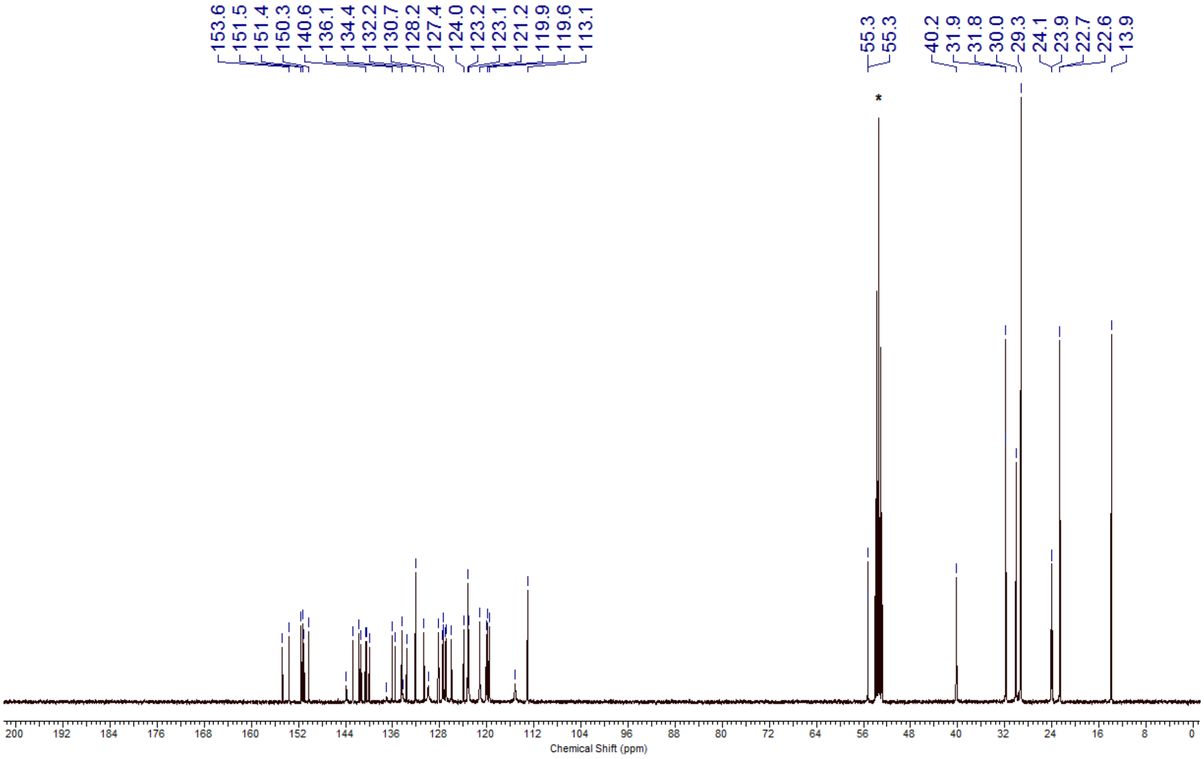

$^{13}C\{^1H\}$ NMR spectrum of ***8*** in CD_2Cl_2. * = CD_2Cl_2

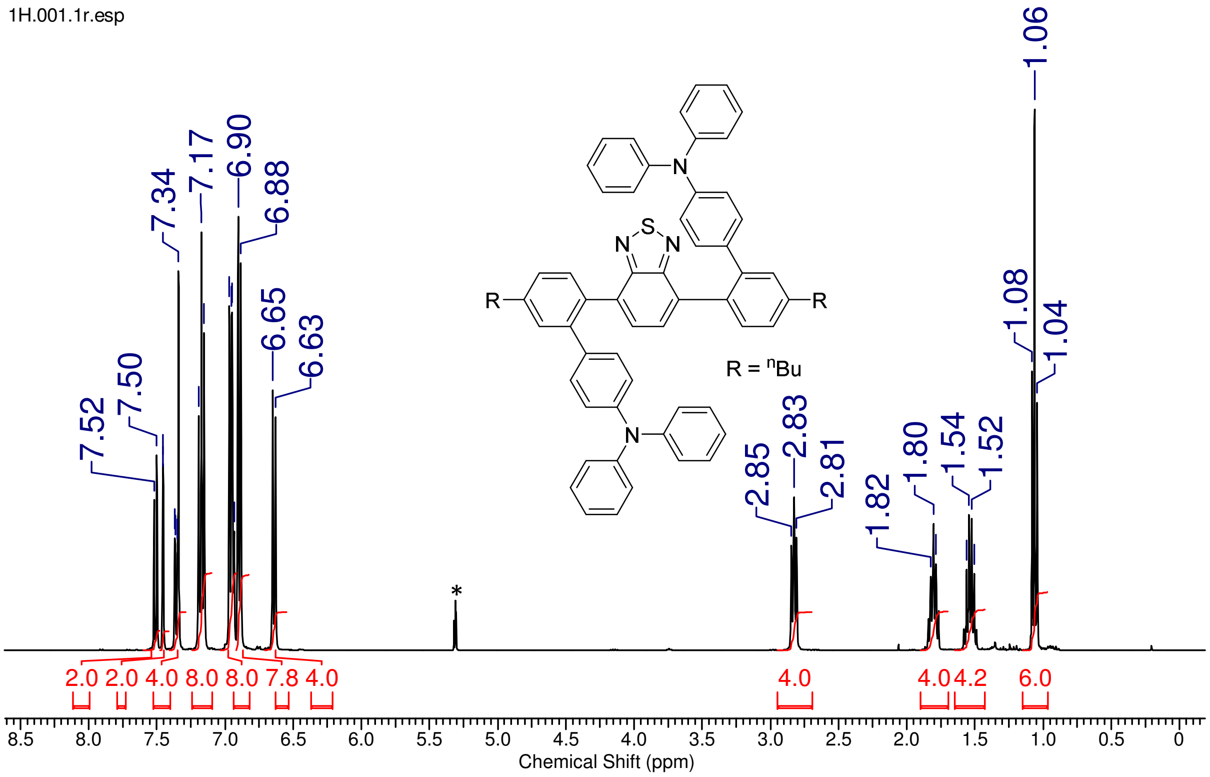

^1H NMR spectrum of ***9*** in CD_2Cl_2. * = CH_2Cl_2

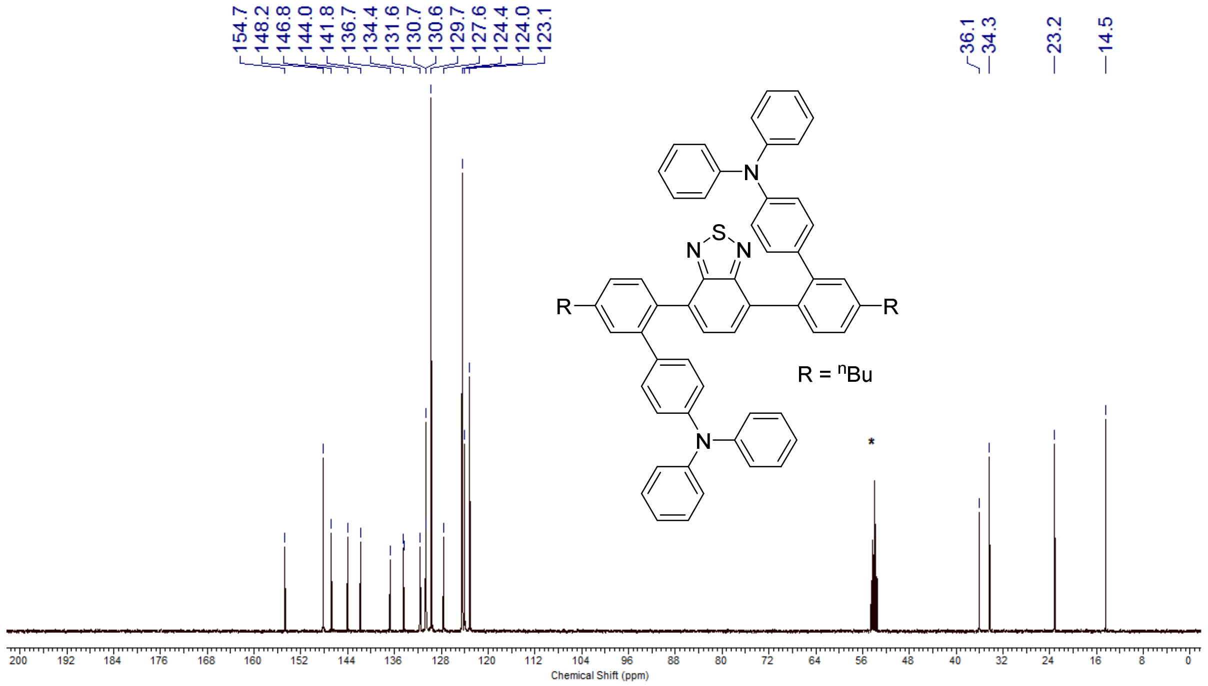

^{13}C{^{1}H} NMR spectrum of ***9*** in CD_2Cl_2. * = CH_2Cl_2

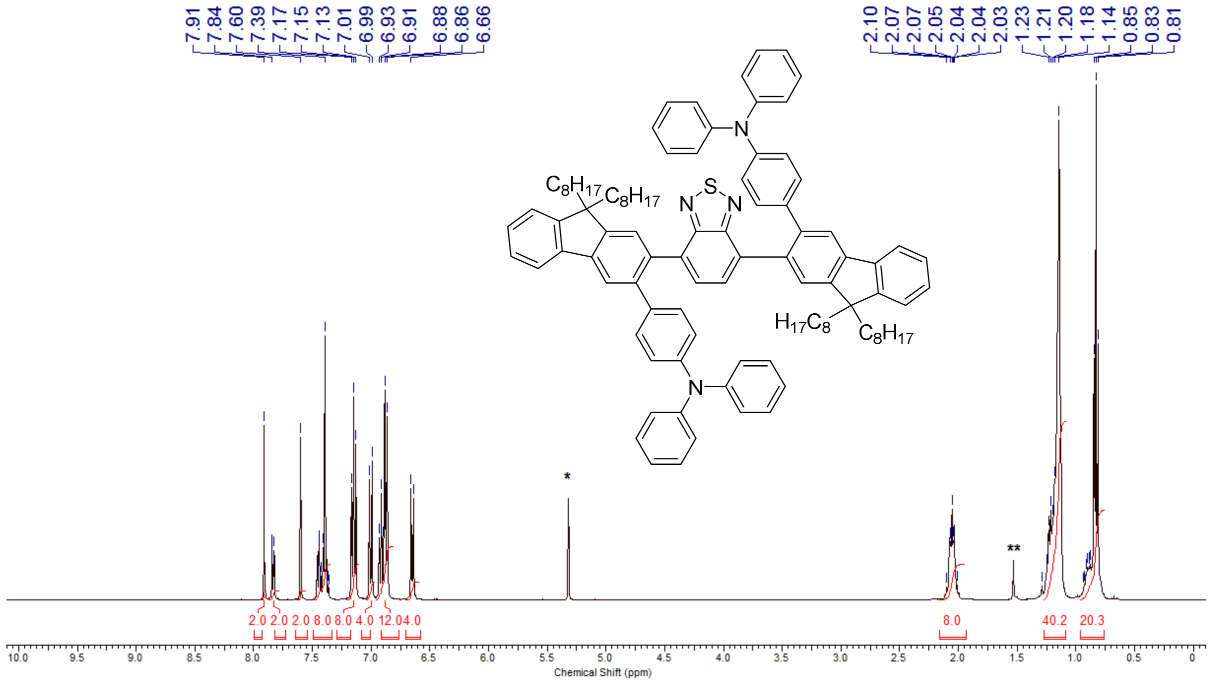

^{1}H NMR spectrum of ***10*** in CD_2Cl_2. * = CH_2Cl_2, ** = H_2O

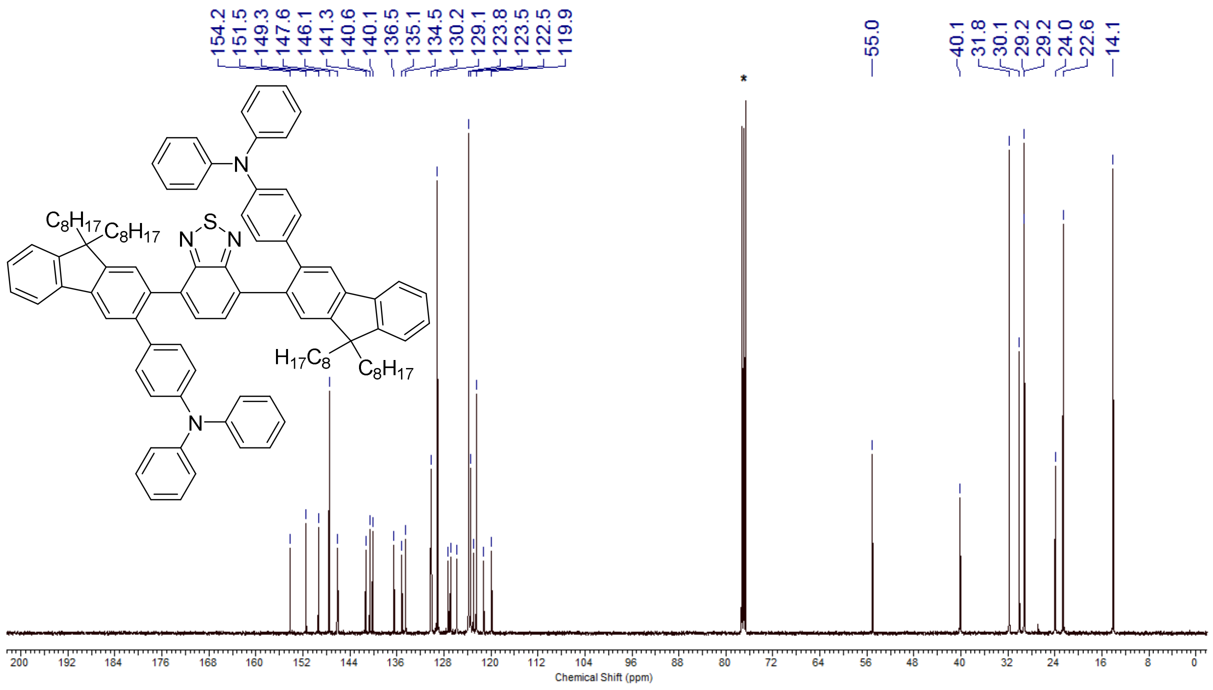

$^{13}C\{^1H\}$ **NMR** spectrum of ***10*** in $CDCl_3$. * = $CDCl_3$

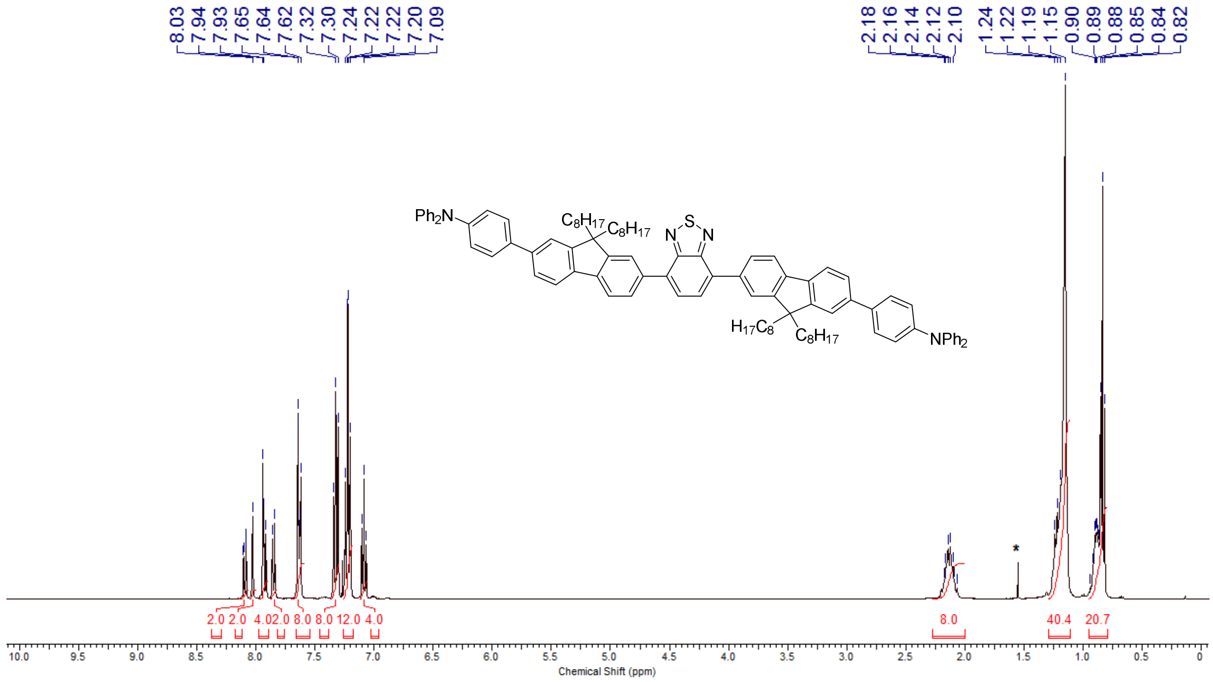

^{1}**H NMR** spectrum of ***11*** in $CDCl_3$. * = H_2O

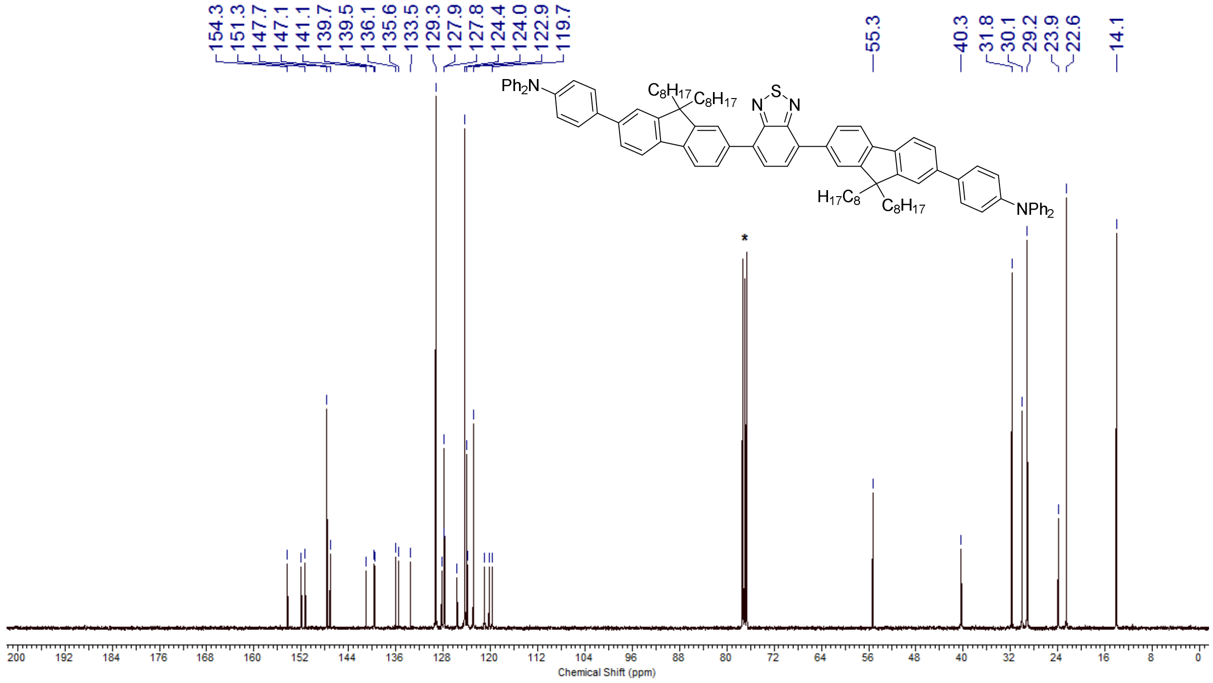

$^{13}C\{^{1}H\}$ NMR spectrum of ***11*** in $CDCl_3$. * = $CDCl_3$

Optical Properties

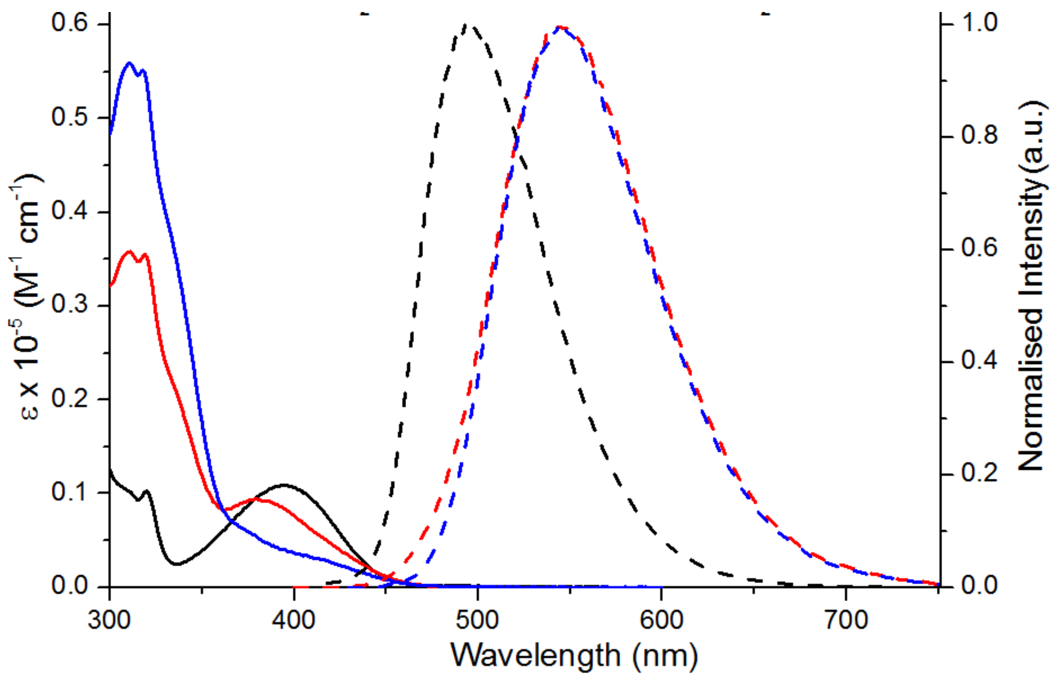

Figure S1: UV-vis absorbance (solid lines) and normalised photoluminescence spectra (dashed lines) of **1**, ***3*** and ***9*** in toluene (1 x 10^{-5} M).

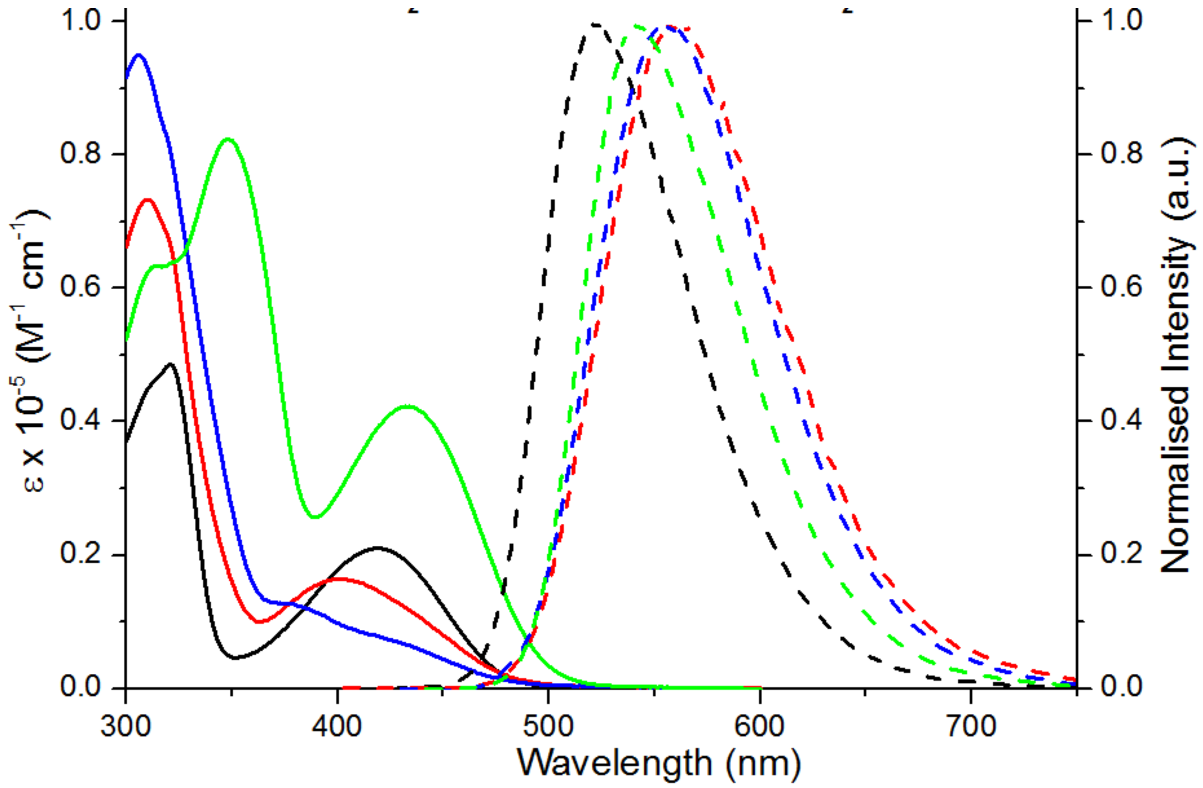

Figure S2: UV-vis absorbance (solid lines) and normalised photoluminescence spectra (dashed lines) of **2**, ***6***, ***10*** and ***11*** in toluene (1 x 10^{-5} M).

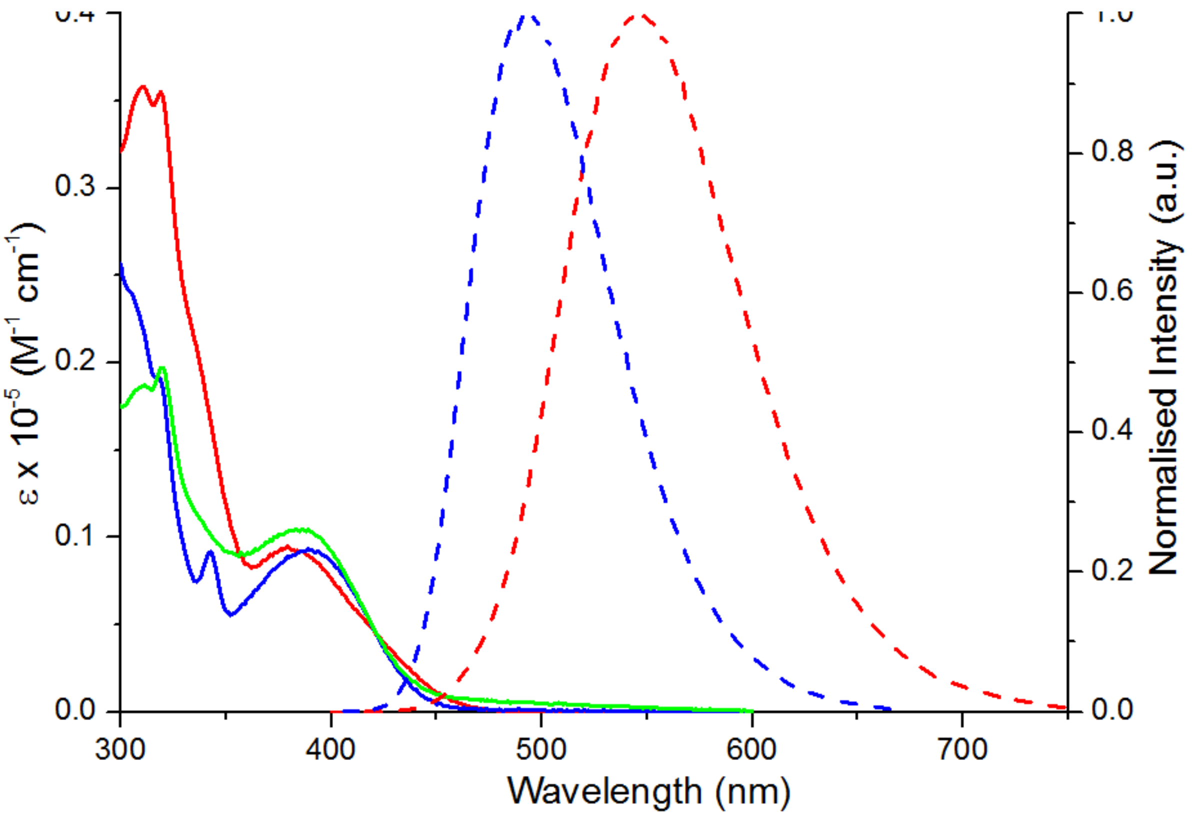

Figure S3: UV-vis absorbance (solid lines) and normalised photoluminescence spectra (dashed lines) of ***3***, ***4*** and ***5*** in toluene (1 x 10^{-5} M) (***5*** is not emissive).

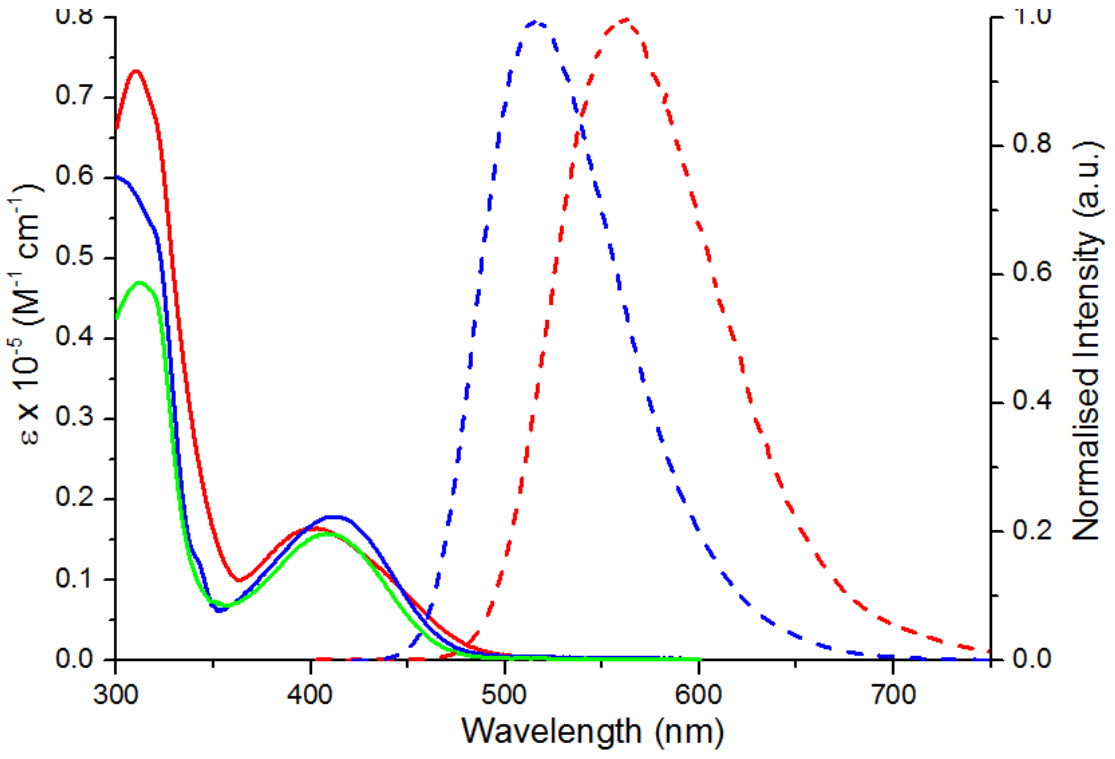

Figure S4: UV-vis absorbance (solid lines) and normalised photoluminescence spectra (dashed lines) of **6**, ***7*** and ***8*** in toluene (1 x 10^{-5} M) (***8*** is not emissive).

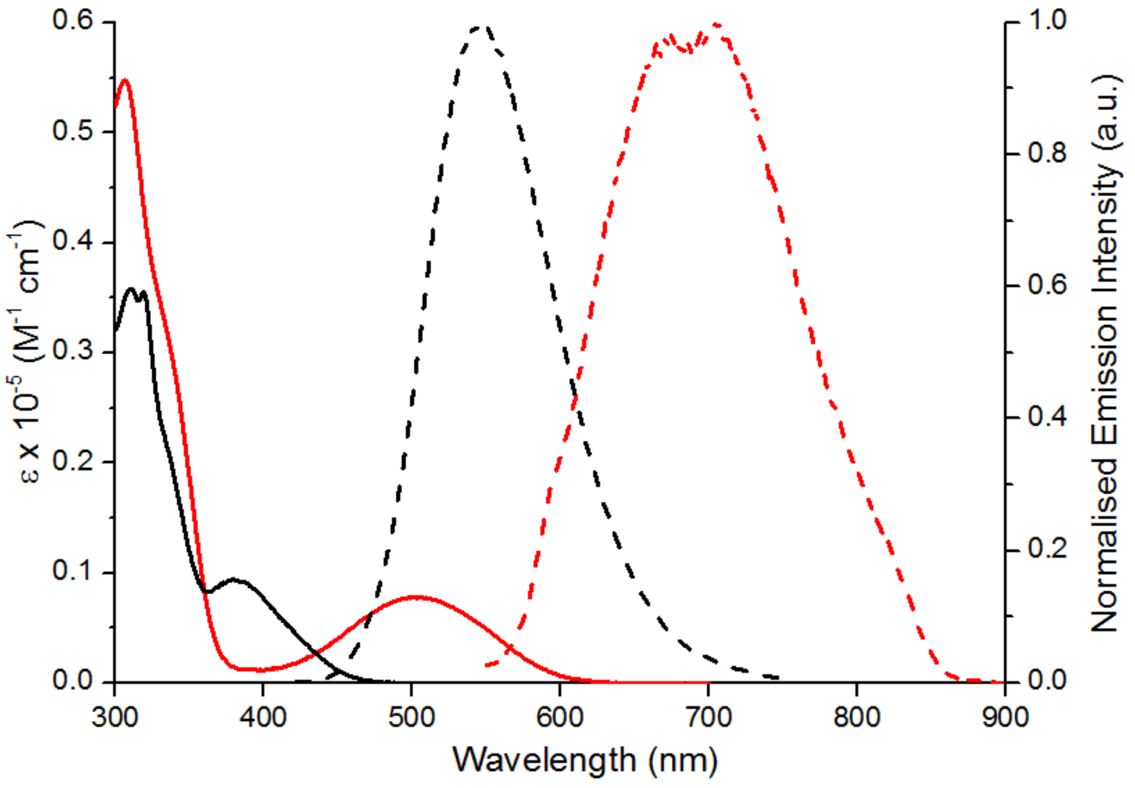

Figure S5: UV-vis absorbance (solid lines) and normalised photoluminescence spectra (dashed lines) of **3** and **3-BPh₂** in toluene (1 x 10^{-5} M).

Table S1: Summary of photophysical properties in toluene.

Compound	λ max_{abs} (nm)	ε x10^3 (M^{-1} cm^{-1})	λ max_{em} (nm)	Stokes shift (nm)	$Φ_f$ (%) (Air)	E_g^{opt} (eV)
**1**	319, 394	10.2, 10.9	493	99	- - -	2.77
3	311, 319, 379	35.8, 35.6, 9.5	546	167	9.9	2.72
**9**	311, 318, 368 (s), 400 (s)	55.9, 55.2, 6.8, 3.5	544	144	3.9	2.67
4	343, 389	9.2, 9.4	492	103	58.2	2.81
5	320, 384	19.7, 10.4	- - -	- - -	N/A	2.80
**3-BPh_2**	306, 502	54.8, 7.9	704	202	<1	2.07
**2**	321, 419	48.7, 21.0	521	102	- - -	2.59
6	310, 403	73.4, 16.4	564	161	42.0	2.57
**10**	306, 379, 420 (s)	95.0, 12.7, 7.8	554	134	7.9	2.56
**11**	348, 432	82.4, 47.3	542	110	69.0	2.50
7	412	17.9	516	104	75.7	2.69
8	312, 408	47.0, 15.8	- - -	- - -	N/A	2.68

Cyclic Voltammetry

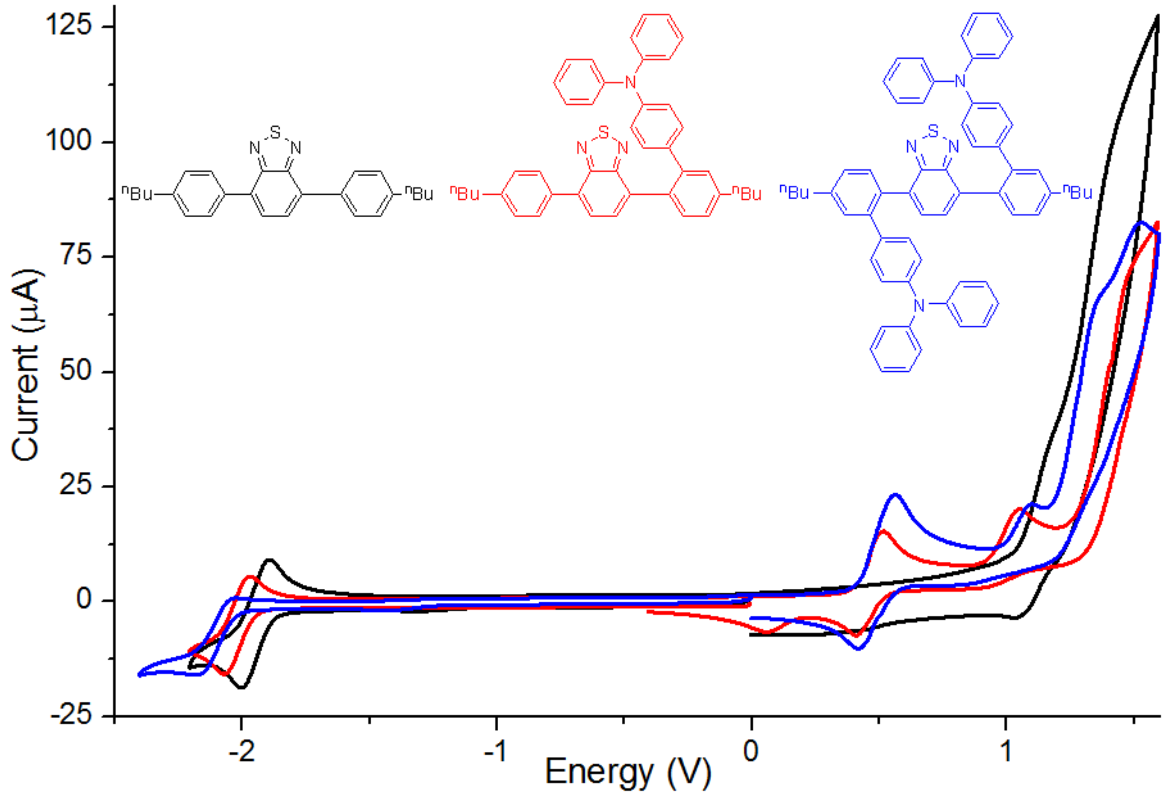

Figure S6: Cyclic voltammetry plots of **1**, ***3*** and ***9***, measured in DCM (1 mM) with [$^{n}Bu_4N$][PF_6] (0.1 M) as the supporting electrolyte at a scan rate of 50 mV s^{-1}.

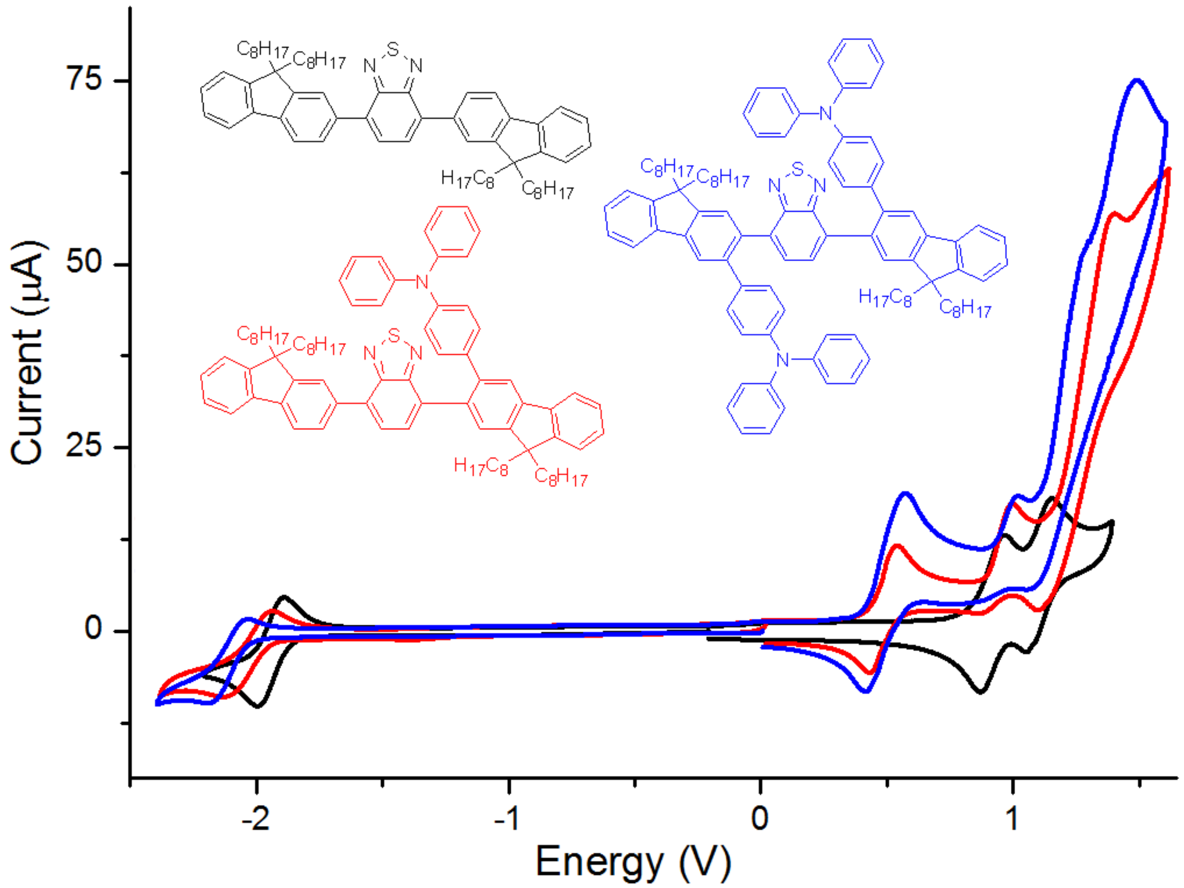

Figure S7: Cyclic voltammetry plots of **2**, ***6*** and ***10***, measured in DCM (1 mM) with [$^{n}Bu_4N$][PF_6] (0.1 M) as the supporting electrolyte at a scan rate of 50 mV s^{-1}.

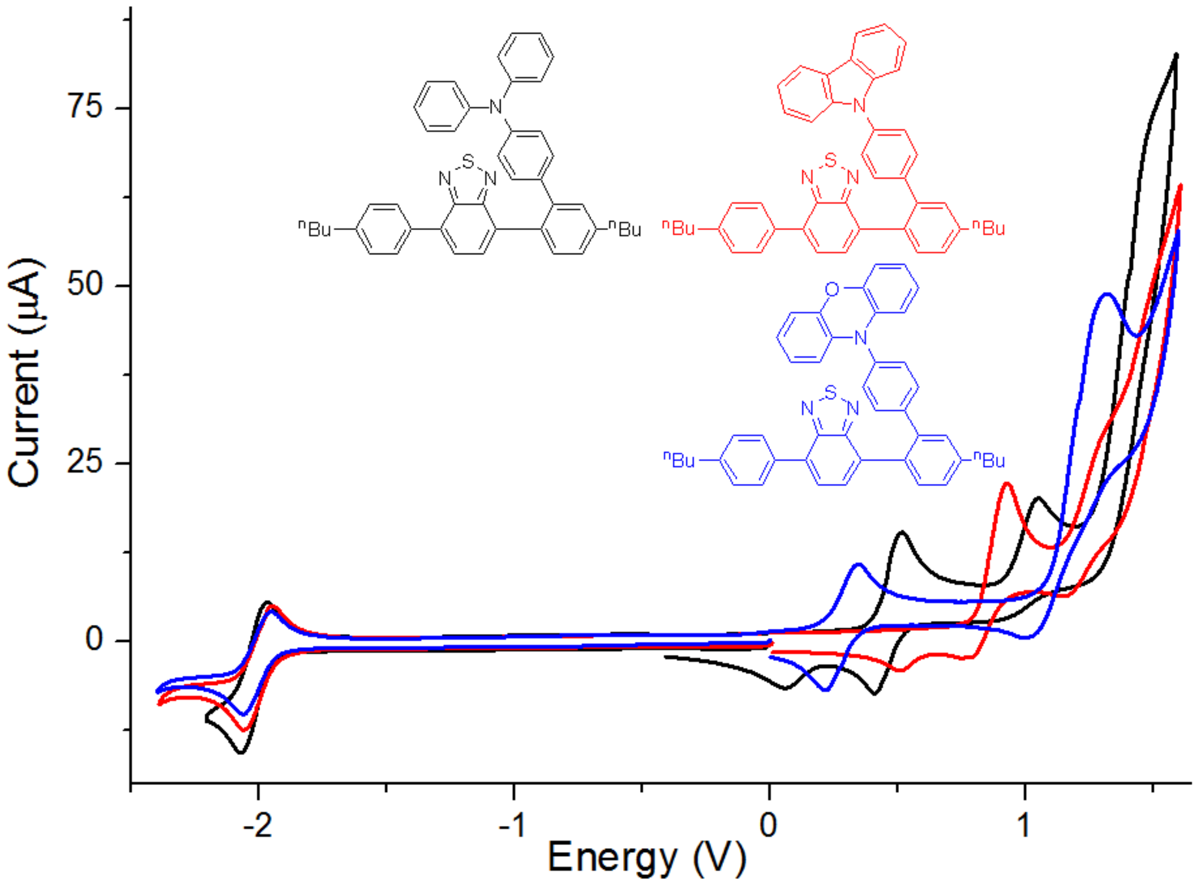

Figure S8: Cyclic voltammetry plots of ***3***, ***4*** and ***5***, measured in DCM (1 mM) with [^{n}Bu$_4$N][PF$_6$] (0.1 M) as the supporting electrolyte at a scan rate of 50 mV s^{-1}.

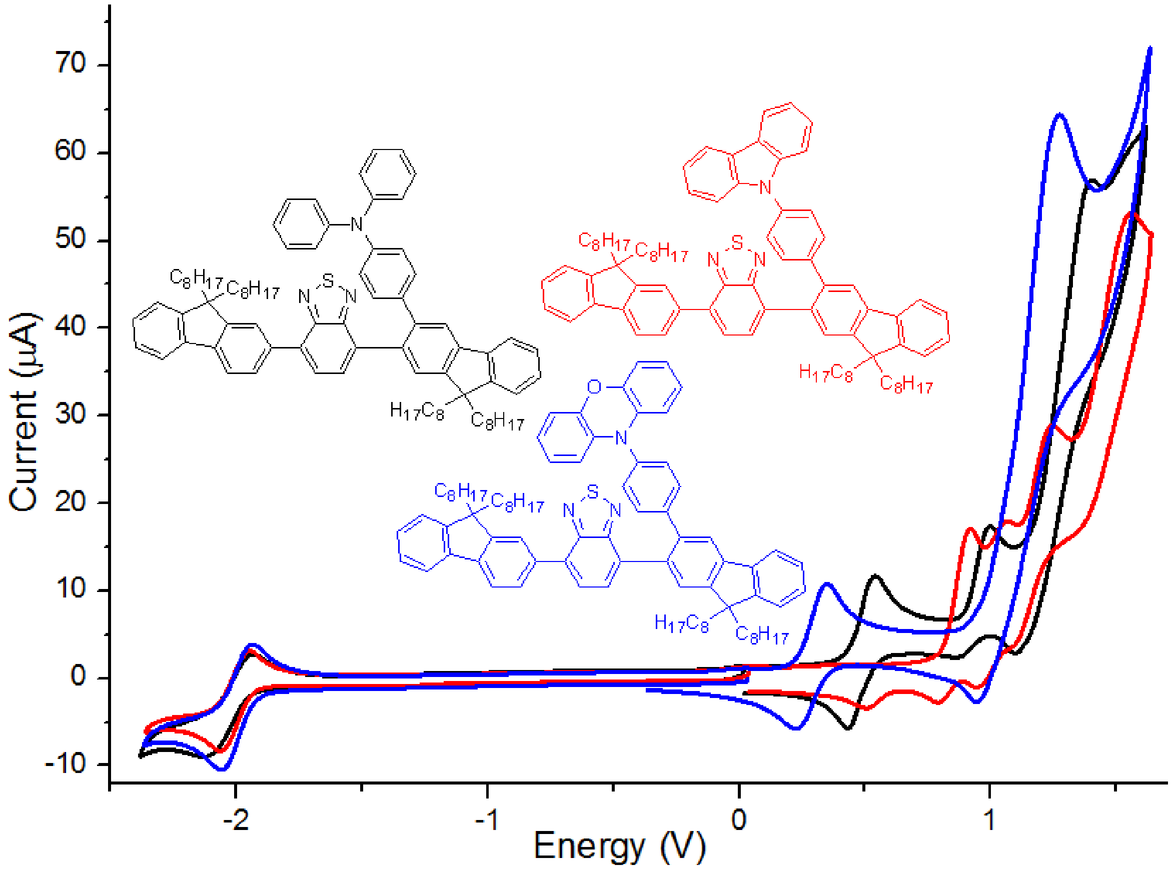

Figure S9: Cyclic voltammetry plots of ***6***, ***7*** and ***8***, measured in DCM (1 mM) with [$^{n}Bu_4N$][PF_6] (0.1 M) as the supporting electrolyte at a scan rate of 50 mV s^{-1}.

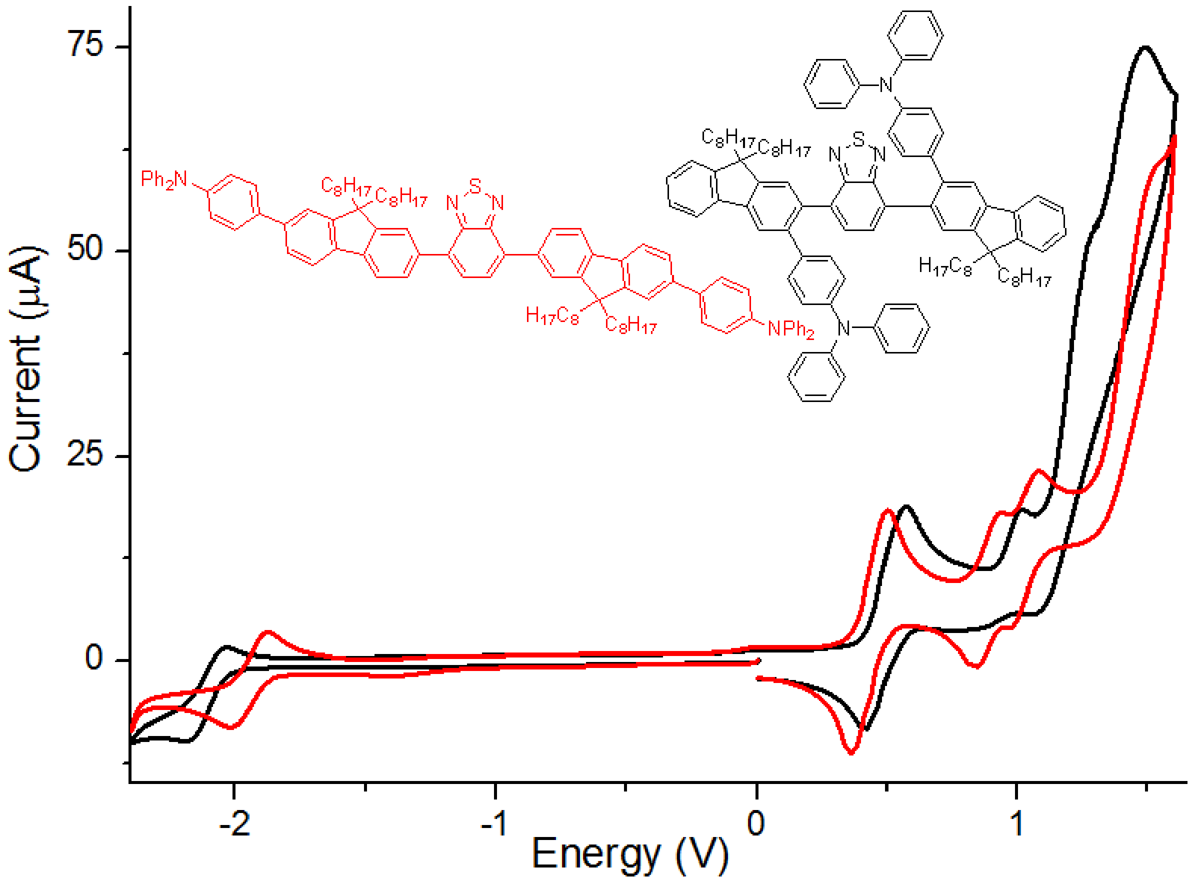

Figure S10: Cyclic voltammetry plots of ***10*** and ***11***, measured in DCM (1 mM) with [$^{n}Bu_4N$][PF_6] (0.1 M) as the supporting electrolyte at a scan rate of 50 mV s^{-1}.

Table S2: Comparison of redox properties.

Compound	E_{ox}^{onset} (V)	E_{red}^{onset} (V)	HOMO (eV)	LUMO (eV)	E_g^{EC} (eV)
**1**	1.05	-1.89	-6.15	-3.21	2.94
3	0.42	-1.95	-5.52	-3.15	2.37
**9**	0.42	-2.03	-5.52	-3.07	2.45
**2**	0.88	-1.87	-5.98	-3.23	2.75
6	0.42	-1.95	-5.52	-3.15	2.37
**10**	0.42	-2.04	-5.52	-3.06	2.46
4	0.81	-1.93	-5.91	-3.17	2.74
5	0.21	-1.93	-5.31	-3.17	2.14
**11**	0.37	-1.88	-5.47	-3.22	2.25
7	0.81	-1.94	-5.91	3.16	2.75
8	0.22	-1.93	-5.32	-3.17	2.15

Measured in DCM (1 mM) with [$^{n}Bu_4N$][PF_6] (0.1 M) as the supporting electrolyte at a scan rate of 50 mV s^{-1}. Potentials are given relative to the Fc/Fc+ redox couple which is taken to be 5.1 eV below vacuum.

Delayed Emission Studies

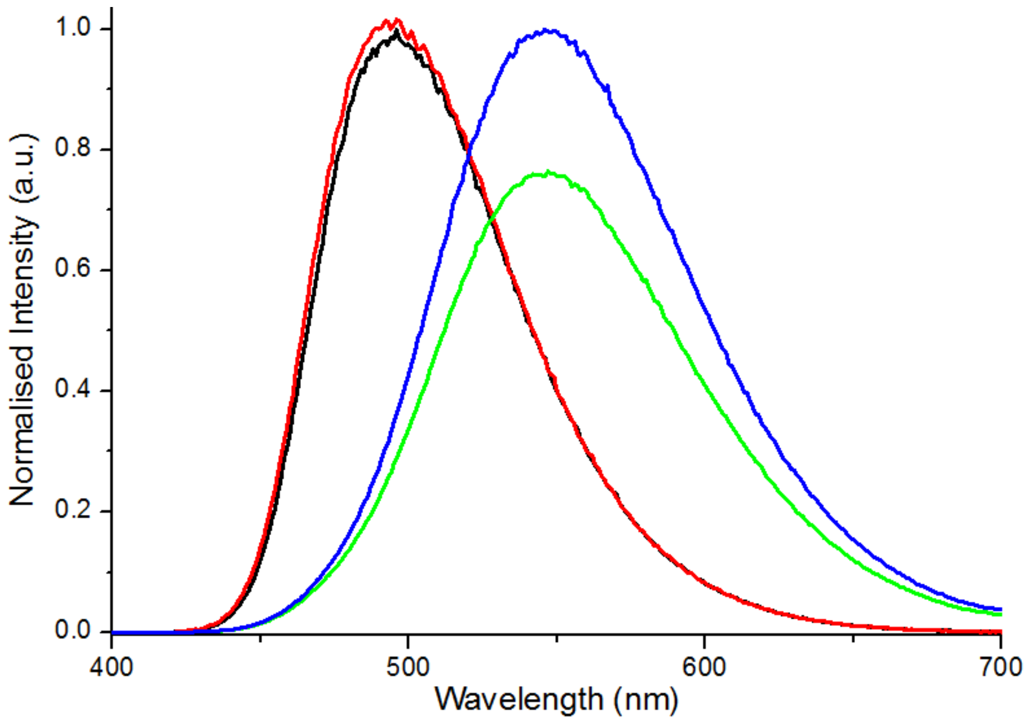

Figure S11: Normalised photoluminescence spectra in (toluene) of **1 in Air**, of **1 under Argon** *(sample prepared in a glovebox in a sealed cuvette using degassed solvent),* of **3 in Air, and 3 under Argon** *(sample prepared in a glovebox in a sealed cuvette using degassed solvent)*.

Compound **3** showed a decrease in the fluorescence intensity by ~23 % after the addition of air (Figure S11).

Emission is observed at delay times after excitation of >0.1 ms with decreasing intensity as the delay time increases. The spectra are essentially identical to that of the prompt fluorescence. Additionally, complete quenching of the delayed fluorescence is observed under Air saturated conditions. Therefore, the origin of the delayed emission is assigned to reverse intersystem crossing from the triplet state (Figure S12).

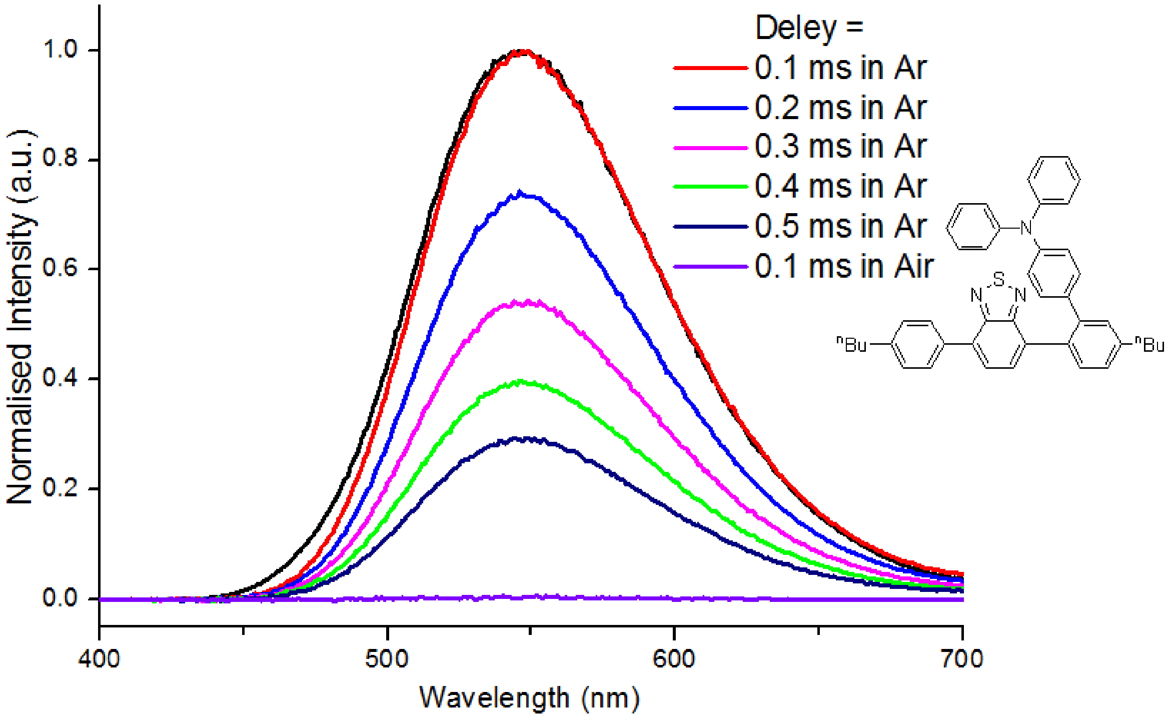

Figure S12: Normalised emission intensity of compound **3** at different delay times under Argon (Ar) and after 0.1 ms under Air.

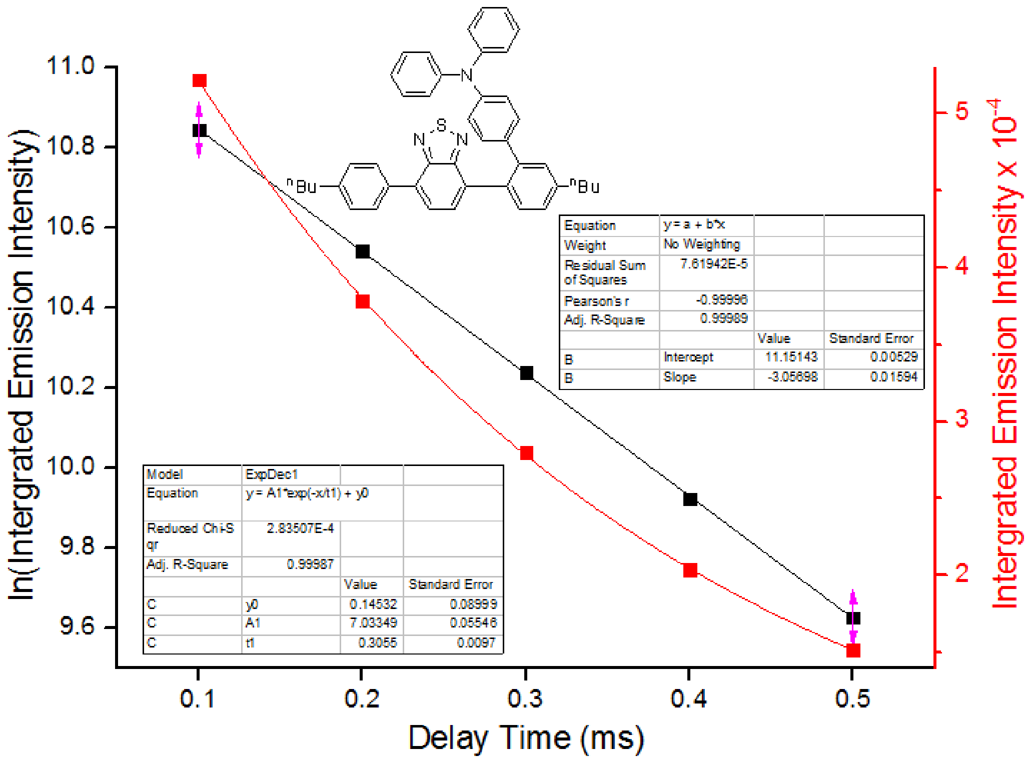

Figure S13: Compound **3** shows an exponential decrease in emission intensity with increasing delay time.

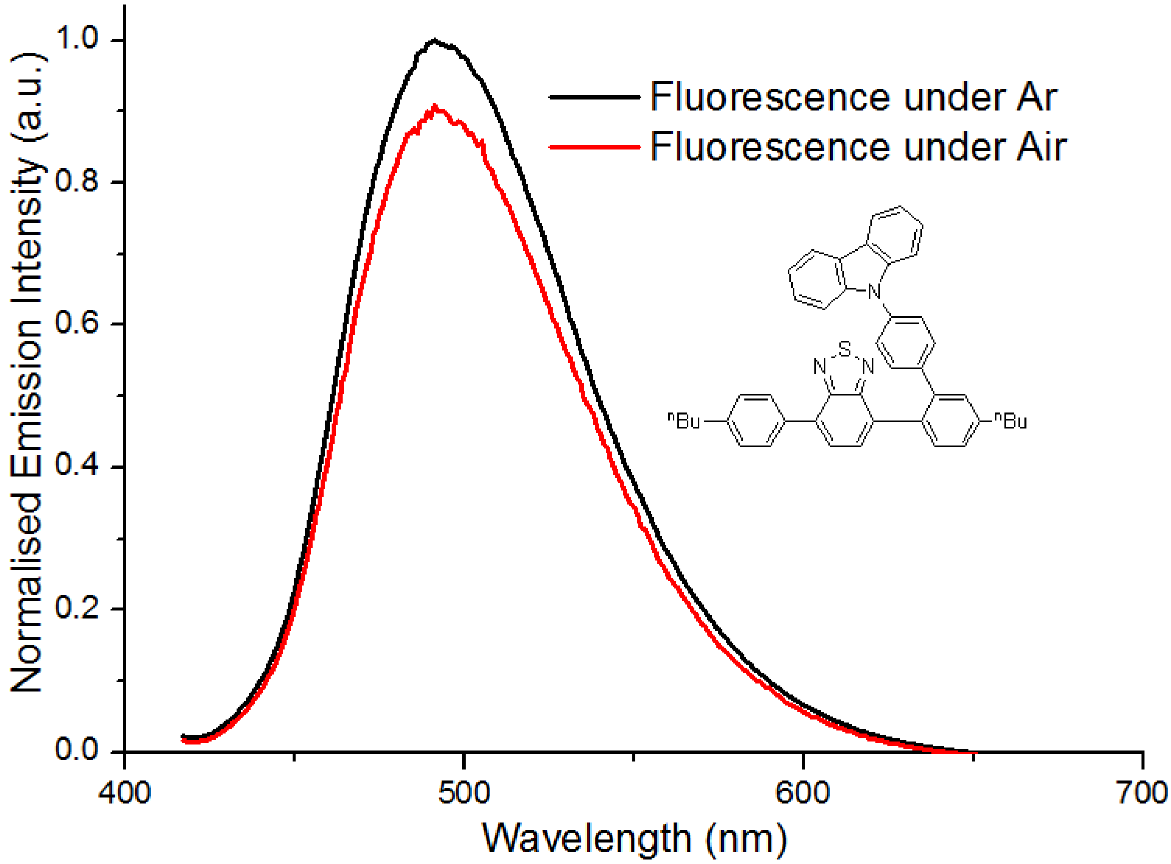

Figure S14: Normalised photoluminescence spectra in (toluene) of **4 in Air**, of **4 under Argon** *(sample prepared in a glovebox in a sealed cuvette using degassed solvent)*.

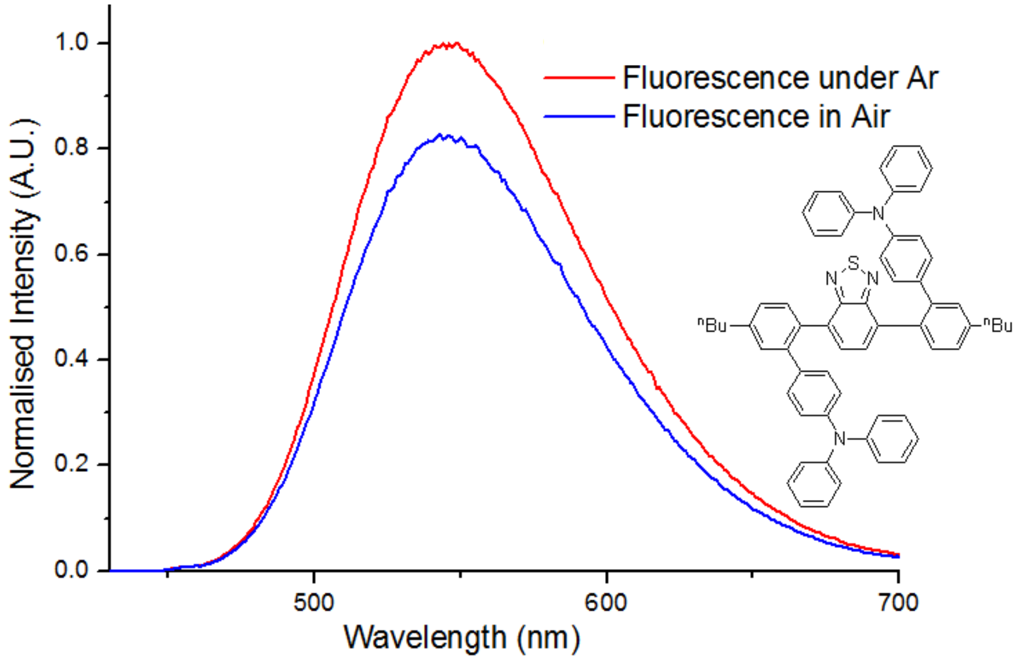

Figure S15: Normalised photoluminescence spectra in (toluene) of **9 under Air**, of **9 under Argon** *(sample prepared in a glovebox in a sealed cuvette using degassed solvent)*. Compound **9** showed a decrease in the fluorescence intensity by ~17 % after addition of air

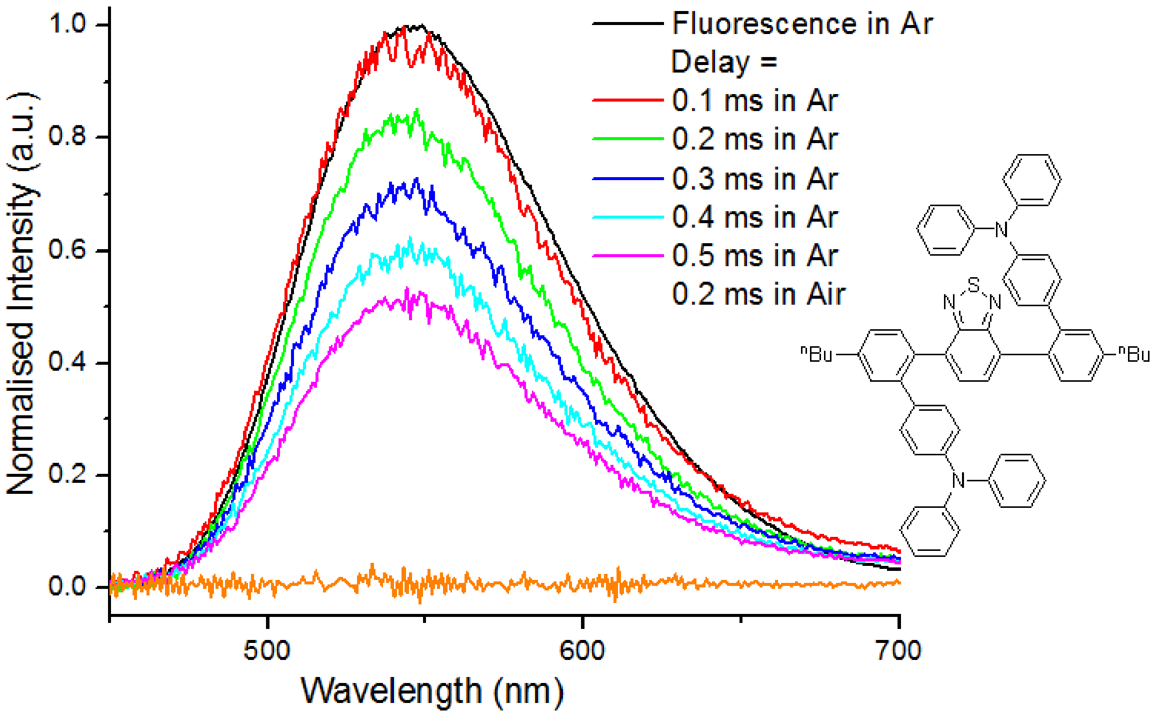

Figure S16: Normalised emission intensity of compound **9** at different delay times under Argon (Ar) and after 0.1 ms under Air. Emission is observed at delay times of >0.1 ms with decreasing intensity as the delay time increases. The spectra are essentially identical to that of the prompt fluorescence. Additionally, complete quenching of the delayed fluorescence is observed under Air saturated conditions. (Figure S16).

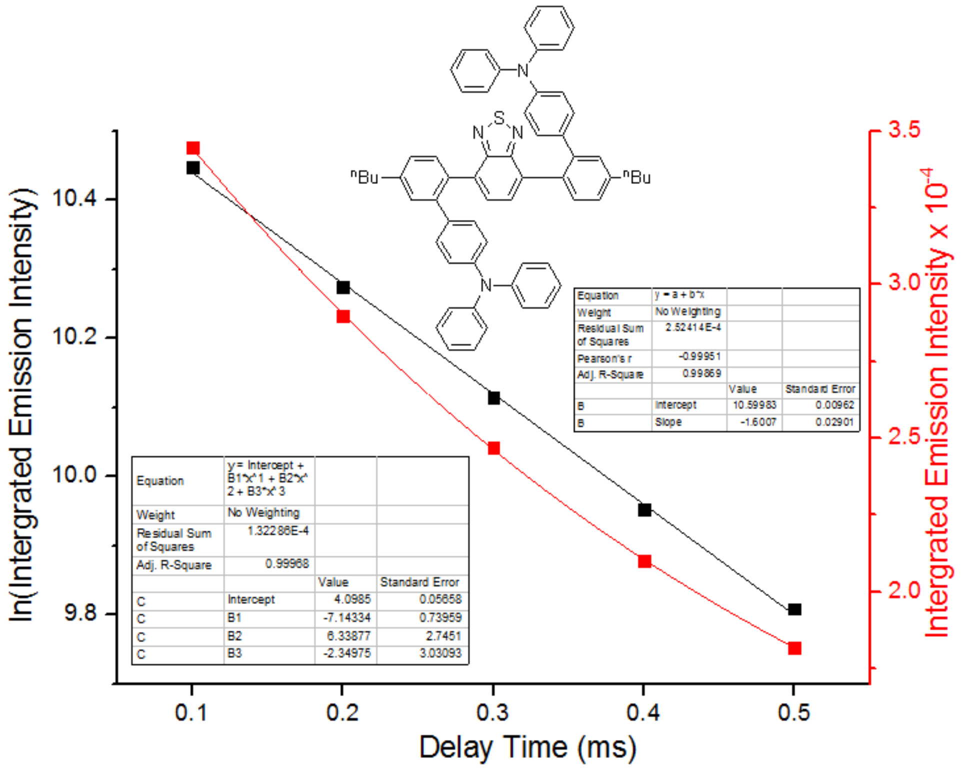

Figure S17: **9** shows an exponential decrease in emission intensity with increasing delay time

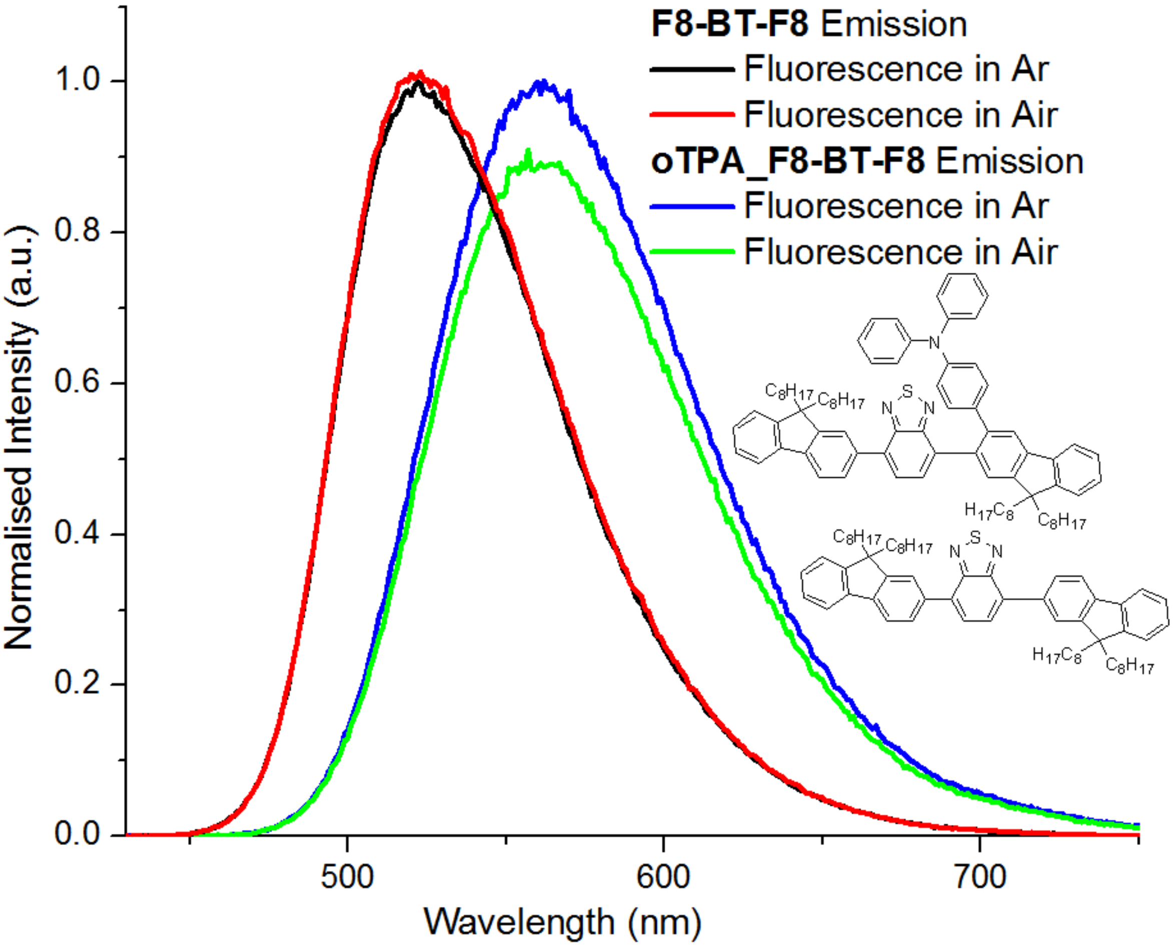

Figure S18: Normalised photoluminescence spectra in (toluene) of **2 in Air**, of **2 under Argon** *(sample prepared in a glovebox in a sealed cuvette using degassed solvent),* of **6 in Air**, **and 6 under Argon** *(sample prepared in a glovebox in a sealed cuvette using degassed solvent).* Compound **6** showed a decrease in the fluorescence intensity by ~10 % after the addition of O_2 (Figure S18). Compound **6** in dilute toluene solution demonstrates a quantum yield value of 46.7 % under argon which reduces to 42.0 % under air (Figure S19).

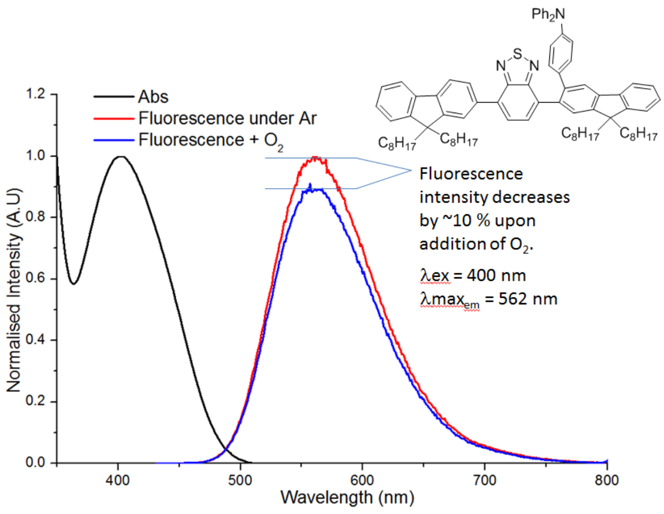

Figure S19: Normalised emission intensity and absorbance of compound **6** in toluene under the argon and air.

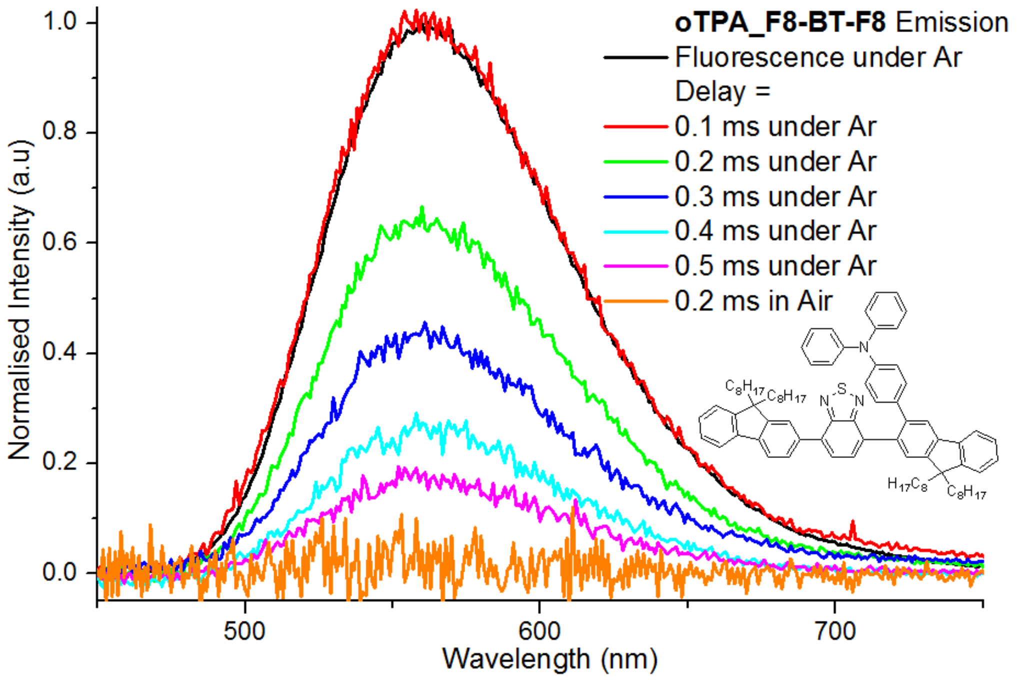

Figure S20: Normalised emission intensity of compound **6** at different delay times

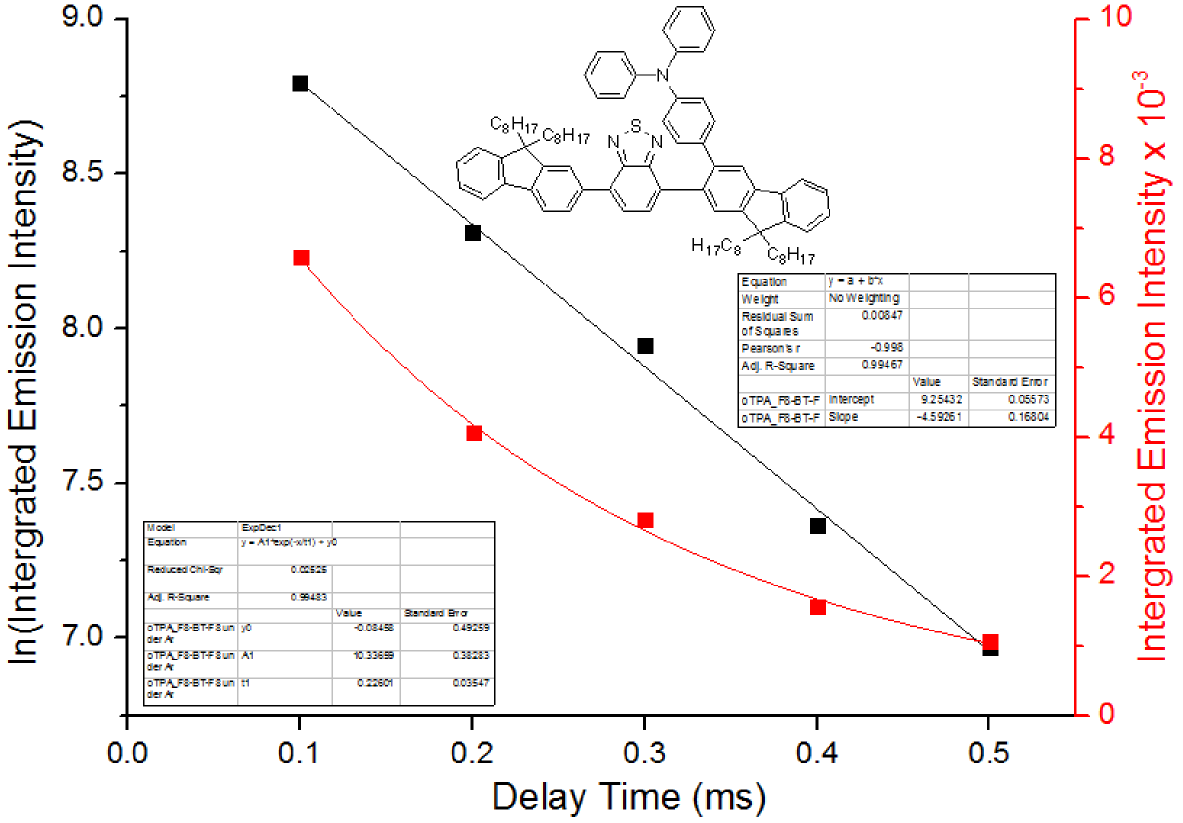

Figure S21: Exponential decay of emission intensity of compound **6.**

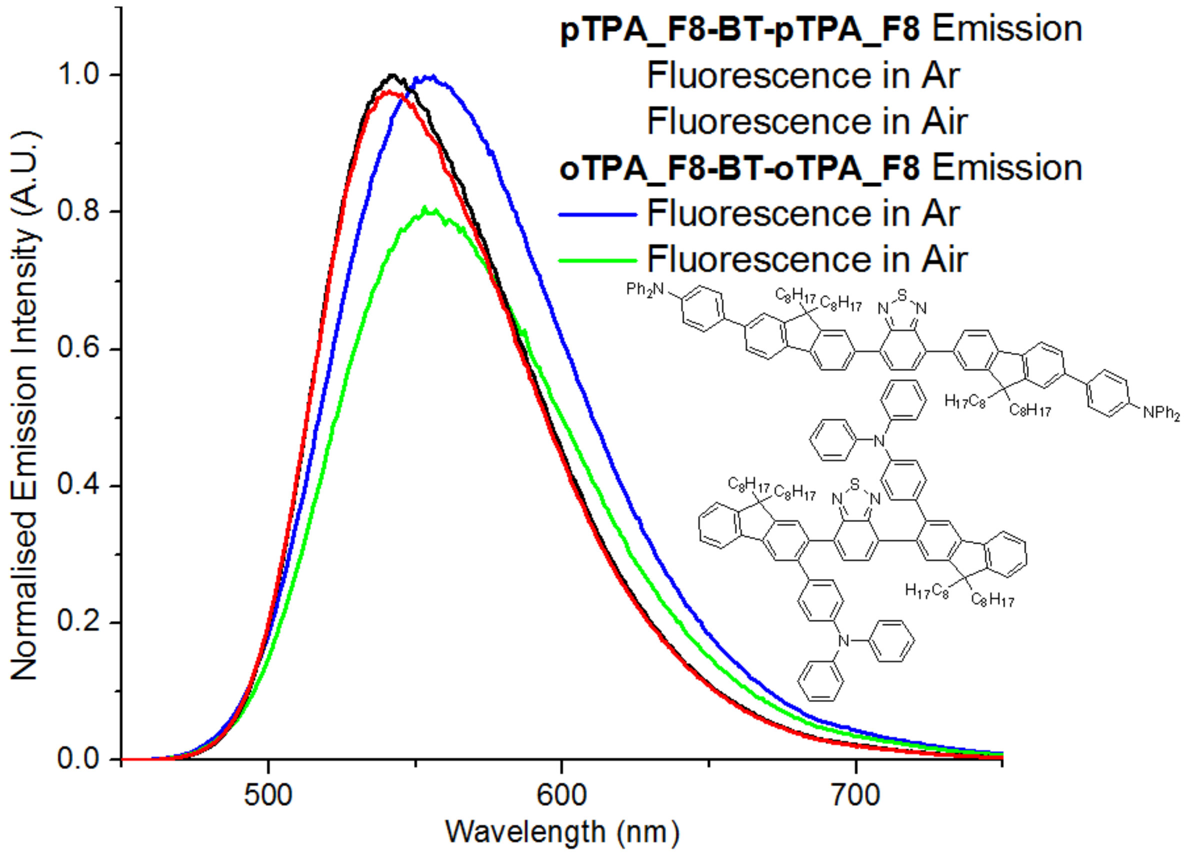

Figure S21: Normalised photoluminescence spectra in (toluene) of **11 in Air**, of **11 under Argon** *(sample prepared in a glovebox in a sealed cuvette using degassed solvent),* of **10 in Air**, **and 10 under Argon** *(sample prepared in a glovebox in a sealed cuvette using degassed solvent)*.

Compound **10** showed a decrease in the fluorescence intensity by ~20 % after the addition of O_2 (Figure S21). Emission is observed at delay times of >0.1 ms with decreasing intensity as the delay time increases. The spectra are essentially identical to that of the prompt fluorescence. Additionally, complete quenching of the delayed fluorescence is observed under Air saturated conditions. Therefore, the origin of the delayed emission is assigned to reverse intersystem crossing from the triplet state (Figure S22).

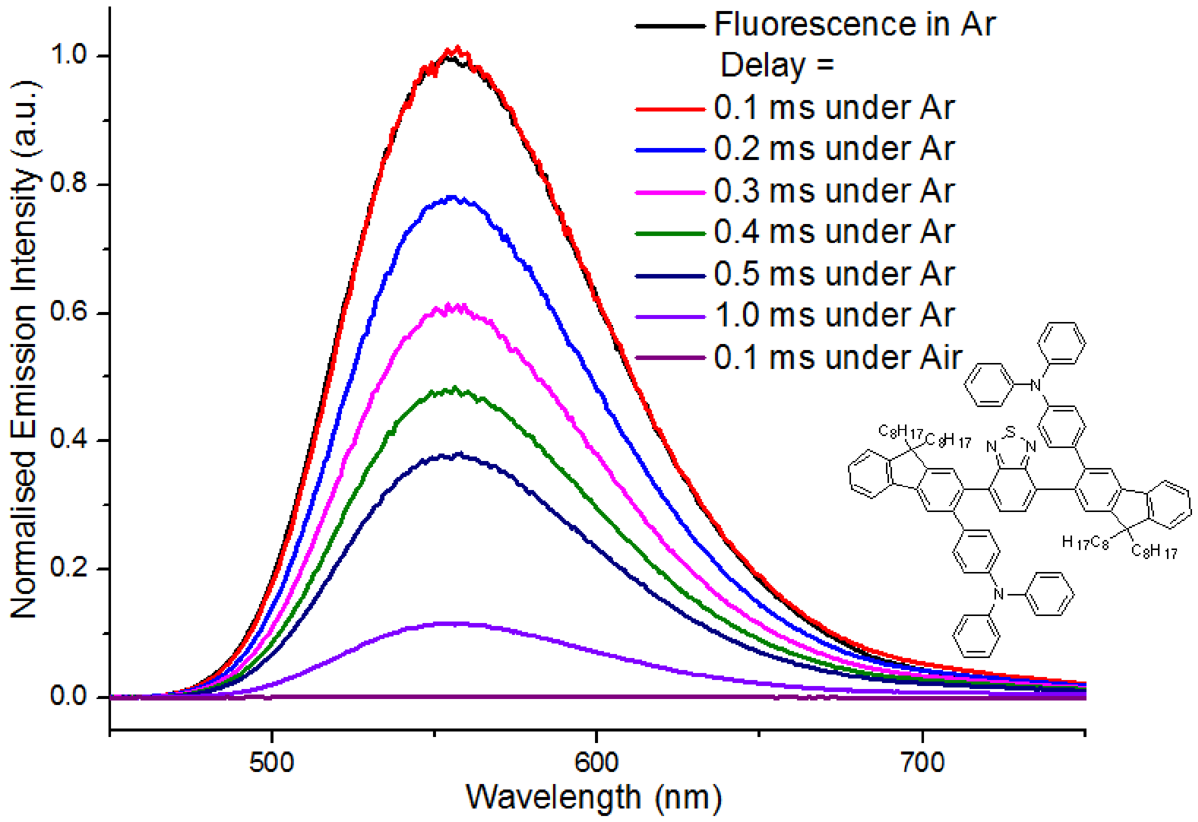

Figure S22: Normalised emission intensity of compound **10** at different delay times under Argon (Ar) and after 0.1 ms under Air. Emission is observed at delay times of >0.1 ms with decreasing intensity as the delay time increases. The spectra are essentially identical to that of the prompt fluorescence.

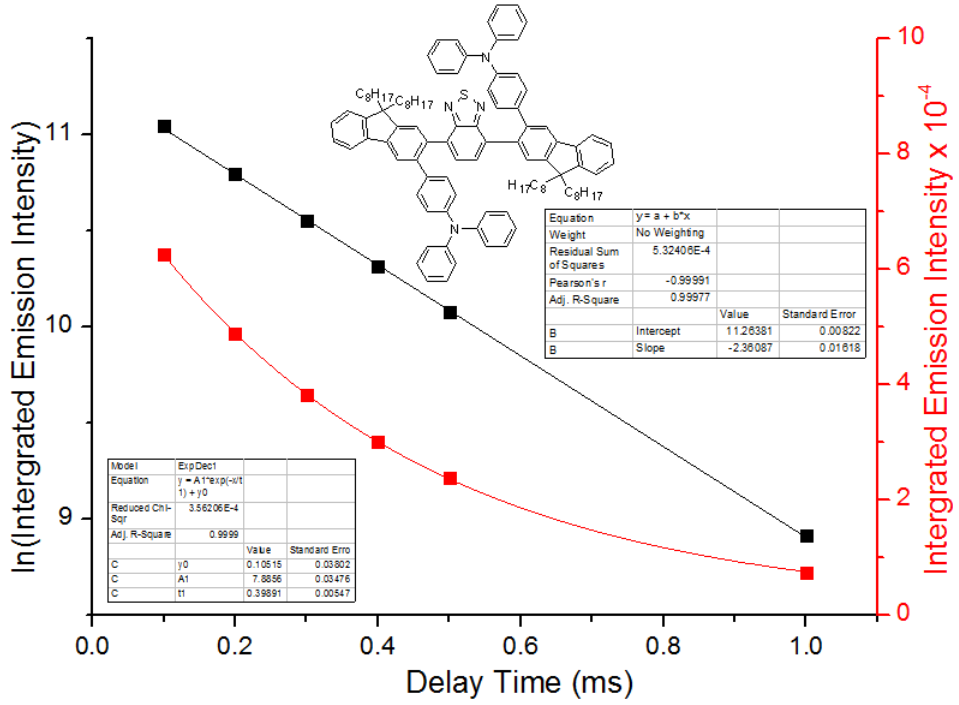

Figure S23: An exponential decrease in emission intensity with increasing delay time for **10**

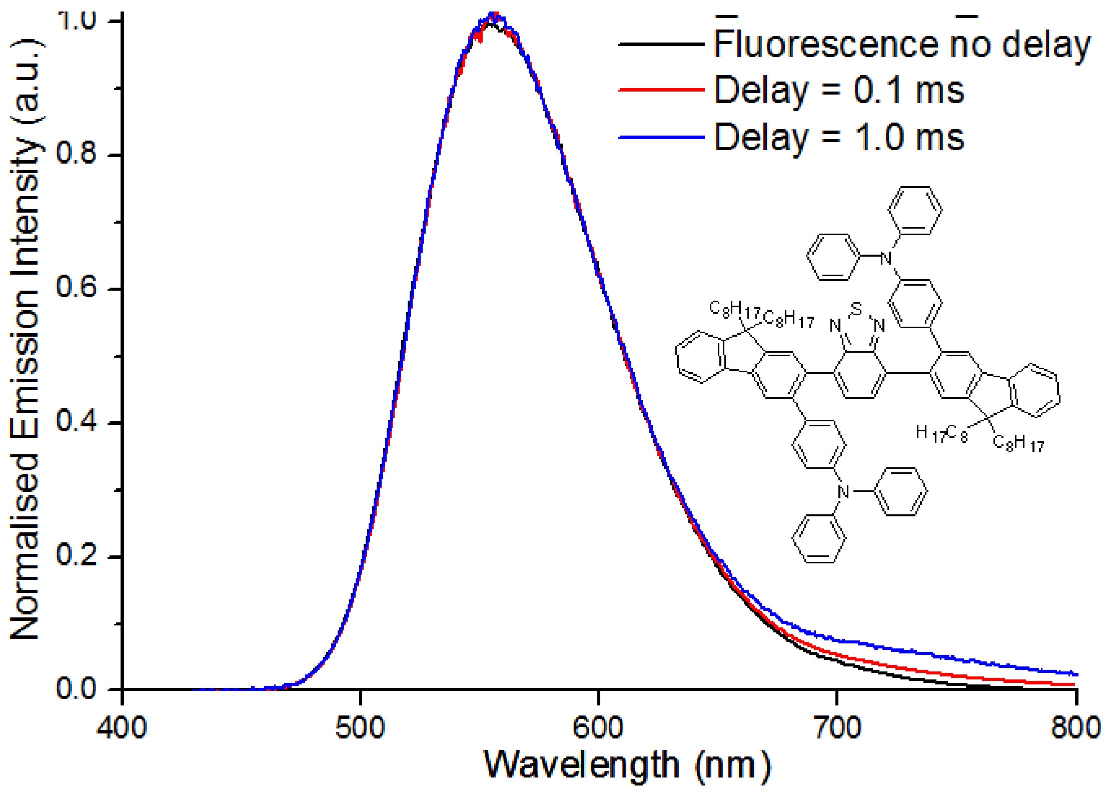

Figure S24: The emission spectra of compound **10** (under Argon) remains essentially identical at delay times up to 1 ms (Figure 17).

The effects of the triphenylamine unit ortho- to the benzothiadiazole unit can be observed as the linear 7,7' substituted isomer of **10** (compound **11**) shows no appreciable decrease in emission intensity upon the addition of O_2 and no delayed emission is observed (Figure 25).

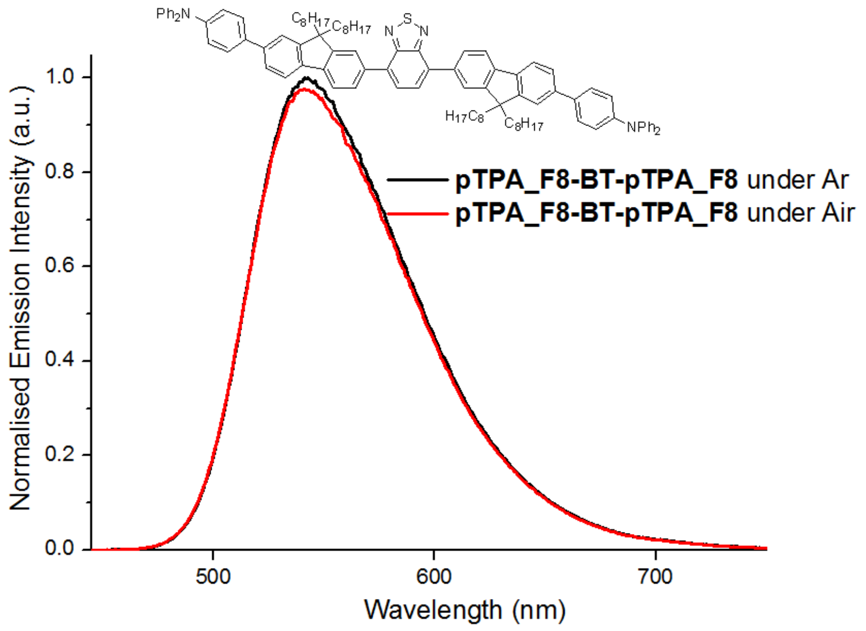

Figure S25: Normalised emission intensity of **11** in toluene under the argon and air.

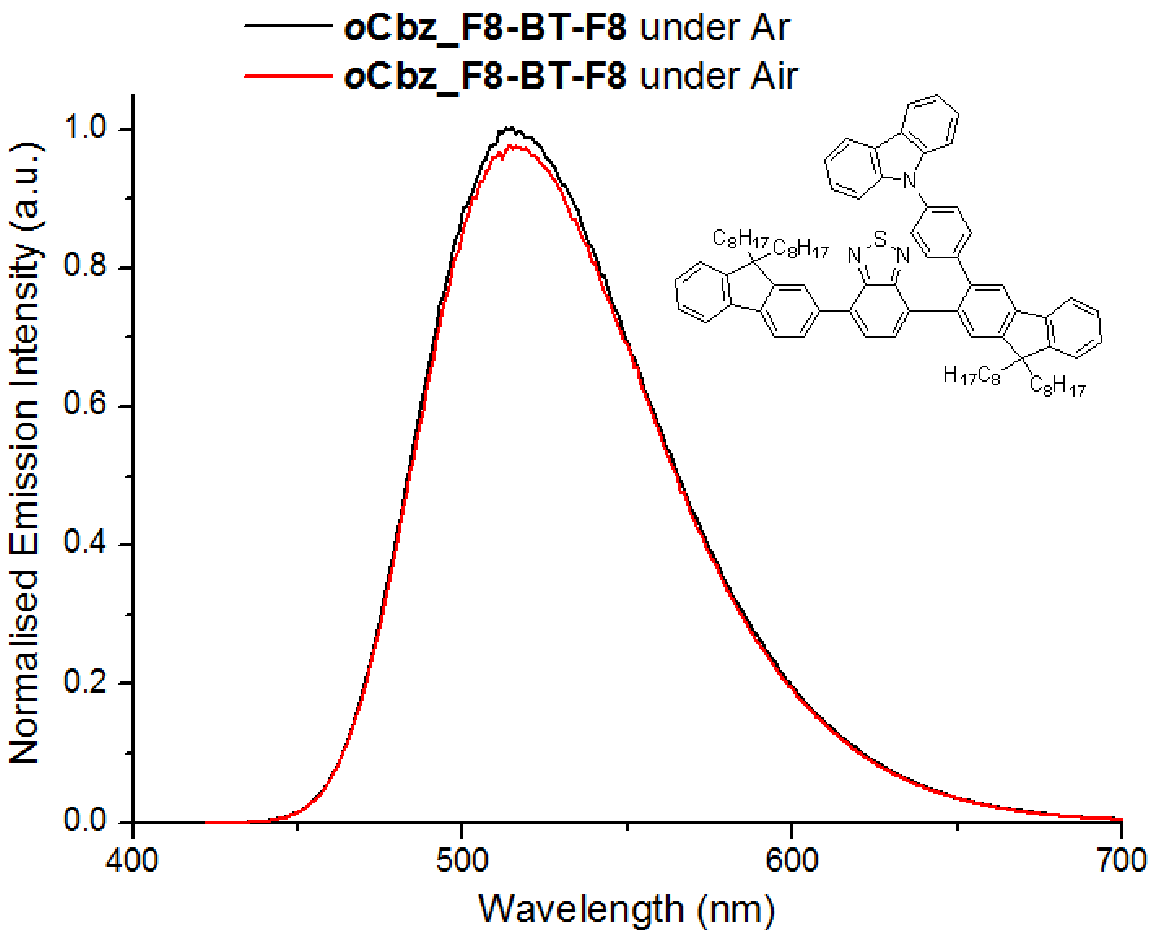

Figure S25: Normalised emission intensity of **7** in toluene under the argon and air.

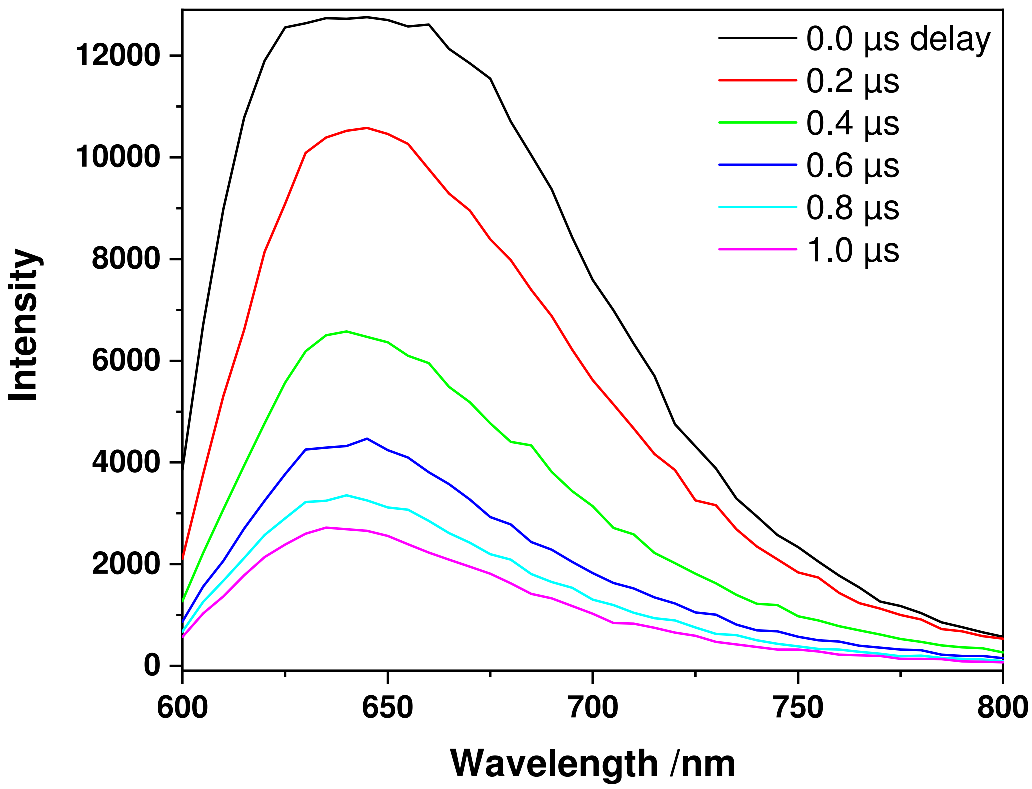

Figure S26: Solid state emission of crystalline **3-BPh₂** run under air with varying delay times after excitation.

For comparison compound **DPS-PXZ,** which is a well-studied thermally activated delayed fluorescence (TADF) material published by Adachi et. al.,[S10] was synthsized using the published procedure and in dilute toluene solution under an argon atmosphere **DPS-PXZ** shows intense fluorescence and weak emission after a 0.2 ms delay. Upon the addition of oxygen a significant decrease in the fluorescence intensity by ~70% (reported 73% decrease in quantum yield upon addition of O_2)[1] is observed and the delayed emission is absent (Figure S27).

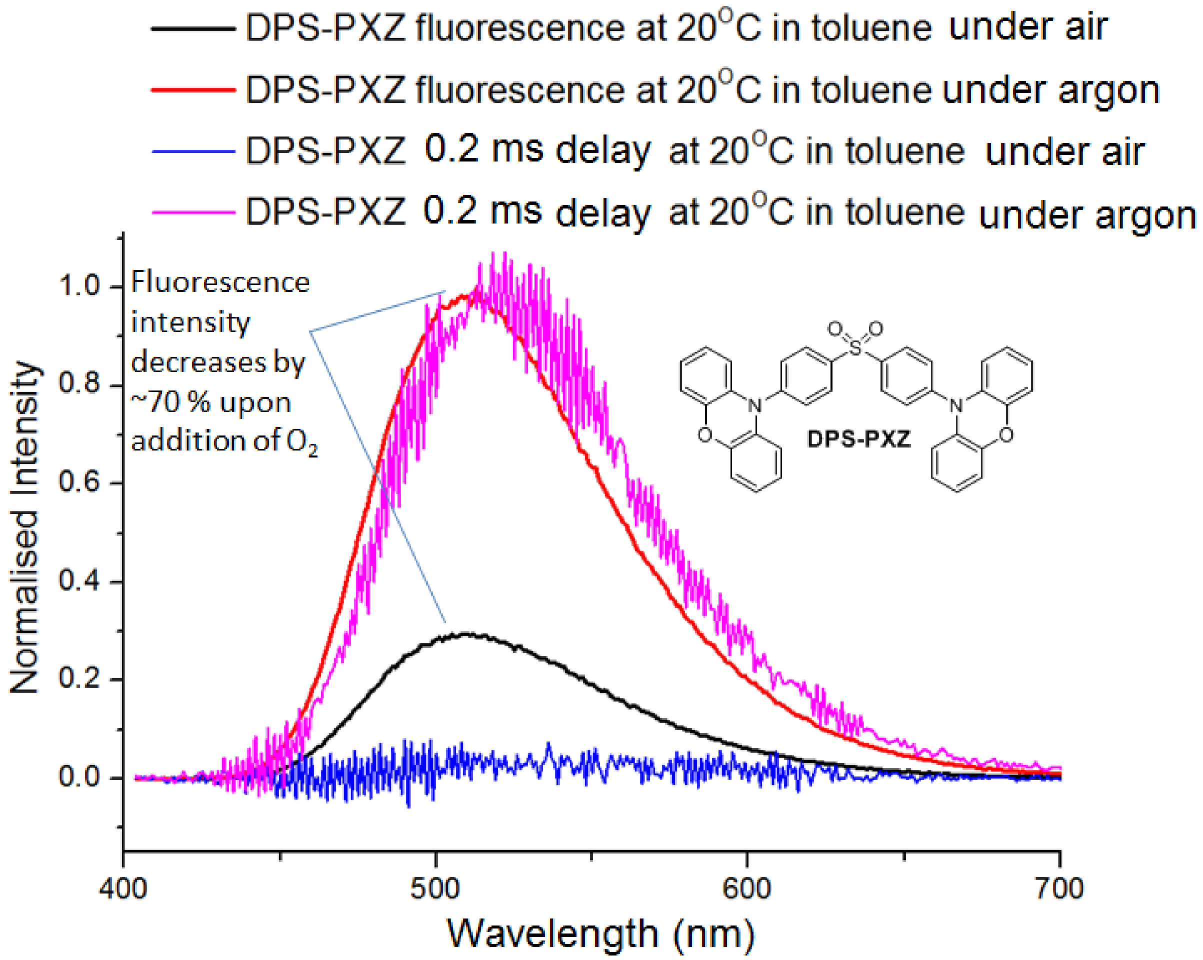

Figure S27: Emission of DPS-PXZ in toluene under argon and air.

A related compound **DPS-TCb** published by Monkman et. al.,S11 shows emission at delay times up to 600 μs with decreasing intensity as the delay time increases. The spectra are essentially identical to that of the prompt fluorescence. Additionally, complete quenching of the delayed fluorescence is observed under Air saturated conditions (Figure S28).

These observations on two other compounds are closely comparable to the photophysical properties observed for the triphenylamine functionalised compounds studied in this work.

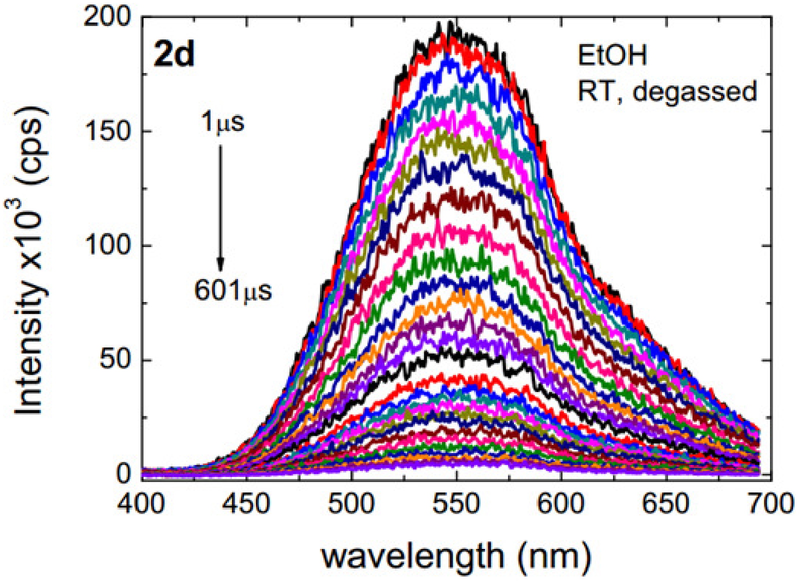

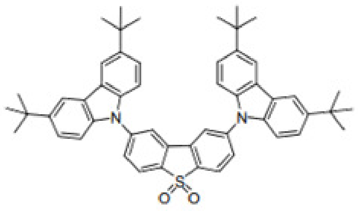

DPS-TCb

Figure S28: The delayed fluorescence emission in **DPS-TCb** decays within 600 μs with a single exponential time constant. Taken from reference S11

Crystallographic Details

Crystallographic data for compound **3-BPh$_2$** were recorded on an Agilent Supernova diffractometer, at 150 K with Mo Kα radiation (mirror monochromator, λ =0.7107). The CrysAlisPro software package was used for data collection, cell refinement and data reduction.[S12] Data for compounds **3** and **4** were collected on an Oxford Diffraction Xcalibur 2 at 150 and 293 K, respectively, with Mo Kα radiation (mirror monochromator, λ =0.7107). Synchotron X-ray data for compound **5** were collected at beamline I19 (λ = 0.6889 Å) of Diamond Light Source at a temperature of 100 K and measured using GDA suite of programs.[S13] The CrysAlisPro5 (**3**, **3-BPh$_2$**, **4**) and dials (**5**) software packages were used for data collection, cell refinement and data reduction. For all data sets the CrysAlisPro software package was used for empirical absorption corrections, which were applied using spherical harmonics, implemented in SCALE3 ABSPACK scaling algorithm. All further data processing was undertaking within the Olex2 software.[S14] The molecular structures all compounds were solved with ShelXT[S15] structure solution program using Intrinsic Phasing. All structures were refined with the SHELXL[S16] refinement package using Least Squares minimisation against F2. Non-hydrogen atoms were refined anisotropically. Hydrogen atoms were all located in a difference map and repositioned geometrically.

Table S3

Compound	3	3-BPh_2
CCDC code	1836435	1836436
Empirical formula	$C_{44}H_{43}N_3S$	$C_{56}H_{51}BN_3S$
Formula weight	645.87	808.86
Temperature/K	150(2)	150.02(10)
Crystal system	triclinic	triclinic
Space group	P-1	P-1
a/Å	9.2247(3)	11.4572(5)
b/Å	11.3106(5)	16.0336(12)
c/Å	18.2809(9)	25.0170(18)
α/°	72.934(4)	81.684(6)
β/°	89.017(4)	82.092(5)
γ/°	79.183(3)	78.143(5)
Volume/$Å^3$	1789.53(14)	4422.6(5)
Z	2	4
$\rho_{calc}g/cm^3$	1.199	1.215
μ/mm^{-1}	0.126	0.115
F(000)	688.0	1716.0
Crystal size/mm^3	1 × 0.5 × 0.5	0.4 × 0.27 × 0.13
Radiation	MoKα (λ = 0.71073)	MoKα (λ = 0.71073)
2Θ range for data collection/°	6.864 to 58.308	6.626 to 50.7
Index ranges	-12 ≤ h ≤ 12, -12 ≤ k ≤ 14, -21 ≤ l ≤ 24	-13 ≤ h ≤ 13, -19 ≤ k ≤ 19, -30 ≤ l ≤ 29
Reflections collected	15321	31917
Independent reflections	8097 [R_{int} = 0.0322, R_{sigma} = 0.0731]	16152 [R_{int} = 0.0601, R_{sigma} = 0.1172]
Data/restraints/parameters	8097/39/463	16152/346/1189
Goodness-of-fit on F^2	0.947	1.088
Final R indexes [I>=2σ (I)]	R_1 = 0.0803, wR_2 = 0.2168	R_1 = 0.0990, wR_2 = 0.2125
Final R indexes [all data]	R_1 = 0.1457, wR_2 = 0.2827	R_1 = 0.1557, wR_2 = 0.2426
Largest diff. peak/hole / e $Å^{-3}$	0.76/-0.51	0.56/-0.50

Compound	4	5
CCDC code	1836437	1836439
Empirical formula	$C_{44}H_{41}N_3S$	$C_{44}H_{41}N_3OS$
Formula weight	643.86	659.86
Temperature/K	293(2)	100(2)
Crystal system	monoclinic	triclinic
Space group	$P2_1/c$	P-1
a/Å	12.2226(13)	10.1468(6)
b/Å	7.2731(6)	12.5298(5)
c/Å	39.701(6)	15.0883(8)
α/°	90	99.960(4)
β/°	90.766(12)	94.002(5)
γ/°	90	107.912(4)
Volume/$Å^3$	3529.0(7)	1782.26(17)
Z	4	2
$\rho_{calc}g/cm^3$	1.212	1.230
μ/mm^{-1}	0.127	0.126
F(000)	1368.0	700.0
Crystal size/mm^3	0.5 × 0.3 × 0.1	0.07 × 0.06 × 0.03
Radiation	MoKα (λ = 0.71073)	? (λ = 0.6889)
2Θ range for data collection/°	6.518 to 59.02	3.382 to 51.006
Index ranges	-16 ≤ h ≤ 16, -9 ≤ k ≤ 9, -51 ≤ l ≤ 51	-12 ≤ h ≤ 12, -15 ≤ k ≤ 15, -18 ≤ l ≤ 18
Reflections collected	9294	20402
Independent reflections	9294 [R_{int} = ?, R_{sigma} = 0.1442]	7142 [R_{int} = 0.0497, R_{sigma} = 0.0629]
Data/restraints/parameters	9294/0/436	7142/0/444
Goodness-of-fit on F^2	1.002	1.055
Final R indexes [I>=2σ (I)]	R_1 = 0.1353, wR_2 = 0.3131	R_1 = 0.0561, wR_2 = 0.1279
Final R indexes [all data]	R_1 = 0.2200, wR_2 = 0.3497	R_1 = 0.0754, wR_2 = 0.1361
Largest diff. peak/hole / e $Å^{-3}$	0.77/-0.93	0.26/-0.61

Computational Details

HOMO -5.23 eV | LUMO -2.28 eV

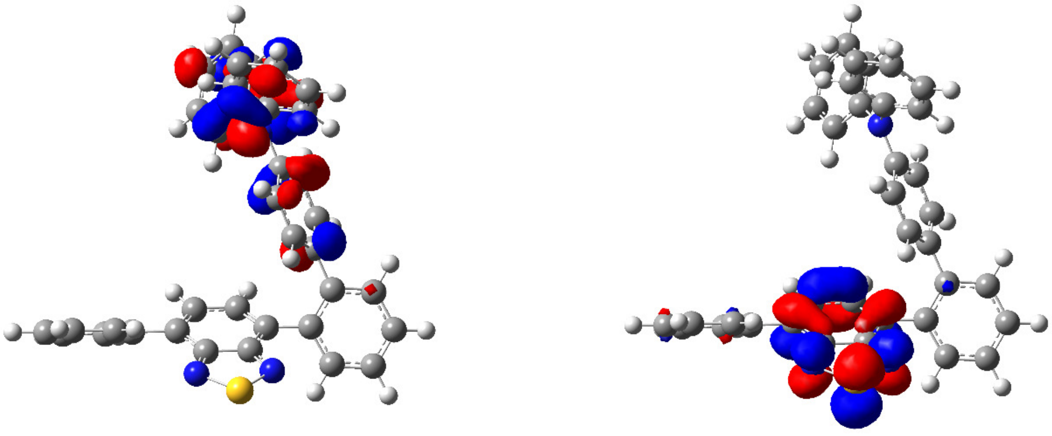

Figure S29: HOMO/LUMO plots of $\mathbf{3_H}$ (isovalue = 0.04).

HOMO -5.30 eV | LUMO -2.99 eV

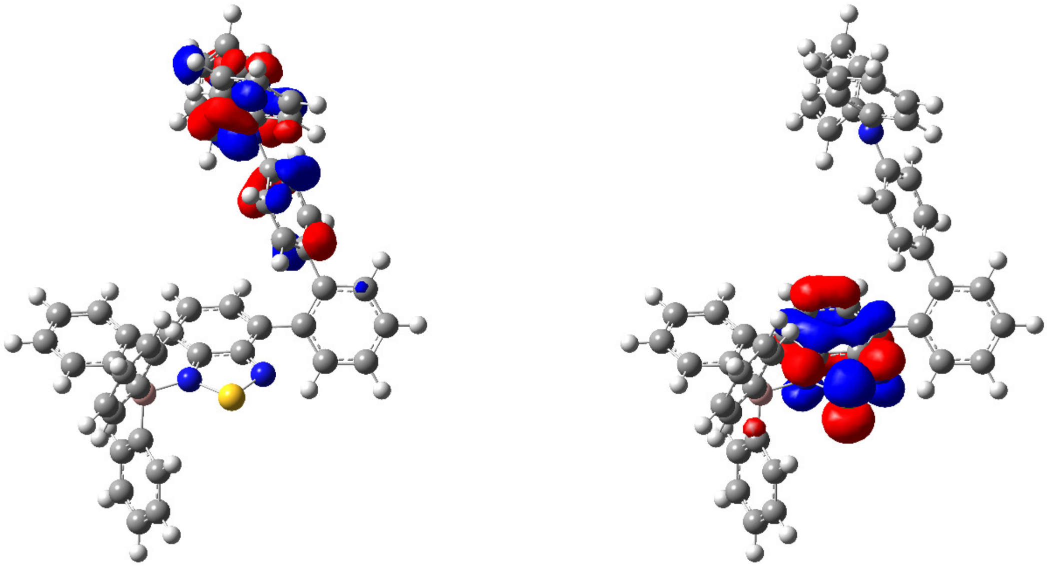

Figure S30: HOMO/LUMO plots of $\mathbf{3_H\text{-}BPh_2}$ (isovalue = 0.04).

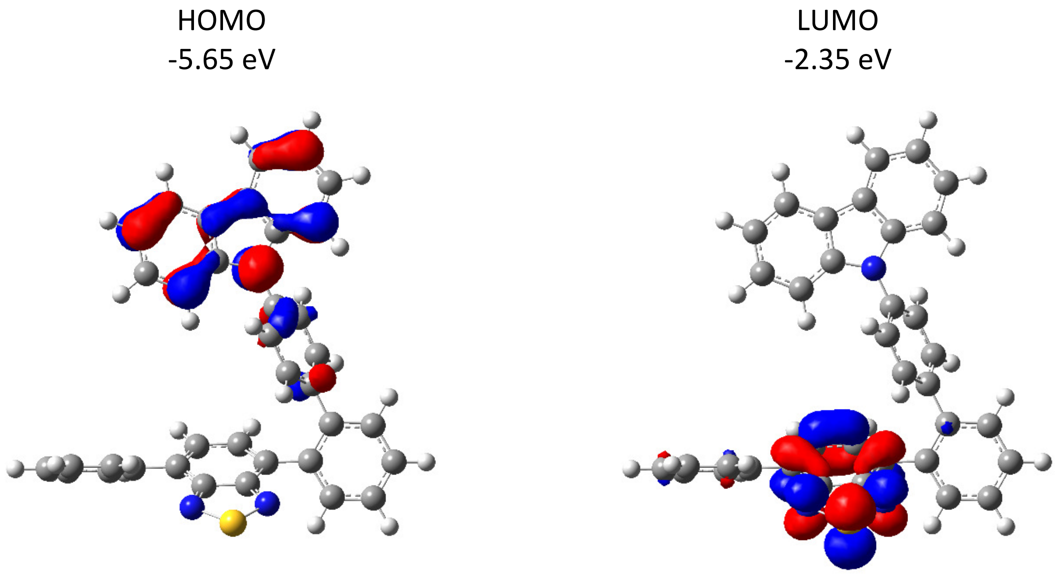

Figure S31: HOMO/LUMO plots of $\mathbf{4_H}$ (isovalue = 0.04).

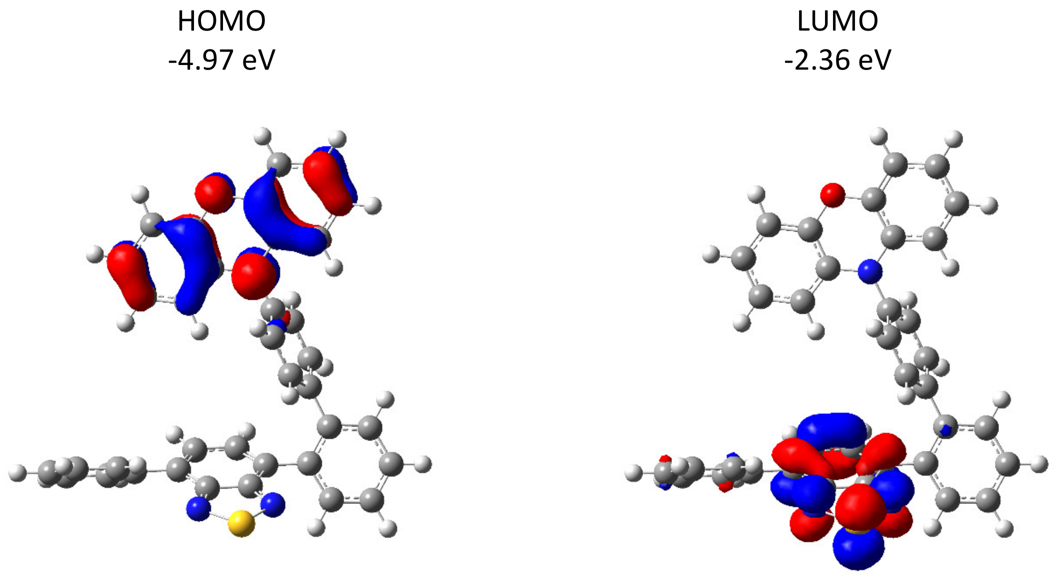

Figure S32: HOMO/LUMO plots of $\mathbf{5_H}$ (isovalue = 0.04).

HOMO
-5.16 eV

LUMO
-2.21 eV

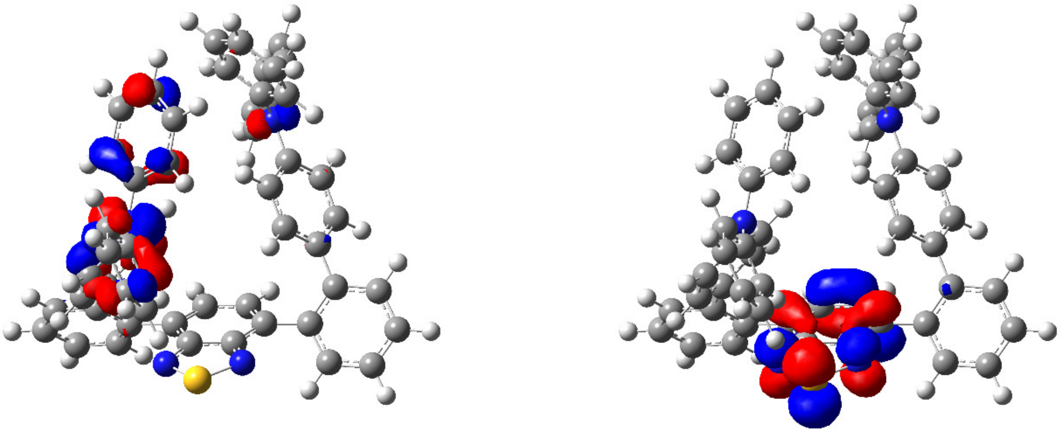

Figure S33: HOMO/LUMO plots of $\mathbf{9_H}$ (isovalue = 0.04).

HOMO
-5.13 eV

LUMO
-2.21 eV

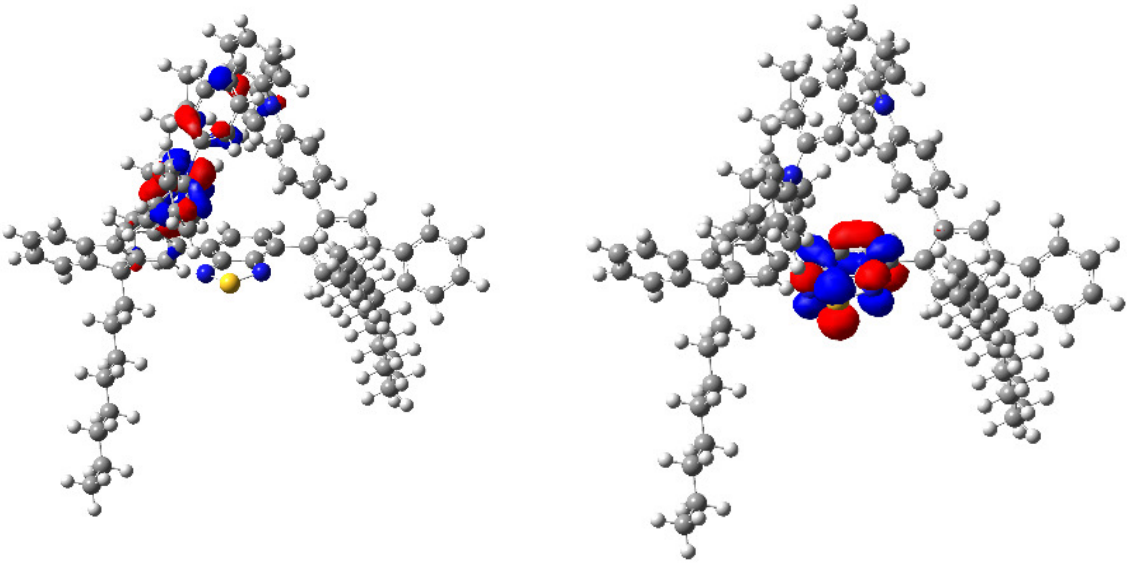

Figure S34: HOMO/LUMO plots of **10** (isovalue = 0.04).

HOMO
-5.15 eV

LUMO
-2.34 eV

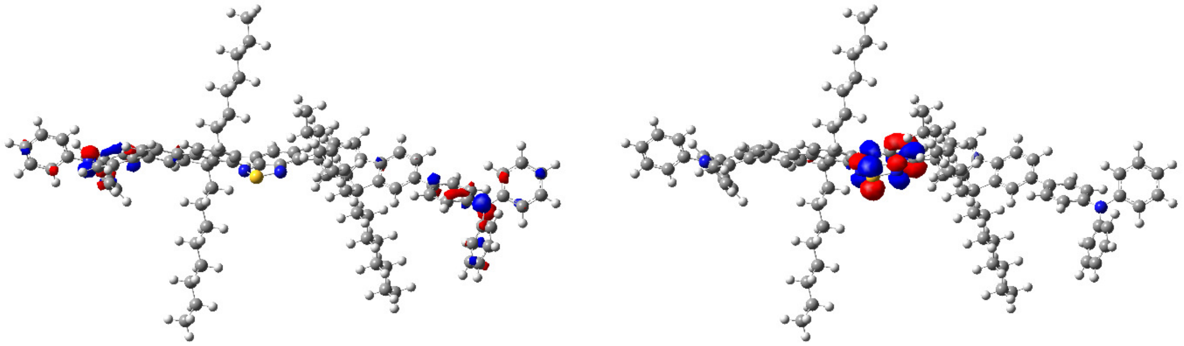

Figure S35: HOMO/LUMO plots of **11** (isovalue = 0.04).

Table S4: Calculated singlet-triplet splitting (ΔE_{ST}), spin-orbit coupling (SOC), reorganisation energies (λ) and (reverse) intersystem crossing ((R)ISC) at the PBE0/6-31G(d,p)/ PCM(toluene) level of theory.

**Compound**	**State**	**ΔE_{ST} (eV)**	**SOC (cm^{-1})**	**λ (eV)**	**(R)ISC (s^{-1})**
**3_H**	S_1	+0.83	0.16	0.17	1.00×10^{-4}
	T_1	-0.83	0.24	0.22	1.48×10^{-14}
**3_H-BPh_2**	S_1	+0.62	0.16	0.16	2.96×10^{1}
	T_1	-0.62	0.23	0.23	1.47×10^{-6}
**4_H**	S_1	+1.15	0.11	0.12	9.99×10^{-30}
	T_1	-1.15	0.27	0.18	3.37×10^{-34}
**5_H**	S_1	+0.47	0.18	0.30	6.05×10^{6}
	T_1	-0.47	0.14	0.45	7.06×10^{-2}
**9_H**	S_1	+0.66	0.17	0.26	3.32×10^{4}
	T_1	-0.66	0.39	0.30	1.02×10^{-5}
**10**	S_1	+0.68	0.19	0.22	2.59×10^{3}
	T_1	-0.68	0.40	0.27	7.76×10^{-7}
**11**	S_1	+0.89	0.14	0.08	1.06×10^{-30}
	T_1	-0.89	0.17	0.11	1.84×10^{-32}

Atomic coordinates of optimised ground-state 3_H

S	-5.019838	-0.748442	2.201689
N	-4.719230	0.678049	1.482914
N	-4.053617	-1.725269	1.334069
C	-3.827953	0.441241	0.516039
C	-3.447505	-0.954571	0.426022
C	-3.279303	1.415458	-0.380365
C	-2.503583	-1.406628	-0.549345
C	-2.374359	0.929428	-1.298900
C	-1.999446	-0.435731	-1.382976
H	-1.906007	1.626029	-1.988053
H	-1.277360	-0.722408	-2.141110
C	-2.135224	-2.835392	-0.679107
C	-3.162909	-3.779540	-0.803440
C	-2.889921	-5.120469	-1.038732
C	-1.568967	-5.543434	-1.152570
C	-0.538814	-4.622862	-1.008705
C	-0.792783	-3.267349	-0.761495
H	-4.192161	-3.443156	-0.732431
H	-3.706263	-5.829550	-1.138728
H	-1.339691	-6.589142	-1.335802
H	0.494145	-4.956552	-1.054869
C	0.364978	-2.364300	-0.548810
C	1.436243	-2.348185	-1.449480
C	2.551028	-1.548386	-1.234110
C	2.625416	-0.725362	-0.104191
C	1.558204	-0.736068	0.804283
C	0.454618	-1.546029	0.584839
H	1.395321	-2.974096	-2.337093
H	3.370801	-1.555510	-1.945496
H	1.603030	-0.107132	1.687723
H	-0.356383	-1.545130	1.307308
C	4.274989	0.243624	1.420334
C	4.720342	1.493195	1.869456
C	5.245628	1.630663	3.148980
C	5.318910	0.535758	4.007146
C	4.867379	-0.705852	3.565540
C	4.357832	-0.857830	2.281220
H	4.652413	2.352042	1.209115
H	5.586555	2.607176	3.481211
H	5.722924	0.648855	5.008463
H	4.924341	-1.570798	4.220363
H	4.020263	-1.830215	1.936552
C	4.360072	0.765830	-0.969015
C	3.576770	1.360781	-1.965944
C	4.180260	2.012101	-3.035324
C	5.568001	2.098815	-3.121328
C	6.348654	1.515771	-2.125785
C	5.755149	0.845991	-1.062103
H	2.494889	1.308720	-1.894582

H	3.557663	2.468906	-3.799485
H	6.035475	2.615007	-3.954190
H	7.432260	1.568552	-2.182240
H	6.367623	0.381666	-0.295604
N	3.750951	0.096007	0.116109
C	-3.654714	2.842132	-0.342405
C	-3.771097	3.565057	-1.538537
C	-4.085083	4.918913	-1.526998
C	-4.291623	5.578414	-0.317960
C	-4.185810	4.870378	0.876600
C	-3.874164	3.515468	0.867637
H	-3.639439	3.051781	-2.486917
H	-4.178486	5.457355	-2.465726
H	-4.539414	6.635808	-0.307139
H	-4.344693	5.376337	1.824576
H	-3.794417	2.972832	1.802663

Atomic coordinates of optimised ground-state 3_H-BPh$_2$

S	-3.407212	1.273719	-1.669195
N	-3.207398	0.257421	-0.372722
N	-2.142496	2.229619	-1.377245
C	-1.617438	-0.017996	1.458259
C	-2.127731	0.643088	0.308222
C	-0.347334	2.382252	0.311650
C	-2.282329	-1.237562	1.934435
C	1.669348	3.718765	-0.444196
C	2.361295	1.358339	-0.977979
H	1.452275	1.237420	-1.560058
C	2.603466	2.573018	-0.323315
C	-1.516274	1.806945	-0.275603
N	5.371296	-0.628548	-0.107057
C	0.115315	1.735660	1.436913
C	5.601567	-1.437247	-1.245040
C	3.260542	0.306227	-0.903008
H	3.048831	-0.625554	-1.417545
C	-0.004427	5.943920	-0.853392
H	-0.658347	6.795899	-1.012767
C	2.188373	4.958423	-0.839613
H	3.256000	5.037679	-1.024277
C	-3.463635	-1.680579	1.296274
C	4.707330	1.649806	0.476134
H	5.625545	1.770752	1.041911
C	1.369678	6.062929	-1.038456
C	4.451824	0.436451	-0.174742
C	-0.538622	4.726919	-0.451982
H	-1.607151	4.638312	-0.283428
C	3.794504	2.693640	0.402510
H	4.010919	3.624336	0.920431
C	6.082871	-0.883885	1.087196
C	0.277452	3.610109	-0.226674
C	-0.490644	0.578301	1.989517
C	-4.045452	-2.862207	1.777193
H	-4.948284	-3.229272	1.294992
C	-3.506937	-3.579068	2.837191
C	-2.338762	-3.129400	3.453895
H	-1.901485	-3.684627	4.278563
C	5.720248	-0.859251	-2.514705
H	5.638396	0.218137	-2.619765
C	-1.732419	-1.970766	3.000364
H	-0.815962	-1.637676	3.477804
C	7.439492	-1.228178	1.043250
H	7.943031	-1.287858	0.083568
C	5.441364	-0.803275	2.329571
H	4.387647	-0.545296	2.368794
C	5.717481	-2.826203	-1.114297
H	5.620949	-3.278477	-0.132190
C	6.148387	-1.052517	3.500124

H	5.635483	-0.986495	4.455512
C	5.939570	-1.659676	-3.629900
H	6.028945	-1.196028	-4.608217
C	6.063240	-3.040797	-3.497130
H	6.241523	-3.662142	-4.369353
C	7.495870	-1.403107	3.453719
H	8.042648	-1.604538	4.369710
C	5.955493	-3.616310	-2.232979
H	6.042569	-4.692676	-2.115144
C	8.132990	-1.493384	2.218327
H	9.184898	-1.759569	2.166077
H	1.802495	7.008193	-1.352494
H	-3.989461	-4.490229	3.180214
H	-0.029438	0.157979	2.876780
H	0.996389	2.130341	1.932812
5	-4.221726	-0.914306	0.082412
C	-4.440175	-1.895161	-1.194463
C	-5.663274	-2.542189	-1.427023
C	-3.385989	-2.209556	-2.067380
C	-5.830180	-3.446813	-2.474140
H	-6.508198	-2.325536	-0.777684
C	-3.542096	-3.104478	-3.122689
H	-2.408865	-1.753452	-1.914467
C	-4.769838	-3.728334	-3.330962
H	-6.793375	-3.928095	-2.624400
H	-2.703238	-3.320284	-3.779699
H	-4.897494	-4.428362	-4.152206
C	-5.570477	-0.134723	0.570030
C	-6.474802	0.400029	-0.362112
C	-5.852791	0.094050	1.923696
C	-7.596996	1.126010	0.029511
H	-6.310716	0.228127	-1.425045
C	-6.974688	0.815482	2.329711
H	-5.181269	-0.305131	2.679940
C	-7.851620	1.336541	1.382840
H	-8.277267	1.520702	-0.721090
H	-7.163038	0.971557	3.389056
H	-8.727361	1.898991	1.695379

Atomic coordinates of optimised ground-state 4_H

S	-5.041356	-0.697739	2.257270
N	3.648707	0.109916	0.074202
N	-4.071640	-1.744316	1.479393
N	-4.744687	0.660268	1.415561
C	-1.529908	-5.752753	-0.607321
C	-2.857152	-5.338342	-0.551780
H	-3.662849	-6.065383	-0.588531
C	-3.151342	-3.984521	-0.456167
H	-4.185646	-3.657497	-0.429533
C	-2.137948	-3.018249	-0.415952
C	-0.790196	-3.439261	-0.442357
C	-0.513306	-4.808046	-0.548002
H	0.524407	-5.129924	-0.550480
C	0.352054	-2.500451	-0.315791
C	0.411635	-1.566047	0.726053
H	-0.396733	-1.521476	1.449501
C	1.501456	-0.719341	0.866504
H	1.551163	-0.020178	1.695316
C	2.553699	-0.772945	-0.051585
C	2.510436	-1.700153	-1.093645
H	3.317620	-1.729425	-1.818870
C	1.425583	-2.561303	-1.211643
H	1.393959	-3.271584	-2.033195
C	3.563353	1.495988	0.159490
C	2.437190	2.317460	0.106361
H	1.440713	1.902775	-0.005127
C	2.636721	3.689359	0.195947
H	1.775917	4.350711	0.159421
C	3.921352	4.236266	0.329184
H	4.040184	5.313199	0.399103
C	5.039012	3.414024	0.366275
H	6.034694	3.838748	0.458992
C	4.866787	2.030807	0.277573
C	5.778219	0.911392	0.264412
C	7.166762	0.797793	0.361795
H	7.784983	1.685815	0.459992
C	7.743432	-0.464625	0.337178
H	8.821719	-0.567838	0.410750
C	6.944762	-1.611958	0.224576
H	7.415154	-2.590948	0.217482
C	5.561614	-1.527254	0.125566
H	4.949649	-2.419826	0.049407
C	4.989353	-0.255300	0.136645
C	-2.522207	-1.587636	-0.424874
C	-3.467983	-1.054837	0.506713
C	-3.852405	0.342207	0.473323
C	-3.305720	1.236067	-0.504584
C	-2.402356	0.672836	-1.379925
H	-1.936591	1.306605	-2.128820

C	-2.024421	-0.693776	-1.344125
H	-1.302876	-1.043334	-2.076116
C	-3.679748	2.661230	-0.587720
C	-3.906016	3.432954	0.560902
H	-3.835247	2.969374	1.538215
C	-4.213677	4.784727	0.455186
H	-4.378176	5.367902	1.356682
C	-4.308482	5.391374	-0.794739
C	-4.095840	4.633390	-1.943525
H	-4.181155	5.092453	-2.924210
C	-3.786013	3.282535	-1.840553
H	-3.649837	2.692488	-2.742412
H	-4.552818	6.446687	-0.873361
H	-1.285382	-6.808272	-0.680976

Atomic coordinates of optimised ground-state 5_H

S	-4.984268	-0.962215	2.371140
N	3.554719	0.191960	-0.068843
N	-4.039441	-1.928852	1.469145
N	-4.740120	0.463556	1.630667
C	-1.543292	-5.713454	-1.063776
C	-2.872099	-5.328308	-0.913462
H	-3.668429	-6.063724	-0.977708
C	-3.180948	-3.993123	-0.688526
H	-4.217461	-3.687790	-0.589214
C	-2.179624	-3.017026	-0.608935
C	-0.829457	-3.411853	-0.728924
C	-0.537330	-4.760062	-0.967234
H	0.503602	-5.061527	-1.045041
C	0.301360	-2.464302	-0.561599
C	0.401465	-1.661522	0.582178
H	-0.365810	-1.726694	1.347772
C	1.475436	-0.798836	0.754296
H	1.554607	-0.184455	1.646135
C	2.465469	-0.710562	-0.226096
C	2.382849	-1.509540	-1.365058
H	3.156516	-1.434495	-2.123218
C	1.314308	-2.385855	-1.524074
H	1.248610	-2.999155	-2.418481
C	3.378852	1.560561	-0.308830
C	2.159775	2.108536	-0.709838
H	1.302930	1.455996	-0.836807
C	2.034310	3.477890	-0.944643
H	1.072345	3.875032	-1.253669
C	3.124282	4.321157	-0.782979
H	3.034007	5.387523	-0.962245
C	4.349268	3.784822	-0.385408
H	5.227683	4.408111	-0.251431
C	4.474513	2.425798	-0.156902
C	5.846060	0.646180	0.554444
C	7.288306	-1.090145	1.398168
H	8.258757	-1.402000	1.770229
C	6.246247	-1.998764	1.280300
H	6.387578	-3.037562	1.562125
C	5.002255	-1.588172	0.800950
H	4.190039	-2.301585	0.714944
C	4.783718	-0.262687	0.424243
C	-2.578959	-1.596406	-0.481728
C	-3.487586	-1.154325	0.530395
C	-3.887748	0.235203	0.627376
C	-3.395273	1.212507	-0.298271
C	-2.526769	0.734879	-1.255588
C	-2.132628	-0.624544	-1.346755
C	-3.788467	2.633867	-0.248068
C	-3.959371	3.308078	0.969276

H	-3.828245	2.769889	1.901036
C	-4.287533	4.658886	0.989319
H	-4.407790	5.166019	1.942280
C	-4.458719	5.361295	-0.200980
C	-4.300905	4.700626	-1.416728
H	-4.445520	5.234835	-2.351330
C	-3.970049	3.350929	-1.439624
H	-3.876498	2.836465	-2.391896
H	-4.719271	6.415466	-0.181442
H	-1.288633	-6.754208	-1.240585
H	-2.103795	1.433597	-1.971503
H	-1.442606	-0.904343	-2.136807
O	5.717724	1.968254	0.205682
C	7.078657	0.240531	1.034949
H	7.865543	0.983805	1.115173

Atomic coordinates of optimised ground-state 9_H

S	3.768973	-2.900395	-1.215899
N	3.336995	-2.876718	0.349939
N	2.330149	-3.186275	-1.918579
C	2.019207	-3.085879	0.382244
C	1.436936	-3.279222	-0.928721
C	1.213323	-3.139732	1.562384
C	0.038497	-3.545794	-1.084575
C	-0.117456	-3.426916	1.366511
C	-0.688386	-3.622401	0.080680
H	-0.769559	-3.489643	2.233430
H	-1.751197	-3.834618	0.019169
C	-0.568793	-3.779803	-2.415203
C	0.029378	-4.720035	-3.264946
C	-0.542416	-5.064868	-4.482264
C	-1.735317	-4.465966	-4.875628
C	-2.329621	-3.517492	-4.053355
C	-1.764947	-3.149651	-2.824684
H	0.951063	-5.197100	-2.948615
H	-0.060149	-5.802273	-5.117000
H	-2.194094	-4.723730	-5.825743
H	-3.239778	-3.018646	-4.374527
C	-2.438570	-2.083747	-2.041947
C	-3.813835	-2.150797	-1.789394
C	-4.477506	-1.135381	-1.113620
C	-3.777360	-0.012367	-0.657916
C	-2.399034	0.063004	-0.903642
C	-1.747704	-0.952572	-1.587533
H	-4.375802	-3.016408	-2.130254
H	-5.545416	-1.208815	-0.934231
H	-1.843646	0.931330	-0.562590
H	-0.681042	-0.866864	-1.773983
C	-4.117675	2.373270	-0.229426
C	-4.059549	3.300310	0.818600
C	-3.754819	4.631233	0.559628
C	-3.484948	5.057038	-0.739022
C	-3.531273	4.134239	-1.781128
C	-3.854149	2.804811	-1.535254
H	-4.257036	2.969596	1.833431
H	-3.714633	5.337219	1.384359
H	-3.241525	6.096411	-0.936900
H	-3.328748	4.451903	-2.800008
H	-3.903929	2.092683	-2.352942

C	-5.475048	0.713327	0.946204
C	-5.334114	-0.356022	1.839646
C	-6.352891	-0.659518	2.735205
C	-7.517608	0.104127	2.769976
C	-7.655972	1.173550	1.888167
C	-6.650673	1.473727	0.976408
H	-4.421931	-0.944458	1.825910
H	-6.226551	-1.491901	3.421802
H	-8.307764	-0.131415	3.476237
H	-8.561204	1.774110	1.897910
H	-6.768302	2.300395	0.282913
N	-4.444287	1.022449	0.029854
C	1.783838	-2.962871	2.917799
C	1.612944	-4.002386	3.839841
C	2.149174	-3.932235	5.120031
C	2.873845	-2.806124	5.496467
C	3.036590	-1.759706	4.596516
C	2.496163	-1.806722	3.305314
H	1.073947	-4.891956	3.525671
H	2.009279	-4.756268	5.813302
H	3.301484	-2.734849	6.492312
H	3.569413	-0.863946	4.903399
C	2.662006	-0.626302	2.425098
C	3.918454	-0.027608	2.272391
C	1.578088	-0.044964	1.756095
C	4.092062	1.099014	1.482091
H	4.778719	-0.462411	2.773507
C	1.743527	1.073794	0.951351
H	0.587171	-0.476119	1.861957
C	3.005552	1.662596	0.801594
H	5.076485	1.544379	1.378532
H	0.889070	1.504543	0.438532
C	4.326241	2.906679	-0.837792
C	4.980680	4.136336	-0.984138
C	4.822704	1.787366	-1.517955
C	6.100556	4.240920	-1.800804
H	4.604295	5.006133	-0.454961
C	5.953890	1.898702	-2.317886
H	4.316307	0.832633	-1.414347
C	6.598168	3.124548	-2.469195
H	6.595711	5.202530	-1.902763
H	6.324593	1.020335	-2.838726
H	7.477300	3.208934	-3.100597
C	2.204463	3.819138	-0.029386

C	1.597723	4.234126	1.162683
C	1.828807	4.429523	-1.232482
C	0.628623	5.230157	1.145205
H	1.889692	3.768689	2.098862
C	0.872338	5.437989	-1.236594
H	2.290433	4.107060	-2.160457
C	0.262530	5.842018	-0.051290
H	0.165894	5.537848	2.078643
H	0.591019	5.899308	-2.179041
H	-0.491568	6.622697	-0.060075
N	3.181221	2.798003	-0.017191

Atomic coordinates of optimised ground-state 10

S	1.414023	1.127668	-3.187038
N	-0.153208	0.992596	-2.781404
N	2.107936	1.086758	-1.716048
C	-0.193344	0.897481	-1.451151
C	1.113789	0.966846	-0.831027
C	-1.376324	0.741559	-0.662487
C	1.261147	0.905195	0.592837
C	-1.190031	0.751232	0.701041
C	0.089032	0.832463	1.309975
H	-2.059190	0.646858	1.344761
H	0.141553	0.803671	2.393599
C	2.588297	0.937369	1.245940
C	2.940192	0.021961	2.268363
C	3.492663	1.947726	0.878553
C	4.174313	0.160446	2.915666
C	4.709092	2.069428	1.524249
H	3.212777	2.640487	0.090573
C	5.049984	1.175356	2.550520
H	4.445596	-0.557111	3.685607
C	5.812228	3.088424	1.297293
C	6.379169	1.528724	3.050543
C	6.837225	2.643862	2.327819
C	5.324120	4.531462	1.563079
C	6.374570	3.019191	-0.140493
C	7.163205	0.954975	4.049187
C	8.082943	3.189939	2.603333
H	6.174901	5.209522	1.407883
H	4.588149	4.787104	0.788100
C	4.717294	4.786564	2.938172
H	5.589309	3.361811	-0.828422
H	7.188303	3.753835	-0.218641
C	6.878183	1.653700	-0.593833
C	8.412898	1.507647	4.319780
H	6.810964	0.091930	4.607798
C	8.869581	2.615537	3.603819
H	8.447633	4.052727	2.051029
H	3.859424	4.118637	3.089013
H	5.447326	4.529774	3.716660
C	4.268052	6.232942	3.122140
H	7.648974	1.295306	0.100806
H	6.057164	0.927108	-0.546740
C	7.445439	1.681231	-2.009790

H	9.038251	1.072964	5.094300
H	9.846772	3.034056	3.827443
H	5.129014	6.903919	2.990068
H	3.554697	6.497717	2.328318
C	3.626676	6.496336	4.480671
H	6.675854	2.049415	-2.703427
H	8.269983	2.407180	-2.059970
C	7.942715	0.321039	-2.487800
H	2.765744	5.825025	4.609653
H	4.337572	6.230693	5.275953
C	3.172539	7.939790	4.670345
H	8.710776	-0.049162	-1.793861
H	7.117638	-0.403521	-2.438945
C	8.510395	0.344497	-3.903075
H	4.035185	8.611280	4.553375
H	2.470854	8.207988	3.867431
C	2.512646	8.196843	6.020992
H	7.742362	0.717291	-4.595976
H	9.336656	1.068400	-3.951446
C	9.003955	-1.015894	-4.383926
H	1.650381	7.524618	6.137490
H	3.213031	7.929862	6.825442
C	2.054060	9.639060	6.211479
H	9.772688	-1.389655	-3.692285
H	8.178156	-1.740192	-4.335693
C	9.570252	-0.993602	-5.800033
H	2.916609	10.310002	6.100800
H	1.358801	9.905849	5.404027
C	1.387424	9.881541	7.559762
H	8.801753	-0.619371	-6.489900
H	10.395857	-0.270598	-5.847385
C	10.058484	-2.357666	-6.271009
H	0.503099	9.246281	7.682183
H	2.071432	9.654633	8.385137
H	1.067016	10.922248	7.670322
H	10.850457	-2.740577	-5.617716
H	9.244872	-3.091571	-6.265510
H	10.459461	-2.313653	-7.288522
C	2.067522	-1.109945	2.673991
C	1.730947	-1.296175	4.019536
C	1.585553	-2.043333	1.746713
C	0.932090	-2.357254	4.425973
H	2.096404	-0.591068	4.761553
C	0.779891	-3.101852	2.140664

H	1.839383	-1.931698	0.696332
C	0.434849	-3.270022	3.489032
H	0.682059	-2.479248	5.475176
H	0.414326	-3.811120	1.404078
N	-0.400523	-4.332121	3.897122
C	-0.243110	-5.621245	3.342969
C	-1.387607	-4.104188	4.883922
C	-1.363029	-6.420486	3.078698
C	1.032444	-6.123409	3.054461
C	-2.172806	-2.945228	4.846431
C	-1.592904	-5.032335	5.912152
C	-1.204736	-7.697172	2.552668
H	-2.355218	-6.035415	3.291415
C	1.178401	-7.393490	2.509424
H	1.905743	-5.512661	3.260244
C	-3.134172	-2.718545	5.824840
H	-2.021339	-2.226802	4.046757
C	-2.568968	-4.805779	6.875559
H	-0.983667	-5.930028	5.948890
C	0.064508	-8.192592	2.261412
H	-2.084826	-8.303941	2.358614
H	2.175517	-7.765236	2.291380
C	-3.341929	-3.647049	6.842956
H	-3.734259	-1.813570	5.782941
H	-2.714481	-5.535433	7.667114
H	0.183922	-9.189710	1.848335
H	-4.095856	-3.467551	7.603430
C	-2.727582	0.594991	-1.247929
C	-3.054192	-0.426432	-2.173653
C	-3.719523	1.490698	-0.818306
C	-4.365272	-0.501474	-2.662251
C	-5.012755	1.393581	-1.299450
H	-3.445621	2.271174	-0.112217
C	-5.334529	0.395382	-2.230374
H	-4.623242	-1.303604	-3.348603
C	-6.231945	2.223973	-0.940221
C	-6.760331	0.500664	-2.545608
C	-7.298472	1.562011	-1.797438
C	-6.048033	3.715217	-1.301236
C	-6.555724	2.119051	0.568336
C	-7.565166	-0.247569	-3.401808
C	-8.645203	1.879130	-1.904695
H	-6.972035	4.244825	-1.029850
H	-5.262208	4.126707	-0.652459

C	-5.704858	4.009323	-2.757204
H	-5.742902	2.612871	1.119319
H	-7.459245	2.711697	0.767285
C	-6.741020	0.705102	1.108625
C	-8.916416	0.075332	-3.503710
H	-7.151077	-1.068390	-3.981160
C	-9.452760	1.129409	-2.762462
H	-9.072038	2.698616	-1.331250
H	-4.776000	3.490745	-3.027367
H	-6.486791	3.598922	-3.409135
C	-5.548107	5.502006	-3.030864
H	-7.598429	0.229911	0.615132
H	-5.864056	0.098213	0.848565
C	-6.936251	0.668804	2.621444
H	-9.558903	-0.497574	-4.166187
H	-10.508471	1.368352	-2.854241
H	-6.481166	6.021847	-2.769372
H	-4.775887	5.915982	-2.366426
C	-5.184766	5.816626	-4.478315
H	-6.092423	1.180081	3.107521
H	-7.833653	1.242022	2.894946
C	-7.047615	-0.747678	3.176609
H	-4.250822	5.298373	-4.737838
H	-5.954656	5.402157	-5.144547
C	-5.027402	7.308263	-4.754219
H	-7.906283	-1.254531	2.714270
H	-6.160835	-1.322849	2.874004
C	-7.184583	-0.801603	4.694402
H	-5.964012	7.825634	-4.501110
H	-4.262919	7.723015	-4.081504
C	-4.651163	7.624669	-6.197788
H	-6.331656	-0.279821	5.153137
H	-8.079393	-0.241492	5.001701
C	-7.261101	-2.221464	5.246206
H	-3.714267	7.107920	-6.450890
H	-5.414543	7.211050	-6.872409
C	-4.491768	9.116463	-6.472971
H	-8.115754	-2.743125	4.792194
H	-6.366421	-2.780444	4.936363
C	-7.386008	-2.280398	6.765069
H	-5.429304	9.631786	-6.224068
H	-3.731915	9.528758	-5.795305
C	-4.108297	9.421678	-7.915466
H	-6.528114	-1.763673	7.217608

H	-8.275307	-1.716705	7.078070
C	-7.466685	-3.703679	7.302788
H	-3.157618	8.945399	-8.179999
H	-4.866980	9.049908	-8.613173
H	-3.999554	10.497502	-8.085103
H	-8.334444	-4.231771	6.892163
H	-6.574101	-4.278436	7.031139
H	-7.555026	-3.718793	8.393799
C	-2.086633	-1.461018	-2.610352
C	-1.992374	-1.818343	-3.960888
C	-1.268854	-2.146945	-1.703650
C	-1.120518	-2.807116	-4.392979
H	-2.607996	-1.301203	-4.691679
C	-0.373732	-3.118517	-2.127579
H	-1.324102	-1.914030	-0.644682
C	-0.284633	-3.467083	-3.482314
H	-1.073173	-3.063371	-5.446413
H	0.257817	-3.622129	-1.402753
N	0.620570	-4.452579	-3.921964
C	0.947736	-5.551218	-3.094404
C	1.253559	-4.323056	-5.181261
C	2.270847	-6.001269	-3.007839
C	-0.042375	-6.203102	-2.348310
C	1.770430	-3.090612	-5.598155
C	1.375276	-5.433998	-6.024179
C	2.591455	-7.082010	-2.194366
H	3.043659	-5.497168	-3.579412
C	0.292533	-7.265920	-1.518034
H	-1.071361	-5.864384	-2.416320
C	2.386184	-2.974596	-6.839241
H	1.683676	-2.228051	-4.944378
C	2.008865	-5.312513	-7.255485
H	0.971977	-6.389903	-5.704485
C	1.608529	-7.716045	-1.438597
H	3.623411	-7.417056	-2.138884
H	-0.483208	-7.747839	-0.930239
C	2.513306	-4.083031	-7.673475
H	2.782218	-2.011547	-7.148800
H	2.095488	-6.184046	-7.898165
H	1.864146	-8.549136	-0.791115
H	3.000890	-3.989697	-8.639045

Atomic coordinates of optimised ground-state 11

S	0.746683	-1.540673	-3.601436
N	-0.702937	-1.525898	-2.867661
N	1.728238	-1.489017	-2.307739
C	-0.470373	-1.483554	-1.553137
C	0.943233	-1.453602	-1.227601
C	-1.471950	-1.455353	-0.527036
C	1.393207	-1.403825	0.133421
C	-0.987942	-1.406423	0.762970
C	0.392117	-1.377042	1.081047
H	-1.696690	-1.405698	1.585861
H	0.667977	-1.315899	2.129634
C	2.819078	-1.394505	0.505719
C	3.239935	-2.066518	1.666974
C	3.774301	-0.707324	-0.264364
C	4.569363	-2.059278	2.070297
H	2.512621	-2.630161	2.243959
C	5.099390	-0.698496	0.133589
H	3.463307	-0.189939	-1.165702
C	5.504076	-1.370658	1.299598
H	4.868809	-2.597454	2.965473
C	6.281775	-0.030180	-0.548388
C	6.945746	-1.195673	1.456226
C	7.417114	-0.419341	0.383752
C	6.107127	1.502936	-0.634599
C	6.499365	-0.580120	-1.976805
C	7.829720	-1.653010	2.431445
C	8.760545	-0.100405	0.285113
H	7.004631	1.918549	-1.113525
H	5.274933	1.709079	-1.321999
C	5.859451	2.222472	0.686477
H	5.633701	-0.286433	-2.586338
H	7.366660	-0.062287	-2.410039
C	6.703322	-2.087030	-2.082842
C	9.178196	-1.334253	2.322531
H	7.480501	-2.261646	3.261207
C	9.667017	-0.559063	1.257034
H	9.119233	0.525120	-0.528933
H	4.953675	1.820683	1.158635
H	6.685280	2.015990	1.379487
C	5.710811	3.730403	0.509218
H	7.576600	-2.382868	-1.486962
H	5.840762	-2.604911	-1.644405

C	6.891957	-2.556334	-3.522336
H	9.876679	-1.716280	3.061405
H	6.624574	4.136571	0.052077
H	4.899808	3.935457	-0.204520
C	5.428957	4.469311	1.813416
H	6.012168	-2.271962	-4.117166
H	7.745215	-2.028016	-3.972066
C	7.113782	-4.060725	-3.637787
H	4.512386	4.065980	2.266902
H	6.236231	4.263135	2.530481
C	5.283107	5.976978	1.636562
H	7.996291	-4.342851	-3.046048
H	6.264022	-4.587862	-3.181353
C	7.293924	-4.544055	-5.073065
H	6.204010	6.381509	1.192611
H	4.483502	6.181335	0.910020
C	4.982847	6.717048	2.935761
H	6.408222	-4.268369	-5.663305
H	8.139881	-4.012774	-5.532554
C	7.524132	-6.047523	-5.182016
H	4.060304	6.314571	3.378358
H	5.780335	6.512563	3.664577
C	4.838485	8.225073	2.758412
H	8.411728	-6.322845	-4.594302
H	6.680319	-6.579211	-4.719145
C	7.699695	-6.536892	-6.616062
H	5.762186	8.627009	2.320621
H	4.044790	8.427667	2.026581
C	4.530627	8.954080	4.060222
H	6.811822	-6.263265	-7.202034
H	8.542065	-6.004778	-7.078495
C	7.930730	-8.039647	-6.711076
H	3.594613	8.594326	4.501870
H	5.324286	8.795783	4.798933
H	4.431666	10.033008	3.904973
H	8.831531	-8.336185	-6.162212
H	7.088549	-8.596227	-6.284934
H	8.052285	-8.364401	-7.749235
C	11.103976	-0.236539	1.155079
C	11.886246	-0.005110	2.295546
C	11.740952	-0.148859	-0.091034
C	13.234181	0.306890	2.200252
H	11.421217	-0.028637	3.277152
C	13.091145	0.147562	-0.197521

H	11.176745	-0.358417	-0.995540
C	13.860104	0.385384	0.949211
H	13.810002	0.504822	3.098783
H	13.563325	0.186603	-1.174050
N	15.230014	0.698556	0.844291
C	15.683276	1.539795	-0.197909
C	16.152701	0.178286	1.779584
C	16.865364	1.242926	-0.887353
C	14.955930	2.682157	-0.554186
C	16.041308	-1.143619	2.228894
C	17.194268	0.977236	2.267643
C	17.310526	2.078429	-1.905263
H	17.430569	0.355811	-0.619023
C	15.399778	3.501221	-1.585804
H	14.042956	2.921494	-0.017911
C	16.948134	-1.647337	3.154121
H	15.241084	-1.770358	1.847971
C	18.106772	0.458060	3.178589
H	17.282510	2.004157	1.927381
C	16.580280	3.208886	-2.265151
H	18.229574	1.833491	-2.430135
H	14.823647	4.383708	-1.849377
C	17.988600	-0.853803	3.632000
H	16.847265	-2.675002	3.491592
H	18.908695	1.091588	3.547012
H	16.927604	3.854910	-3.065569
H	18.699303	-1.253203	4.348963
C	-2.917604	-1.464006	-0.812929
C	-3.452405	-2.223141	-1.867913
C	-3.792996	-0.724305	0.003632
C	-4.820876	-2.263228	-2.105724
H	-2.781975	-2.790534	-2.502697
C	-5.155857	-0.760014	-0.231097
H	-3.387412	-0.103695	0.799193
C	-5.675940	-1.531465	-1.284650
H	-5.210099	-2.863542	-2.923467
C	-6.265140	-0.030690	0.509616
C	-7.127867	-1.374977	-1.297612
C	-7.490911	-0.509964	-0.251284
C	-6.100286	1.503962	0.416547
C	-6.321341	-0.426176	2.002101
C	-8.104559	-1.915329	-2.131501
C	-8.820067	-0.187531	-0.035648
H	-6.936293	1.966300	0.959668

H	-5.190918	1.779950	0.968519
C	-6.031732	2.081800	-0.992389
H	-5.405097	-0.058119	2.484919
H	-7.149150	0.127288	2.466988
C	-6.483051	-1.913974	2.293054
C	-9.436666	-1.588445	-1.908477
H	-7.838018	-2.591678	-2.939125
C	-9.818160	-0.726071	-0.866407
H	-9.098524	0.503998	0.756111
H	-5.196201	1.624150	-1.537503
H	-6.942389	1.813468	-1.543300
C	-5.867153	3.598647	-0.998408
H	-7.402576	-2.282410	1.820407
H	-5.656980	-2.471425	1.832634
C	-6.524384	-2.216100	3.787930
H	-10.205877	-2.029938	-2.535482
H	-6.701841	4.059593	-0.450760
H	-4.956250	3.870594	-0.445739
C	-5.798725	4.189578	-2.402684
H	-5.606188	-1.839487	4.261710
H	-7.351897	-1.659758	4.251385
C	-6.678431	-3.700725	4.101322
H	-4.964542	3.727859	-2.949715
H	-6.709062	3.916421	-2.955046
C	-5.634577	5.705727	-2.419204
H	-7.600078	-4.076769	3.635060
H	-5.854806	-4.257448	3.632120
C	-6.706754	-4.006025	5.595263
H	-6.469388	6.167730	-1.872792
H	-4.724299	5.979045	-1.866314
C	-5.565354	6.293677	-3.824530
H	-5.785107	-3.627344	6.060266
H	-7.531153	-3.450217	6.064645
C	-6.856156	-5.490448	5.910972
H	-4.730961	5.831571	-4.371462
H	-6.475473	6.021012	-4.378036
C	-5.400129	7.809921	-3.844417
H	-7.780277	-5.869503	5.451425
H	-6.034564	-6.047346	5.437907
C	-6.875276	-5.796333	7.405204
H	-6.234393	8.271080	-3.298672
H	-4.490926	8.081054	-3.290820
C	-5.330590	8.386050	-5.252953
H	-5.951965	-5.415746	7.862661

H	-7.696921	-5.240652	7.877058
C	-7.021422	-7.281608	7.710645
H	-4.485357	7.966501	-5.809863
H	-6.241335	8.158763	-5.818194
H	-5.211559	9.473994	-5.238340
H	-7.953416	-7.680871	7.295257
H	-6.195798	-7.856783	7.276928
H	-7.029430	-7.472816	8.788299
C	-11.240403	-0.395810	-0.649271
C	-12.124533	-0.237999	-1.725954
C	-11.762365	-0.230299	0.641278
C	-13.460766	0.074541	-1.528663
H	-11.749560	-0.321931	-2.742212
C	-13.101070	0.064450	0.851502
H	-11.116160	-0.378797	1.502034
C	-13.973876	0.224988	-0.233118
H	-14.116632	0.213717	-2.382089
H	-13.482575	0.160287	1.862986
N	-15.334110	0.526393	-0.029100
C	-15.728404	1.373604	1.031693
C	-16.319589	-0.033455	-0.875829
C	-16.858779	1.066093	1.798899
C	-14.999945	2.532538	1.327519
C	-16.247564	-1.377841	-1.260324
C	-17.383340	0.751511	-1.336679
C	-17.252448	1.906041	2.834009
H	-17.424822	0.166769	1.577288
C	-15.391143	3.356173	2.376763
H	-14.128013	2.781165	0.730562
C	-17.216152	-1.918604	-2.098092
H	-15.428735	-1.992173	-0.899113
C	-18.356981	0.196752	-2.159210
H	-17.440046	1.795466	-1.044572
C	-16.520357	3.052515	3.134056
H	-18.131866	1.652348	3.419172
H	-14.814915	4.251481	2.592561
C	-18.278283	-1.138259	-2.549379
H	-17.146139	-2.963399	-2.387225
H	-19.175940	0.819083	-2.508637
H	-16.826700	3.702512	3.947872
H	-19.036746	-1.566005	-3.197807

References

S1. J. E. Slota, E. Elmalem, G. Tu, B. Watts, J. Fang, P. M. Oberhumer, R. H. Friend, and W. T. S. Huck, *Macromolecules*, 2012, *45* (3), pp 1468–1475.

S2. I. S., Lee, S. Y., Adachi, C. and Yasuda, T. (2016), Full-Color Delayed Fluorescence Materials Based on Wedge-Shaped Phthalonitriles and Dicyanopyrazines: Systematic Design, Tunable Photophysical Properties, and OLED Performance. Adv. Funct. Mater., 26: 1813–1821.

S3. Cardona, C. M.; Li, W.; Kaifer, A. E.; Stockdale, D.; Bazan, G. C. Adv. Mater. 2011, 23, 2367−2371.

S4. Gaussian 09, Revision D.01, M. J. Frisch, G. W. Trucks, H. B. Schlegel, G. E. Scuseria, M. A. Robb, J. R. Cheeseman, G. Scalmani, V. Barone, B. Mennucci, G. A. Petersson, H. Nakatsuji, M. Caricato, X. Li, H. P. Hratchian, A. F. Izmaylov, J. Bloino, G. Zheng, J. L. Sonnenberg, M. Hada, M. Ehara, K. Toyota, R. Fukuda, J. Hasegawa, M. Ishida, T. Nakajima, Y. Honda, O. Kitao, H. Nakai, T. Vreven, J. A. Montgomery, Jr., J. E. Peralta, F. Ogliaro, M. Bearpark, J. J. Heyd, E. Brothers, K. N. Kudin, V. N. Staroverov, T. Keith, R. Kobayashi, J. Normand, K. Raghavachari, A. Rendell, J. C. Burant, S. S. Iyengar, J. Tomasi, M. Cossi, N. Rega, J. M. Millam, M. Klene, J. E. Knox, J. B. Cross, V. Bakken, C. Adamo, J. Jaramillo, R. Gomperts, R. E. Stratmann, O. Yazyev, A. J. Austin, R. Cammi, C. Pomelli, J. W. Ochterski, R. L. Martin, K. Morokuma, V. G. Zakrzewski, G. A. Voth, P. Salvador, J. J. Dannenberg, S. Dapprich, A. D. Daniels, O. Farkas, J. B. Foresman, J. V. Ortiz, J. Cioslowski, and D. J. Fox, Gaussian, Inc., Wallingford CT, 2013.

S5. Stephens, P. J.; Devlin, F. J.; Chabalowski, C. F.; Frisch, M. J. J. Phys. Chem. 1994, 98, 11623-11627.

S6. Gao, X.; Bai, S.; Fazzi, D.; Niehaus T.; Barbatti, M.; Thiel, W. J. Chem. Theory Comput. 2017, 13(2), 515.

S7. Becke, A. D. J. Chem. Phys. 1988, 88(4), 2547.

S8. Lee, K.; Kim D. J. Phys. Chem. C 2016, 120, 28330.

S9. Bruhn G.; Davidson E. R.; Mayer I.; Clark A. E. Int. J. Quantum Chem. 2006, 106, 2065.

S10. Q. Zhang, B. Li, S. Huang, H. Nomura, H. Tanaka and C. Adachi, 2014, *Nature Photonics,* 8, 326-332.

S11. F. B. Dias, K. N. Bourdakos, V. Jankus, K. C. Moss, K. T. Kamtekar, V. Bhalla, J. Santos, M. R. Bryce and A. P. Monkman, *Adv. Mater.*, 2013, 25, 3707-3714.

S12 CrysAlisPro, Agil. Technol. Version 1.1 71.35.19 (release 27-10-2011 CrysAlis171.NET) (compiled Oct 27 2011,150211)

S13 H. Nowell, S. A. Barnett, K. E. Christensen, S. J. Teat, D. R. Allan, *J Synchrotron Radiat.*, **2012**, *19*, 435-441

S14 O. V. Dolomanov, L. J. Bourhis, R. J. Gildea, J. A. K. Howard and H. Puschmann, *J. Appl. Crystallogr*. **2009**, *42*, 339-341

S15 G. M. Sheldrick, *Acta Cryst*. **2015**, A71, 3-8

S16 G. M. Sheldrick, *Acta Cryst*. **2015**, C71, 3-8
